# Haplotype-Based Genome-Wide Association Study and Identification of Candidate Genes Associated with Carcass Traits in Hanwoo Cattle

**DOI:** 10.3390/genes11050551

**Published:** 2020-05-14

**Authors:** Swati Srivastava, Krishnamoorthy Srikanth, Sohyoung Won, Ju-Hwan Son, Jong-Eun Park, Woncheoul Park, Han-Ha Chai, Dajeong Lim

**Affiliations:** 1 National Institute of Animal Science, Rural Development Administration, Wanju-gun 55365, Korea; swati051@gmail.com (S.S.); kris87@korea.kr (K.S.); tdpro@naver.com (J.-H.S.); jepark0105@korea.kr (J.-E.P.); wcpark1982@korea.kr (W.P.); hanha@korea.kr (H.-H.C.); 2 Department of Agricultural Biotechnology and Research Institute of Population Genomics, Seoul National University, Seoul 151742, Korea; wsy415@snu.ac.kr

**Keywords:** haplotype, carcass traits, *PLCB1*, *PLCB4*, *CEL*

## Abstract

Hanwoo, is the most popular native beef cattle in South Korea. Due to its extensive popularity, research is ongoing to enhance its carcass quality and marbling traits. In this study we conducted a haplotype-based genome-wide association study (GWAS) by constructing haplotype blocks by three methods: number of single nucleotide polymorphisms (SNPs) in a haplotype block (nsnp), length of genomic region in kb (Len) and linkage disequilibrium (LD). Significant haplotype blocks and genes associated with them were identified for carcass traits such as BFT (back fat thickness), EMA (eye Muscle area), CWT (carcass weight) and MS (marbling score). Gene-set enrichment analysis and functional annotation of genes in the significantly-associated loci revealed candidate genes, including *PLCB1* and *PLCB4* present on BTA13, coding for phospholipases, which might be important candidates for increasing fat deposition due to their role in lipid metabolism and adipogenesis. *CEL* (carboxyl ester lipase), a bile-salt activated lipase, responsible for lipid catabolic process was also identified within the significantly-associated haplotype block on BTA11. The results were validated in a different Hanwoo population. The genes and pathways identified in this study may serve as good candidates for improving carcass traits in Hanwoo cattle.

## 1. Introduction

Hanwoo, the premium beef cattle of South Korea, is well known for its juiciness, texture, and flavor. In order to maintain consumer preferences and profitability carcass and meat quality must be continuously enhanced [1]. Consumers prefer highly-marbled meat, as it increases tenderness and palatability, therefore, identifying markers associated with muscular fat deposition is imperative [2]. Traits such as carcass traits, reproductive traits, milk production traits, and many more can be easily analyzed by genomic region mapping or by identification of quantitative trait locus (QTL). Genomic loci associated with complex traits can be explored through technologies such as SNP (single nucleotide polymorphism) genotyping and high-throughput sequencing, together with GWAS (genome-wide association study). Many studies have explored the effect of SNPs on carcass traits in Hanwoo [3,4,5,6,7,8,9,10,11] including one [3], which explored genetic contribution of exonic SNPs on carcass traits in Hanwoo using linear mixed model and Bayesian networks. They also suggested that a large proportion of genetic variance was contributed by synonymous variants. Unlike single SNP GWAS, the use of haplotype blocks where each block is tagged with an effective SNP (SNP tag) in GWAS, enables high-resolution association mapping [12,13]. The inclusion of haplotypes, instead of SNPs is advantageous, as the effects of individual SNPs is too small to overcome stringent significance thresholds set in GWAS [14]. Haplotypes can also deal with the problem of missing or low heritability [15]. Moreover, a haplotype-based GWAS study, showed significant improvement in capturing phenotypic variance, compared to an SNP-based study [16].

There are certain limitations in using single SNP information, as individual SNPs have small effects, therefore many of them will not reach the stringent significance threshold and there will be incomplete linkage disequilibrium (LD) between genotyped SNPs and casual variants as explained by Aylor et al. [14,17]. However, by incorporating haplotype information, economically-valuable traits and salient breed characteristics can be identified in various livestock [18,19]. It can provide more robust analysis, as it advances resolution of association and facilitates approximation of markers and causal mutation association by increasing LD [20,21,22]. Haplotype-based association analysis is extensively used to identify trait-associated genes in the human genome and it is now increasingly being used in livestock [23,24]. Characterization of haplotype blocks (i.e., combinations of alleles) of the bovine genome can help in understanding and improving economically-important traits [25]. Compared to SNP-based study, haplotypes have remarkably improved the potential of GWAS [26,27]. Genes present in significantly-associated haplotype blocks along with pathway information can be used to improve carcass traits as many genomic regions are unique to the specific population in which they were discovered and sometimes they are not replicated in other breeds. Several studies have identified significantly-associated haplotypes for various traits such as, milk quality in Holstein [15], carcass trait and meat quality in Simmental cattle [14], slick-hair coat in tropically-adapted cattle [28], reproductive traits in Nellore cattle [29], and meat quality traits in Angus cattle [30] by using haplotype-based GWAS and then identifying QTLs and genes present in significant haplotypes. However, presence of non-informative SNPs in long haplotype blocks or small haplotype blocks which ignore adjacent SNP information, may cause erroneous association thereby reducing the effectiveness of the analysis [31]. Therefore, several haplotype-based association studies have used strategies such as sliding window, linkage disequilibrium(LD) [14], and number of SNPs in haplotype blocks [15], for constructing haplotype blocks to map allele-associated traits in plants [32], humans [13,31], swine [33], and cattle [14,15,16,24,26,29,34,35]. Methods based on linkage disequilibrium (LD), can reduce the number of explanatory variables used for computation [34]. LD plays a vital role in prediction of GWAS and also for genomic selection between quantitative trait loci (QTL) and genetic markers [36,37]. 

Therefore, in this study, we constructed haplotypes based on length (size of haplotypes), number of SNPs (in each haplotype block), and LD, and performed association analysis using the linear mixed model implemented in GEMMA (Genome-wide Efficient Mixed Model Association) software [38]. We then identified candidate genes within the significantly-associated haplotype blocks and performed functional annotation to identify molecular pathways enriched with the candidate genes. And finally, we validated the results in a different Hanwoo population. To the best of our knowledge, this is the first haplotype-based GWAS in Hanwoo cattle.

## 2. Material and Methods

### 2.1. Genotype and Phenotype Data

Phenotypic information for 1166 Hanwoo cattle, born between April 2005 and June 2014 in Nonghyup Hanwoo improvement center, National Institute of Animal Science (NIAS), Rural Development Administration (RDA), South Korea, were collected. All the animals used in this study were steers. All the methods and experimental protocols followed in this study were approved by the Animal Care and Use Committee of the National Institute of Animal Science (NIAS), Rural Development Administration (RDA), South Korea. Tissue samples were collected from the carcasses of slaughtered animals, so ethical committee approval was not required. Available information included sire and dam information along with the individual animals’ date of birth, age at slaughter and progeny test score [39]. Animals were slaughtered at approximately 24 months of age. Four carcass traits—back fat thickness (BFT), carcass weight (CWT), eye muscle area (EMA), and marbling score (MS) were analyzed in this study. CWT was measured in kilograms ranging from 158 to 518 kg. BFT was measured in millimeters ranging from 2 to 25 mm, EMA was measured in centimeter square ranging from 22 to 130 cm^2^ and marbling score was assessed on a 1–9-point scale according to the Korean beef marbling standard (KAPE) ranging from 1 to 9 in given data. Descriptive statistics of the four carcass traits for 1161 Hanwoo cattle are given in Table 1 with boxplot for phenotypic traits in Figure S1. A total of 611,799 SNPs was genotyped with the Illumina Bovine SNP700K BeadChip for 898 animals. Quality control was performed through PLINK ver. 1.9 [40]. For further analyses, SNPs having low minor allele frequency (<0.01), low genotyping rate (<0.90), significant deviation from Hardy–Weinberg equilibrium (<0.001) were discarded; only biallelic format was consider for further study. Individuals with low genotyping call rate (<0.95) and missing slaughter age were excluded from the study. After filtering for quality, 887 animals with 547,836 SNPs remained for further analyses.

### 2.2. Construction of Haplotype Blocks

Haplotype blocks were created for 887 animals with 547,836 SNPs. For construction of haplotype blocks, two steps were followed: (1) phasing of chromosomes through SHAPEIT.v2 [41] and (2) construction of haplotype blocks through GHap, package in R.

#### 2.2.1. Phasing of Chromosomes

**Scheme 1:**
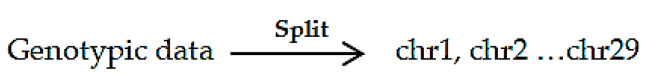
Splitting of Genotypic data (SNP) based on chromosome.

The genotype data was segregated based on chromosomes with creation of individual chromosome ped and map files as explained below (Scheme 1).

The genotypes for each chromosome were phased using SHAPEIT.v2 with 200 states and window size of 0.5 Mb [41]. SHAPEIT was used for estimating haplotypes from either genotypes or sequencing data. This created the haps and sample file which was used to generate haplotype blocks through GHap.

#### 2.2.2. Haplotype Blocks (Haploblocks) Construction

**Scheme 2:**
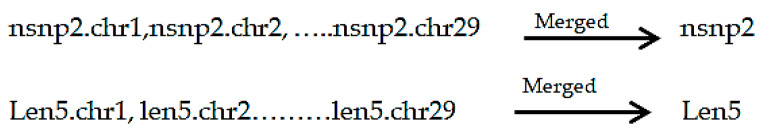
Haplotype blocks constructed based on nsnp, len and LD for each chromosome are merged to form one data set of that particular category. These data set are further used for association study.

Haplotype blocks were constructed based on LD, Len, and nsnp methods and corresponding phase, sample, and marker files for each chromosome were created. The genomic positions in haplotype blocks were based on the distance from the first SNP. After phasing, haplotype blocks were constructed. The blocks were based on: (1) the number of SNP haplotype blocks containing 2, 5, 10, 20, 30, and 50 SNPs per block, respectively, (2) genomic window sizes of 5 kb, 10 kb, 20 kb, 50 kb, 100 kb, and 200 kb, respectively, and (3) LD with r^2^ value of 0.2, 0.3, 0.4, 0.5, 0.6, and 0.7 respectively. A total of 979,942 haplotype blocks were created using the above-mentioned three methods: LD, LEN, and nsnp. We then used GHap (genome-wide haplotyping) package in R to identify haplotype alleles from these haplotypes’ blocks. After construction of haplotypes for each chromosome based on the above categories, all chromosomes were merged as shown below (Scheme 2):

### 2.3. Genome-Wide Association Study (GWAS)

Haplotype alleles determined by GHap were used for association studies using linear mixed model implemented in GEMMA (genome-wide efficient mixed model association) software [38]. As it is computationally efficient in performing large scale GWAS studies. Phenotype and genotype data for 887 animals were used to perform the studies for all four traits: BFT, CWT, EMA, and MS. The model, was used as follows

(1) y=Wα+xβ+μ

where *y* is an n-vector of quantitative traits for n individuals and *W* = (*w*_1_; …..; *w_c_*) is an n x c matrix of covariates (fixed effects). Slaughter age was used as fixed effect in this study, as all animals were steers and reared in same slaughterhouse. Hence, sex and slaughter place are not considered as fixed effects. α is a c vector of the corresponding coefficient, x is a n-vector of haplotype, β is the effect size of the marker, and µ is an n-vector of random effects. GEMMA obtains maximum likelihood estimate (MLE) of and outputs the corresponding *p-*value. These *p*-values were used to filter out the significant haplotype blocks for each trait. Significant haplotype blocks were identified based on a Bonferroni-corrected *p*-value (0.05/979942 = 5.10 × 10^−8^).

### 2.4. Validation

To validate our result, the genotype and phenotype data of a further sample of 468 Hanwoo cattle, reported in a previous study, was used [11]. Similar steps were followed to construct haplotype blocks and to study association analyses as used for the original data set.

### 2.5. Gene Identification in Significant Haplotype Blocks

To identify candidate genes within the associated haplotype blocks, it was necessary to investigate the genomic region. Significant haplotype blocks were scanned from start to end position to identify the genes present. Ensembl database [42] (Ensembl 97: July 2019) with ARS-UCD1.2 was used as the reference genome and detailed gene information was obtained from the NCBI (National Center for Biotechnology Information) [43]. Information such as gene symbol, location, assembly method, gene type, and gene function were retrieved for each gene present in significant haplotype blocks for all four carcass traits (BFT, CWT, EMA, and MS).

### 2.6. Gene Functional Enrichment

After identification of genes present in significant haplotype blocks, the PANTHER classification system [44] was used to classify and cluster genes based on biological process. To understand and identify the biological pathways and the genes involved, gene ontology (GO) enrichment was also performed using DAVID (Database for Annotation, Visualization, and Integrated Discovery) v6.8 [45,46]. Significantly-enriched pathways and GO terms were identified based on enrichment scores.

## 3. Results

### 3.1. Significant Haplotype Blocks

**Figure 1:**
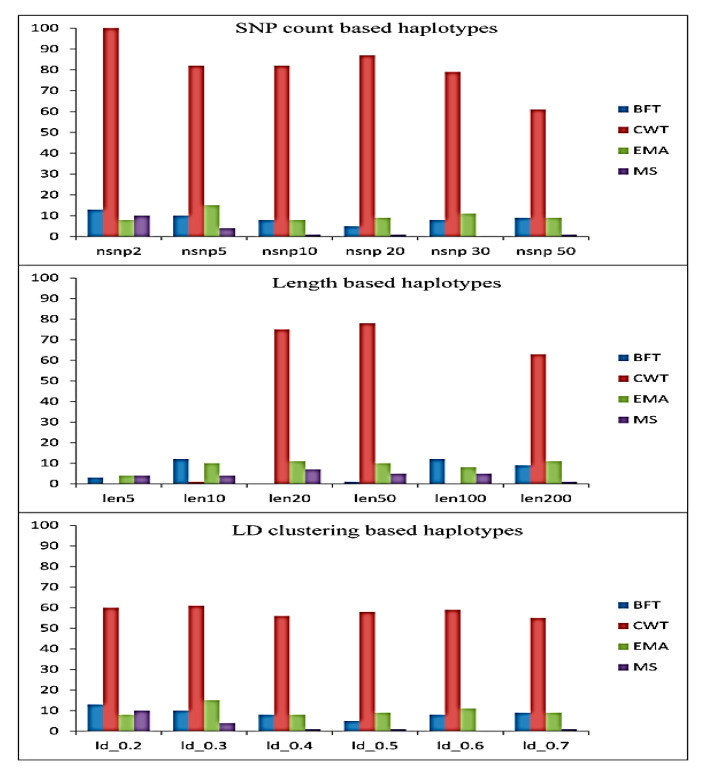
Number of significant haplotype blocks identified from GWAS for the four carcass traits by three different methods. SNP: Single nucleotide polymorphism, nsnp: number of SNPs in each block, Len: length in kilo base pair (Kb), LD: linkage disequilibrium, BFT: back fat thickness, CWT: carcass weight, EMA: eye muscle area, and MS: marbling score.

A total of 1434 haplotype blocks (BFT: 150, CWT: 1062, EMA: 161, and MS: 61) were identified as significantly-associated haplotype blocks based on this threshold (Figure 1). Detail tabulated description of each haplotype block for four carcass traits (BFT, CWT, EMA and MS) is provided in Table S1. Most significant regions were detected on BTA14 for CWT; BTA11 and BTA13 for BFT; BTA1, BTA6, BTA9 and BTA12 for MS; BTA1, and BTA10 for EMA. Manhattan plot for four carcass traits by all three method are provided in Table S2.

### 3.2. Candidate Gene Identification

**Figure 2:**
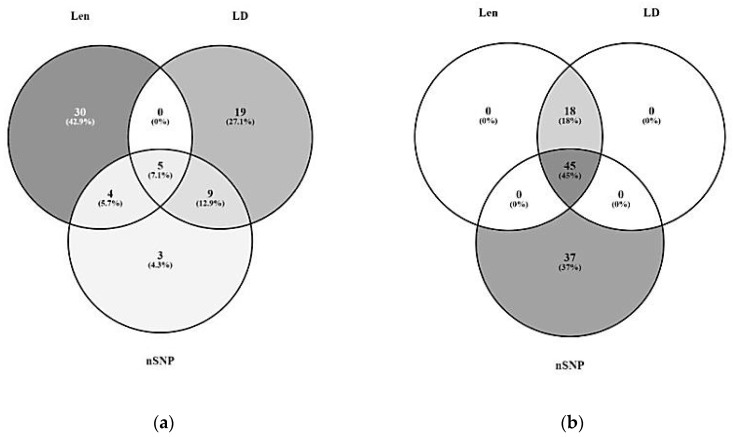

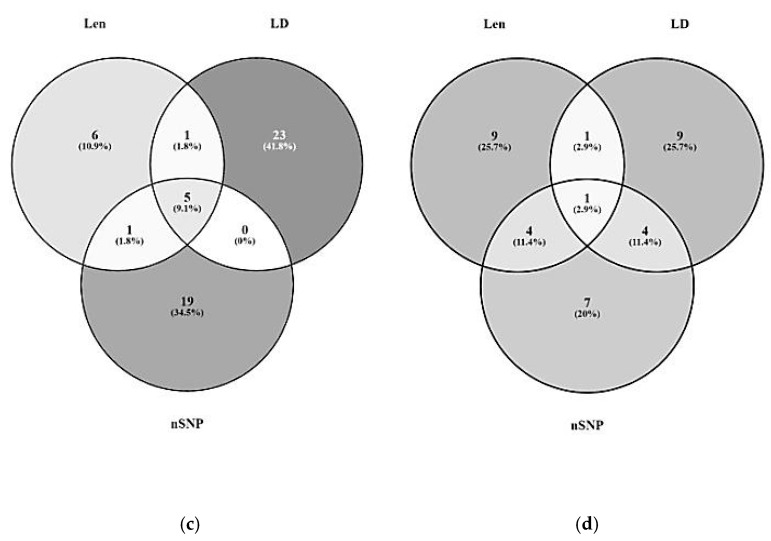
Number of genes unique and common among three methods for (**a**) BFT, (**b**) CWT, (**c**) EMA, and (**d**) MS.

Genes within the significant haplotype blocks were identified using Ensembl database for all three methods (LD, Len, and nsnp) for each trait. Venny 2.1.0 [47] revealed the number of genes which are unique or common among three methods for all carcass traits (Figure 2, Table S3).

Unique and common trait-specific genes were analyzed for each trait and are listed along with function in Table 2. It can be observed that for BFT only five genes were identified by all three methods whereas a greater number of unique genes was identified by the Len followed by the LD methods. *PLCB1* and *TMX4* were some of the genes identified by all three methods whereas *PLCB4,* is identified only by Len and LD methods and the *CEL* gene i.e., carboxyl ester lipase, by the LD method. For CWT, the number of common genes detected by all three methods was 45% of total genes and these included *LYPLA1*, *TMEM68*, *XKR9*, *PREK2*, *KLHL38*, *TOX* and *CYP7A1*, and *SOX17* and *PRKDC,* all present on BTA14. *MYC* and *FAM110B* genes responsible for the cell cycle and cell progression were detected only by the nsnp method.

For EMA, *SLC8A3* was detected by all three methods while *COL18A1* and *RORA* were detected by only LD and nsnp methods, respectively. MS showed only *FYN* gene as common, whereas four genes were common between LD and nsnp method including *PGM2*. *RB1* and *PAPR14* emerged as unique gene detected only by Len and LD methods, respectively.

### 3.3. Gene Function Annotation

**Figure 3:**
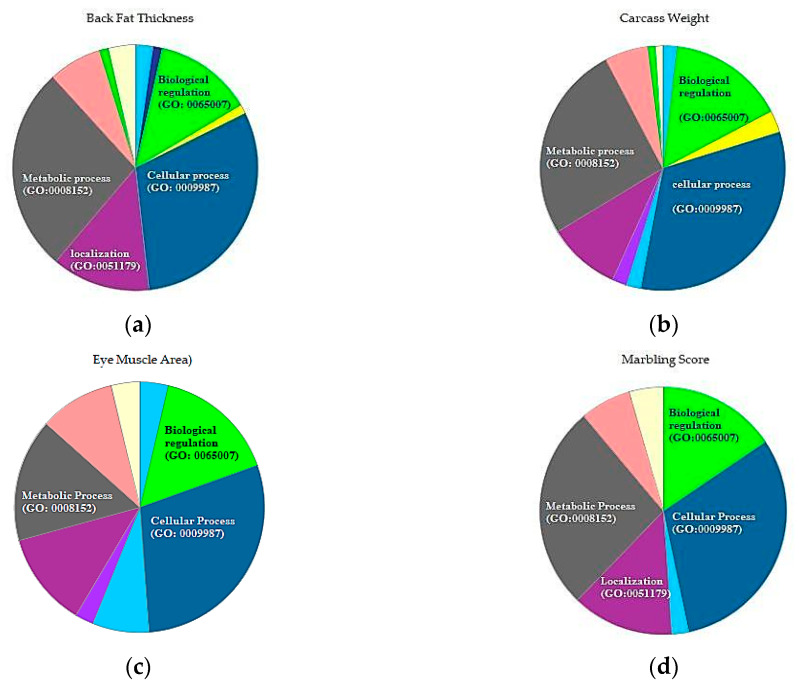
PANTHER analysis of the co-association network for (**a**) BFT, (**b**) CWT, (**c**) EMA, and (**d**) MS.

PANTHER was used to functionally annotate genes based on biological, cellular, and metabolic processes as in Figure 3. Further, all the genes identified by the three methods were functionally annotated via DAVID. DAVID clusters the gene dataset based on the enrichment score (Table S4, Table S5(a–d)). The higher the enrichment score, the more significant the cluster. The result in Table 3 shows that genes such as *CEL, PLCB1, PLCB4,* present in significant haplotype blocks are mainly associated with the lipid catabolic process (GO: 0016042), insulin secretion (bta04911), the glucagon-signaling pathway (bta04922), insulin secretion by pancreas (bta04911), glutamatergic synapses (bta04724), and the sphingolipid-signaling pathway (bta04071) etc., which are involved in lipid metabolism and therefore can help in increasing the richness of meat. *RORA*, helps in regulation of fat cell differentiation (GO: 004559). Meat quality is determined by fat, protein, and cell mass. Several pathways that might play a role in increasing cell mass and size were identified by DAVID and included cell cycle (bta04110), negative regulation of G1/S transition of the mitotic cell (GO: 2000134), potassium ion trans-membrane transportation (GO: 0071805), and regulation of microtubule cytoskeleton organization (GO: 0070507). Several other related GO terms were also identified (Table 3).

## 4. Discussion

The association study by GEMMA software revealed that BTA 13 and BTA11 are significant haplotype blocks for BFT as they contain markers which may affect candidate genes such as *PLCB1*, *PLCB4,* and *CEL*. Figure 2 highlights that there were numerous genes present in significant haplotype blocks, some identified commonly by all three methods, some by two methods, and others were uniquely detected by single method. BTA14 was found to have most of the significantly-associated haplotype block for CWT (Figure 1) which agrees with previous reports [3,5]. It contains a greater number of common genes extracted by all three methods—approximately 45% of total genes, as seen in Figure 2b. Table 4 highlights the candidate genes found in significantly-associated haplotype blocks. BTA 12 was found to be more significant for EMA. BTA 12, by the LD method, BTA 9 and BTA 12, by the Len method, and BTA 3 and BTA 12, by the nsnp method, were found to be more significant for MS, as can be seen through the Manhattan plot (Table S2) and *p*-value generated by GEMMA software. The significant haplotype blocks identified in this study were validated in a different population of 468 Hanwoo cattle (Table 4). The significance threshold for the validation population was 3.45 × 10^−8^. SNPs in BTA13 and BTA11 that harbored the majority of the haplotype blocks and candidate genes significant to this study were found to be associated with carcass traits in the test population (887 original data).

Functional annotation analyses with PANTHER and DAVID, showed that most of the identified genes were found to function in cellular and metabolic processes. Genes which are involved in increasing cell mass, cell number, protein formation, and stabilization and hence are mass determinant are listed in Table 3 [2]. Cellular processes such as hyperplasia and hypertrophy are responsible for increasing muscle mass [2]. Hypertrophy can be increased by increased protein synthesis. *LYPLA1*, *TMEM68*, and *XKR9* which were found within the significantly-associated haplotype blocks were identified as candidate genes for improving carcass traits in a previous study [52]. These genes are responsible for lipase activity and weight gain in cattle. *LYPLA1* gene has hydrolase and lipase activity. *TMEM68* is expressed in bovine rumen, abomasum, intestine and adipose tissue in cattle, and likely affects lipid biosynthetic processes. *XKR9* promotes phosphatidyl serine exposure on apoptotic cell surface. *KLHL38* gene plays an important role in maintaining the balance between intermediate filament stability, a process in maintaining skin integrity. It is an important component of ubiquitin-protein ligase complex also formed by *KLHl2* [52] responsible for proteasomal degradation. Previous studies [3,5] had reported that *TOX* and *CYP7A1* were functional genes for CWT in Hanwoo cattle. Pausch et al. [55] identified *SOX17* as positional and functional gene involved in transcriptional regulation via inhibiting Wnt signaling. RB1 [58,59] plays an important role in posttranslational modifications and hence regulates cell cycle. These genes are responsible for cell development and proliferation. Another set of gene detected were involved in muscular growth such as *KLHL38* (filament stability) and *KCNQ3*, *KCNB2*, and *KCNIP4* [30] which play an important role in calcium and potassium transport and in meat tenderization through their involvement in the proteolytic system responsible for postmortem tenderization and muscle contraction and these were among the candidate genes.

**Figure 4:**
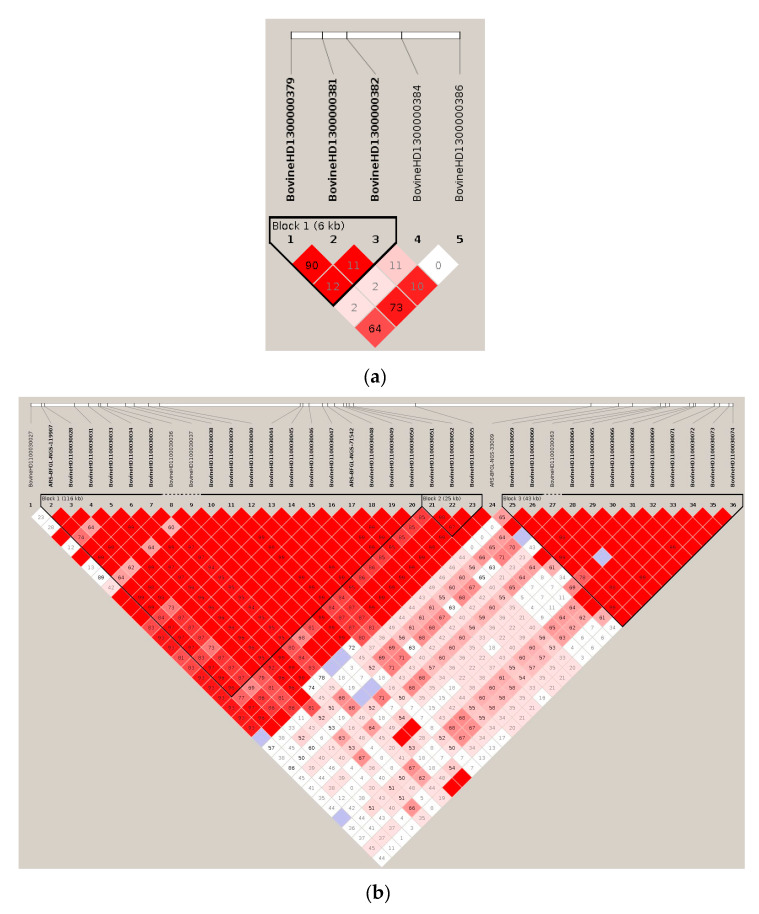

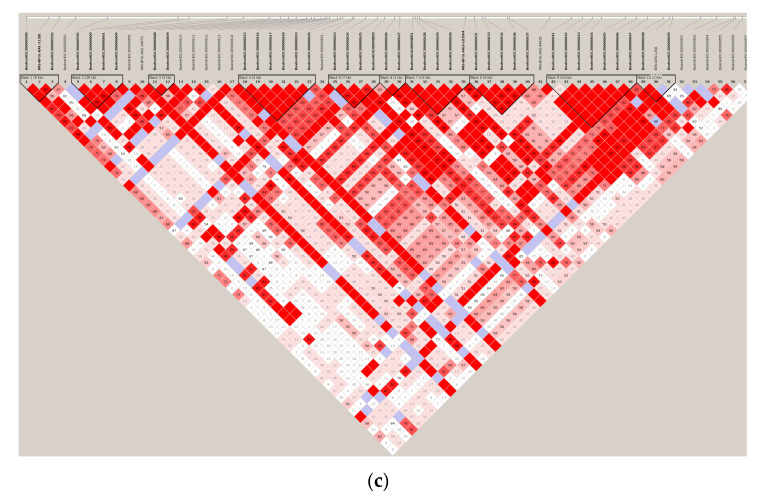
Haplotype structures viewed using Haploview for carcass traits. (**a**) Top haploblock on BTA13 with *p*-value of 5.27 × 10^−6^, from nsnp5 method which included *PLCB1* gene, (**b**) top haplotype block from LD 0.2 method on BTA11 with *p*-value of 5.16 × 10^−6^, which included the *CEL* gene, and (**c**) top haplotype block from Len200 method on BTA13 with *p*-value of 3.02 × 10^−6^ which included the *PCLB4* gene.

A study by Zhao et al. [60], suggested that modulation of annexin expression in animals with high BFT may play an important role in fat formation in beef cattle. These annexins are calcium-dependent phospholipid-binding proteins involved in inhibition of phospholipase activity, exocytosis, endocytosis, signal transduction, and many other biological processes. As can be seen through the PANTHER result there is a set of lipase genes that participates in metabolic processes and plays a significant role in formation of kinases and hence metabolism of the lipid molecule. Through our study *PLCB1*, phospholipase C Beta 1, and *PLCB4* phospholipase C Beta 4 [48,61] were identified as important phospholipases by 12 haplotype blocks and three haplotype blocks, respectively, as seen in Table 4. Haploview analysis of top haplotype blocks (based on *p*-value) for each candidate gene is shown in Figure 4. There were five SNPs contributing to top haplotype block, nsnp5 with block id 68104 for *PLCB1* gene. There were 58 SNPs present in haplotype block Len200 with block id: 7021 affecting gene *PLCB4*. These phospholipases play an essential role in lipid metabolism as fat deposition plays an important role in increasing richness in meat and it has been reported that very low levels of intramuscular fat can lead to dry and less-tasty meat [62]. *CEL* gene i.e., carboxyl ester lipase is a bile-salt-activated lipase, detected by only one haplotype block with block id: 18600 constructed by LD0.2 method with haplotype block start position 103051196 to end 103319019 with 36 SNPs (Figure 4). These three genes are responsible for the lipid catabolic-process glucagon signaling pathway, metabolic pathways, and glutamatergic pathways, etc., as can be seen in Table 3. The *CYP7B1* gene present on BTA 14 also has a minor role in bile acid synthesis [54] which plays an essential role in lipid metabolism in intestines of animal.

### Phospholipase

Important phospholipase C β (*PLCB*) isoforms were identified in this study i.e., *PLCB1* and *PLCB4*. These genes code for 1-phosphatidylinositol 4, 5-bisphosphate phosphodiesterase proteins which are responsible for the hydrolysis of phospholipids [48]. As discussed above fat deposition is governed by the cell mass and cell size and these phospholipids play an important role in forming major components of the cell membrane and determining the physical and chemical properties of the cell [63]. When these phospholipids are acted upon by phospholipase C they form diglyceride (DG) and inositol phosphates for Ca^2+^, which is an activator of protein kinase C [64]. They are further responsible for catabolism of lipid and metabolism of membrane structural phospholipids [65] (GO: 0016042) as seen with the DAVID with *p* < 0.02. Their catabolic activity can reduce the fat deposition in muscle, making meat dry and lean. The genes coding for phospholipase are also involved in the sphingolipid signaling pathway (bta04071). Sphingolipids protect the cell surface from the harmful outside environment and help in making the cell mechanically stable. On the positive side, phospholipase governs the interaction of glucose, acetylcholine, and free fatty acids on insulin secretion (bta04911, bta04972: pancreatic secretion) as they increase insulin secretion leading to increased storage of glucose in body cells [66] and suppressing its counter-regulatory pathway i.e., the glucagon signaling pathway (bta04922) and other metabolic pathways (bta01100). Studies also showed that in ruminants, the adipose tissue is the principal site of de novo fatty acid (FA) synthesis [67,68] where glucose is the main substrate for lipogenic adipocytes in muscle [69].

## 5. Conclusions

*PLCB1*, *PLCB4,* and *CEL* genes were identified as putative candidate genes, as they regulate lipid metabolism which is responsible for increasing fat thickness and the weight of an organism, and hence more rich and marbled meat. To our knowledge, until now, no work has been reported which showed involvement of *PLCB1*, *PLCB4,* and *CEL* in increasing the carcass traits of an animal. Also, we can conclude that the LD and nsnp methods are more useful in the construction of haplotype blocks as a bigger number of unique genes are obtained by these two methods. For example, *CEL* was only detected by the LD method and *PLCB4* only detected by the nsnp and Len methods. Alteration in expression of these lipase-forming genes can help in improving Hanwoo beef quality by increasing muscular fat thickness leading to enhancement of economic growth.

## Figures and Tables

**Table 1:** Descriptive statistics for four carcass traits in 1161 Hanwoo cattle.

Trait	Mean	SD	Min	Max
BFT (mm)	8.92	3.65	2	25
CWT (kg)	369.16	41.36	158	518
EMA (cm^2^)	82.75	9.25	22	130
MS (1–9)	3.86	1.65	1	9

SD: Standard Deviation, Min: Minimum, Max: Maximum, BFT: back fat thickness, CWT: carcass weight, EMA: eye muscle area, and MS: marbling score.

**Table 2:** List of genes related to carcass trait identified by different haplotype methods and their functions.

Trait	BTA	Genes	Detected by Method	Function
BFT	13	*PLCB1*	LD, Len, nsnp	Lipid metabolism [48]
	13	*TMX4*	LD, Len, nsnp	Thioredoxin-related trans membrane protein 4, responsible for negative regulation of hormone secretion [49]
	13	*PLCB4*	LD, Len	Phospholipase C Beta 4 [50] involved in lipid metabolism [48]
	11	*CEL*	LD	Carboxyl ester lipase (CEL) is a bile-salt-activated lipase. It is also responsible for lipid catabolic process [51]
CWT	14	*LYPLA1*	LD, Len, nsnp	Hydrolase and lipase activity [52]
	14	*TMEM68*	LD, Len, nsnp	Expressed in bovine rumen, abomasum, intestine, and adipose tissue in cattle, and likely affects lipid biosynthetic processes [52]
	14	*XKR9*	LD, Len, nsnp	Belongs to Xkr family possessing caspases recognition site, responsible for apoptosis [52,53]
	14	*PREK2*	LD, Len, nsnp	GTPase activator, an important paralog of PREX1 responsible for increasing carcass weight in Hanwoo cattle. It also aids in meat development with richness in texture and flavor [9]
	14	*KLHL38*	LD, Len, nsnp	Helps in maintaining the balance between intermediate filament stability, a process in maintaining skin integrity. It is an important component of ubiquitin–protein–ligase complex also formed by KLHl2 [30]
	14	*TOX*	LD, Len, nsnp	Responsible for the molecular regulation of puberty in Brahman cattle [3,5]
	14	*CYP7B1*	LD, Len, nsnp	Oxysterol 7-hydroxylase, involved in bile acid synthesis [54]
	14	*MYC*	nsnp	Myelocytomatosis oncogene, responsible for ribosomal and mitochondrial biogenesis, glucose and glutamine metabolism, lipid synthesis, and cell cycle progression [2,3].
	13	*FAM110B*	nsnp	A paralog of FAM110A gene, which is responsible for cell cycle progression through G1 phase and hence might play a role in increasing carcass weight in Hanwoo cattle by increasing cell number and cell size [5]
	14	*SOX17*	LD, Len, nsnp	It [55] is a positional and functional gene involved in transcriptional regulation via inhibiting Wnt signaling (GO: 0060070). These are also responsible for tyrosine protein kinase and postnatal angiogenesis and hence affect growth-related traits in cattle
	14	*PRKDC*	LD, Len, nsnp	Plays an important role in cell cycle progression, apoptosis, and cellular transformation(bta04110) [3]
EMA	10	*SLC8A3*	LD, Len, nsnp	Promotes easy uptake of protein for muscular building and fibrillar collagen building [56].
	1	*COL18A1*	LD	
	10	*RORA*	nsnp	RAR-related orphan receptor, responsible for cholesterol homeostasis (GO: 0042632) and negative regulation of fat cell differentiation (GO: 0045599)
MS	9	*FYN*	LD, Len, nsnp	It belongs to member of Src family of non-receptor tyrosine kinases; This gene plays an important role in increasing adipogenesis [4].
	1	*PARP14*	Len	It belongs to poly (ADP-ribose) polymerase family and, it play key role in regulating adipogenesis [9].
	6	*PGM2*	LD, nsnp	It is involved in galactose metabolism, which can help in adipogenesis in an organism [57]
	12	*RB1*	LD	It plays an important role in posttranslational modifications and hence regulates cell cycle (GO: 2000134) [58,59]

**Table 3:** DAVID functional annotation for genes and their associations with pathways.

Term	Pathway	Count	%	*p-Value*	Genes
**Back Fat Thickness**					
GO: 0016042	Lipid catabolic process	3	4.76	0.02	*CEL*, *PLCB4*, *PLCB1*
bta04911	Insulin secretion	3	4.76	0.03	*PLCB4*, *PLCB1*, *SNAP25*
bta03015	mRNA surveillance pathway	3	4.76	0.035	*UPF2*, *MAGOH*, *MSI1*
bta04922	Glucagon signaling pathway	3	4.76	0.039	*LDHA*, *PLCB4*, *PLCB1*
GO: 0005975	Carbohydrate metabolic process	3	4.76	0.039	*LDHA*, *GBGT1*, *GLT6D1*
bta04972	Pancreatic secretion	3	4.76	0.04	*CEL*, *PLCB4*, *PLCB1*
bta01100	Metabolic pathways	9	14.29	0.04	*CEL*, *LDHA*, *DHFR*, *PLCB4*, *GBGT1*, *SPTLC3*, *AOX4*, *PLCB1*, *NANP*
bta04724	Glutamatergic synapse	3	4.76	0.053	*PLCB4*, *SLC1A7*, *PLCB1*
bta04071	Sphingolipid signaling pathway	3	4.76	0.059	*PLCB4*, *SPTLC3*, *PLCB1*
**Carcass Weight**					
GO: 0031648	Protein destabilization	4	3.92	0.001	*DERL1*, *RNF139*, *PRKDC*, *SOX17*
bta04110	Cell cycle	4	3.92	0.021	*PRKDC*, *CDK6*, *MCM4*, *MYC*
GO: 0071805	Potassium ion trans membrane transport	3	2.94	0.034	*KCNQ3*, *KCNB2*, *KCNIP4*
GO: 0071498	Cellular response to fluid shear stress	2	1.96	0.051	*MTSS1*, *HAS2*
GO: 0060070	Canonical Wnt signaling pathway	3	2.94	0.059	*SFRP5*, *SOX17*, *MYC*
GO: 0034765	Regulation of ion trans membrane transport	2	1.96	0.089	*KCNQ3*, *KCNB2*
GO: 0090090	Negative regulation of canonical Wnt signaling pathway	3	2.94	0.09	*SFRP5*, *RGS20*, *SOX17*
**Eye Muscle Area**					
GO: 0002092	Positive regulation of receptor internalization	3	6.25	0.001	*SCYL2*, *WNT3A*, *SYNJ2BP*
GO: 0021766	Hippocampus development	3	6.25	0.003	*WNT3A*, *PAFAH1B1*, *YWHAE*
GO: 0010988	Regulation of low-density Lipoprotein particle clearance	2	4.17	0.008	*CNPY2*, *NR1H4*
GO: 2000188	Regulation of cholesterol Homeostasis (replaced by GO: 0042632)	2	4.17	0.018	*RORA*, *NR1H4*
GO: 0070507	Regulation of microtubule Cytoskeleton organization	2	4.17	0.037	*WNT3A*, *PAFAH1B1*
GO: 0006605	Protein targeting	2	4.17	0.04	*SYNJ2BP*, *YWHAE*
GO: 0043124	Negative regulation of I-kappaB Kinase/NF-kappaB signaling	2	4.17	0.078	*RORA*, *NR1H4*
GO: 0043087	Regulation of GTPase activity	2	4.17	0.083	*PAFAH1B1*, *CRK*
GO: 0045599	Negative regulation of fat cell differentiation	2	4.17	0.083	*WNT3A*, *RORA*
**Marbling Score**					
bta04110	Cell cycle	3	8.57	0.022	*RB1*, *CHEK2*, *CUL1*
GO: 2000134	Negative regulation of G1/S Transition of mitotic cell cycle	2	5.71	0.024	*EZH2*, *RB1*
GO: 0006915	Apoptotic process	3	8.57	0.031	*RRAGA*, *RB1*, *CUL1*
GO: 0042752	Regulation of circadian rhythm	2	5.71	0.043	*EZH2*, *PPARG*

**Table 4:** Candidate genes identified by different haplotype blocks constructed by three different methods and their *p*-value obtained by GEMMA software.

Trait	Chromosome	Gene	Gene Position	Method	Haplotype Block ID	Haplotype Block Start	Haplotype Block End	*p*-Value	Validation Result
BFT	13	PLCB1	NC_037340.1 (933195–1796412)	LD0.2	20,062	1,440,165	1,484,853	7.39 × 10^−6^	3.39 × 10^−2^
				LD0.3	25,710	1,488,714	1,520,870	7.86 × 10^−6^	8.61 × 10^−4^
				LD0.4	30,888	1,582,812	1,622,566	7.29 × 10^−6^	1.16 × 10^−3^
				Len200	7071	1,400,001	1,600,001	5.56 × 10^−6^	4.16 × 10^−3^
				Len100	13,990	1,400,001	1,500,001	6.01 × 10^−6^	1.77 × 10^−2^
					13,991	1,500,001	1,600,001	5.79 × 10^−6^	2.87 × 10^−3^
				nsnp10	34,050	1,555,543	1,582,812	5.43 × 10^−6^	2.10 × 10^−2^
					34,051	1,588,829	1,627,590	7.32 × 10^−6^	1.02 × 10^−2^
				nsnp20	17,020	1,444,917	1,504,649	5.96 × 10^−6^	3.16 × 10^−3^
				nsnp30	11,346	1,444,917	1,529,072	7.2 × 10^−6^	5.62 × 10^−3^
				nsnp5	68,104	1,564,591	1,582,812	5.27 × 10^−6^	1.14 × 10^−2^
				nsnp50	6806	1,299,046	1,480,919	7.89 × 10^−6^	1.03 × 10^−2^
	13	PLCB4	NC_037340.1 (2021085-2503509)	Len200	7021	2,200,001	2,400,001	3.02 × 10^−6^	9.59 × 10^−3^
				nsnp50	6811	2,172,478	2,350,887	3.86 × 10^−6^	9.46 × 10^−3^
				Len100	13,990	1,400,001	1,500,001	6.01 × 10^−6^	1.77 × 10^−2^
	11	CEL	NC_037338.1 (103058913–103067774)	LD0.2	18,600	103,051,196	103,319,019	5.16 × 10^−6^	1.40 × 10^−1^

## Supplementary Materials

The following are available online at https://www.mdpi.com/2073-4425/11/5/551/s1: Table S1: Significant haploblocks identified through GEMMA; Table S2: Manhattan plot for 887 for association study through GEMMA for all methods; Figure S1: Boxplot for phenotype used in this study; Table S3: Common genes identified by all three methods: Len, LD, nsnp, Table S4: DAVID functional annotation cluster for genes and their pathways; Table S5(a–d). DAVID functional annotation chart summary of significant genes for four carcass traits identified based on p-value.
