# Supplementary material for: Haplotype-Based Genome-Wide Association Study and Identification of Candidate Genes Associated with Carcass Traits in Hanwoo Cattle
Source: Genes (Basel). 2020 May 14;11(5):551. doi: 10.3390/genes11050551 (PMC7290854; doi:10.3390/genes11050551)
Supplement: Supplementary file 1 [file genes-11-00551-s001.zip › Table 5a-d.docx]

Table S5(a) DAVID functional annotation chart summary of significant genes for back fat thickness identified based on p value through DAVID tool.

| Gene | Chromosome | UP_Keywords | P value | KEGG pathway/ GO Term | P value |
| --- | --- | --- | --- | --- | --- |
| PLCB4 | Chr. 13 | Lipid degradation | 0.02 | bta04972:Pancreatic secretion | 0.04 |
|  |  | Lipid metabolism | 0.04 | bta04911:Insulin secretion | 0.03 |
|  |  |  |  | bta01100:Metabolic pathways | 0.04 |
|  |  |  |  | bta04922:Glucagon signaling pathway | 0.04 |
|  |  |  |  | bta04071:Sphingolipid signaling pathway | 0.06 |
|  |  |  |  | bta04724:Glutamatergic synapse | 0.05 |
|  |  |  |  | GO:0004435~phosphatidylinositol phospholipase C activity | 0.05 |
| PLCB1 | Chr. 13 | Lipid degradation | 0.02 | bta04972:Pancreatic secretion | 0.04 |
|  |  | Lipid metabolism | 0.04 | bta04911:Insulin secretion | 0.03 |
|  |  |  |  | bta01100:Metabolic pathways | 0.04 |
|  |  |  |  | bta04922:Glucagon signaling pathway | 0.04 |
|  |  |  |  | bta04724:Glutamatergic synapse | 0.05 |
|  |  |  |  | bta04071:Sphingolipid signaling pathway | 0.06 |
|  |  |  |  | GO:0004435~phosphatidylinositol phospholipase C activity | 0.05 |
| CEL | Chr. 11 | Lipid degradation | 0.02 | bta04972:Pancreatic secretion |  |
|  |  | Lipid metabolism | 0.04 | bta01100:Metabolic pathways | 0.04 |
| CPT2 | Chr. 3 | Lipid metabolism | 0.04 |  |  |
| SNAP25 | Chr. 13 |  |  | bta04911:Insulin secretion | 0.03 |
| UPF2 | Chr. 13 |  |  | bta03015:mRNA surveillance pathway | 0.03 |
| MAGOH | Chr. 3 |  |  | bta03015:mRNA surveillance pathway | 0.03 |
| MSI1 | Chr. 17 |  |  | bta03015:mRNA surveillance pathway | 0.03 |
| LDHA | Chr. 29 |  |  | bta04922:Glucagon signaling pathway | 0.04 |
|  |  |  |  | bta01100:Metabolic pathways | 0.04 |
|  |  |  |  | GO:0005975~carbohydrate metabolic process | 0.04 |
| GBGT1 | Chr. 11 |  |  | bta01100:Metabolic pathways | 0.04 |
|  |  |  |  | GO:0005975~carbohydrate metabolic process | 0.04 |
| GLT6D1 | Chr. 11 |  |  | GO:0005975~carbohydrate metabolic process | 0.04 |
| DHFR | Chr. 7 |  |  | bta01100:Metabolic pathways | 0.04 |
| SPTLC3 | Chr. 13 |  |  | bta01100:Metabolic pathways | 0.04 |
|  |  |  |  | bta04071:Sphingolipid signaling pathway | 0.06 |
| AOX4 | Chr. 2 |  |  | bta01100:Metabolic pathways | 0.04 |
| NANP | Chr. 13 |  |  | bta01100:Metabolic pathways | 0.04 |
| SLC1A7 | Chr. 3 |  |  | bta04724:Glutamatergic synapse | 0.05 |

Table S5 (b). DAVID functional annotation chart summary of significant genes for Carcass weight (CWT) identified based on p value through DAVID tool.

| **Gene** | **Chromosome** | **UP_KEYWORDS** | **P value** | **KEGG pathway/ GO Term** | **P value** |
| --- | --- | --- | --- | --- | --- |
| KCNQ3 | Chr. 14 | Potassium channel | 0.02 |  |  |
|  |  | Potassium transport | 0.05 |  |  |
| KCNB2 | Chr. 14 | Potassium channel | 0.02 |  |  |
|  |  | Potassium transport | 0.05 |  |  |
| KCNIP4 | Chr. 14 | Potassium channel | 0.02 |  |  |
|  |  | Potassium transport | 0.05 |  |  |
| PRKDC | Chr. 14 |  |  | bta04110:Cell cycle | 0.02 |
|  |  |  |  | GO:0031648~protein destabilization | 6.77E-04 |
|  |  |  |  | bta03450:Non-homologous end-joining | |
| CDK6 | Chr. 14 |  |  | bta04110:Cell cycle | 0.02 |
| MCM4 | Chr. 14 |  |  | bta04110:Cell cycle | 0.02 |
| MYC | Chr. 14 |  |  | bta04110:Cell cycle | 0.02 |
| PAN2 | Chr. 14 | Nuclease | 0.06 | bta03018:RNA degradation, | |
| TATDN1 | Chr. 14 | Nuclease | 0.06 |  |  |
| LACTB2 | Chr. 14 | Nuclease | 0.06 |  |  |
| RNF139 | Chr. 14 |  |  | GO:0031648~protein destabilization | 6.77E-04 |
| SOX17 |  |  |  | GO:0031648~protein destabilization | 6.77E-04 |
| DERL1 |  |  |  | GO:0031648~protein destabilization | 6.77E-04 |
| RAB2A | Chr. 14 |  |  | bta04152:AMPK signaling pathway, | |
| CS | Chr. 14 |  |  | bta00020:Citrate cycle (TCA cycle),bta00630:Glyoxylate and dicarboxylate metabolism,bta01100:Metabolic pathways,bta01130:Biosynthesis of antibiotics,bta01200:Carbon metabolism,bta01210:2-Oxocarboxylic acid metabolism,bta01230:Biosynthesis of amino acids, | |
| DERL1 | Chr. 14 |  |  | bta04141:Protein processing in endoplasmic reticulum,bta05014:Amyotrophic lateral sclerosis (ALS), | |
| GGH | Chr. 14 |  |  | bta00790:Folate biosynthesis, | |
| GLS2 | Chr. 14 |  |  | bta00220:Arginine biosynthesis,bta00250:Alanine, aspartate and glutamate metabolism,bta00471:D-Glutamine and D-glutamate metabolism,bta01100:Metabolic pathways,bta04724:Glutamatergic synapse,bta04727:GABAergic synapse,bta04964:Proximal tubule bicarbonate reclamation,bta05206:MicroRNAs in cancer,bta05230:Central carbon metabolism in cancer, | |
| LYPLA1 | Chr. 14 |  |  | bta00564:Glycerophospholipid metabolism,bta05231:Choline metabolism in cancer, | |

Table S5(c). DAVID functional annotation chart summary of significant genes for Eye muscle area (EMA) identified based on p value through DAVID tool.

| Gene | Chromosome | UP_KEYWORDS | P value | KEGG pathway/ GO Term | P value |
| --- | --- | --- | --- | --- | --- |
| PTGES3 | Chr. 5 | Acetylation | 0.004 |  |  |
| MED6 | Chr. 10 | Acetylation | 0.004 |  |  |
|  |  | Activator |  |  |  |
| ACTG2 | Chr. 11 | Acetylation | 0.004 |  |  |
| MYO1C | Chr. 19 | Acetylation | 0.004 |  |  |
| PITPNA | Chr. 19 | Acetylation | 0.004 |  |  |
| CS | Chr. 5 | Acetylation | 0.004 |  |  |
| SNX2 | Chr. 7 | Acetylation | 0.004 |  |  |
| RBMS2 | Chr. 5 | Acetylation | 0.004 |  |  |
| PAFAH1B1 | Chr. 19 | Acetylation | 0.004 |  |  |
| YWHAE | Chr. 19 | Acetylation | 0.004 | GO:0006605~protein targeting | 0.04 |
| NR1H4 | Chr. 5 | Acetylation | 0.004 | GO:0010988~regulation of low-density lipoprotein particle clearance | 0.01 |
|  |  | Activator |  | GO:2000188~regulation of cholesterol homeostasis | 0.02 |
| CNPY2 | Chr. 5 |  |  | GO:0010988~regulation of low-density lipoprotein particle clearance | 0.008 |
| RORA | Chr. 10 |  |  | GO:2000188~regulation of cholesterol homeostasis | 0.02 |
| SYNJ2BP | Chr. 10 |  |  | GO:0006605~protein targeting | 0.04 |
| STAT2 | Chr. 5 | Activator | 0.08 |  |  |

Table S5(d). DAVID functional annotation chart summary of significant genes for marbling score (MS) identified based on p value through DAVID tool.

| Gene | Chromosome | UP_KEYWORDS | P value | KEGG pathway/ GO Term | P value |
| --- | --- | --- | --- | --- | --- |
| TRAF3IP1 | Chr. 3 |  |  | GO:1901621~negative regulation of smoothened signaling pathway involved in dorsal/ventral neural tube patterning | 0.008 |
| SUFU | Chr. 26 |  |  | GO:1901621~negative regulation of smoothened signaling pathway involved in dorsal/ventral neural tube patterning | 0.008 |
| HSPBAP1 | Chr. 1 | Alternative splicing | 0.01 |  |  |
| PLIN2 | Chr. 8 | Alternative splicing | 0.01 |  |  |
| PPARG | Chr. 22 | Alternative splicing | 0.01 |  |  |
| ATP6V1H | Chr. 14 | Alternative splicing | 0.01 |  |  |
| RB1 | Chr.. 12 |  |  | bta04110:Cell cycle | 0.02 |
|  |  |  |  |  |  |
| CHEK2 | Chr. 17 |  |  | bta04110:Cell cycle | 0.02 |
| CUL1 | Chr. 4 |  |  | bta04110:Cell cycle | 0.02 |
| EZH2 | Chr. 4 |  |  | GO:2000134~negative regulation of G1/S transition of mitotic cell cycle | 0.02 |
| RRAGA | Chr. 8 |  |  | GO:0006915~apoptotic process | 0.03 |
