# Supplementary material for: Haplotype-Based Genome-Wide Association Study and Identification of Candidate Genes Associated with Carcass Traits in Hanwoo Cattle
Source: Genes (Basel). 2020 May 14;11(5):551. doi: 10.3390/genes11050551 (PMC7290854; doi:10.3390/genes11050551)
Supplement: Supplementary file 1 [file genes-11-00551-s001.zip › Table S2.docx]

1. Manhattan for BFT


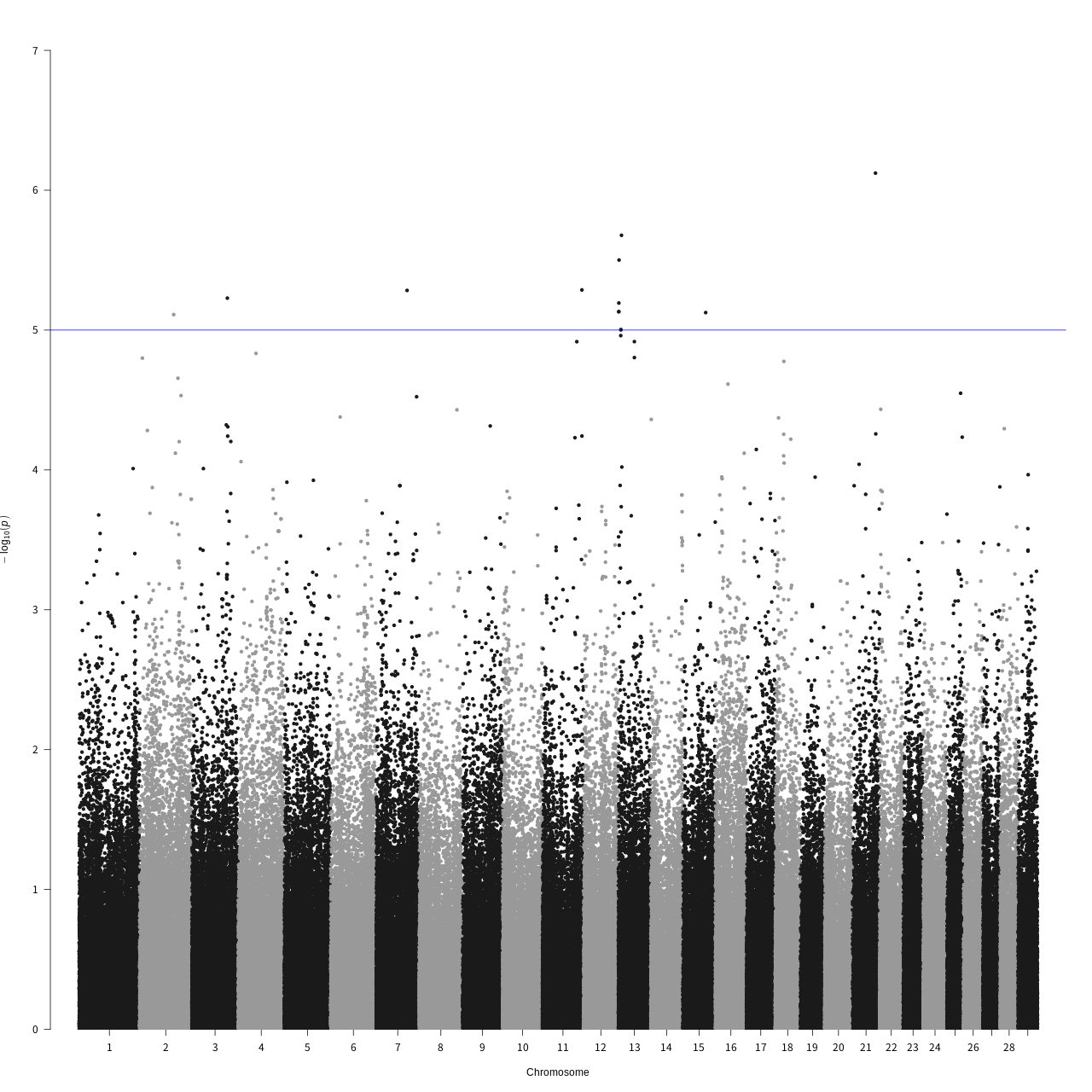

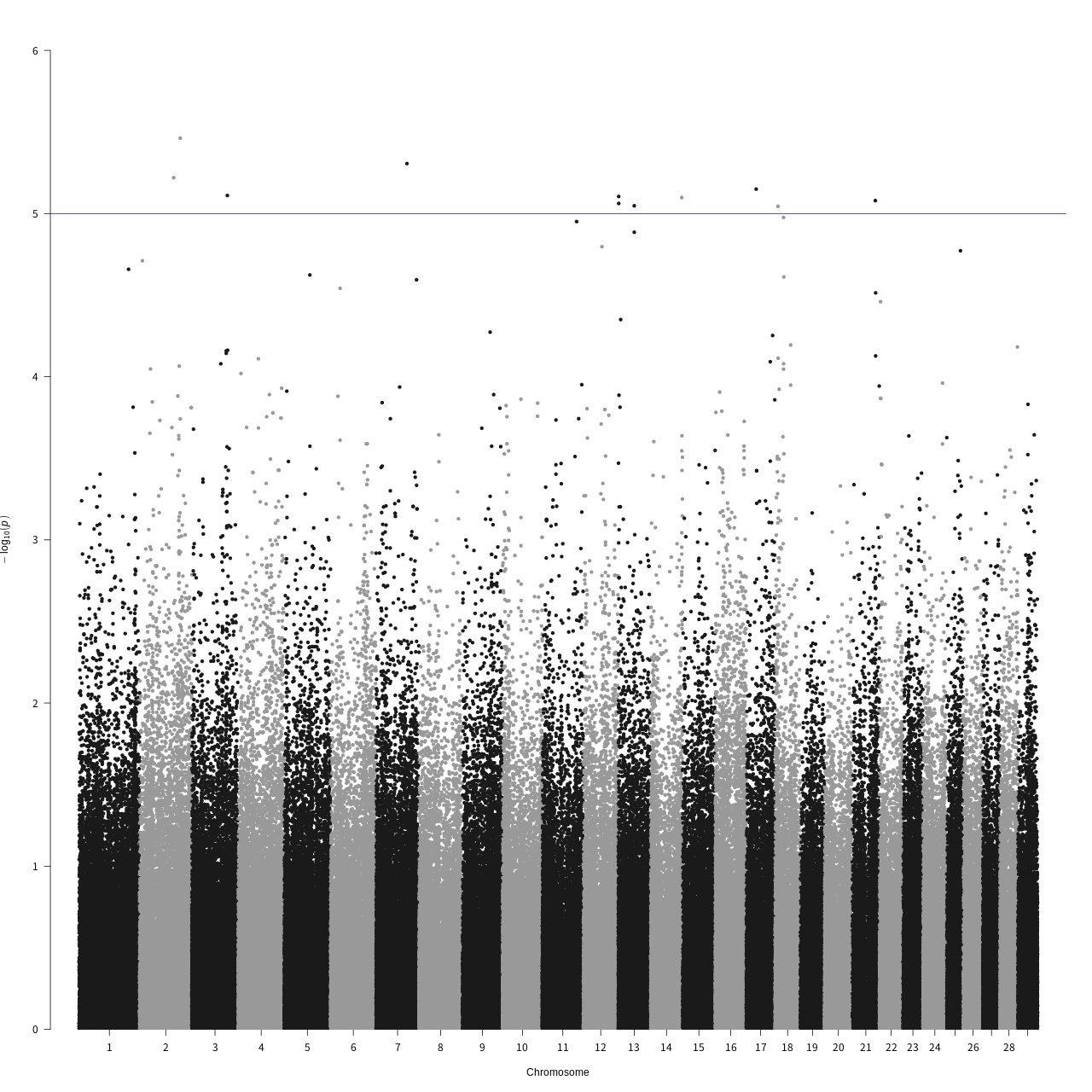

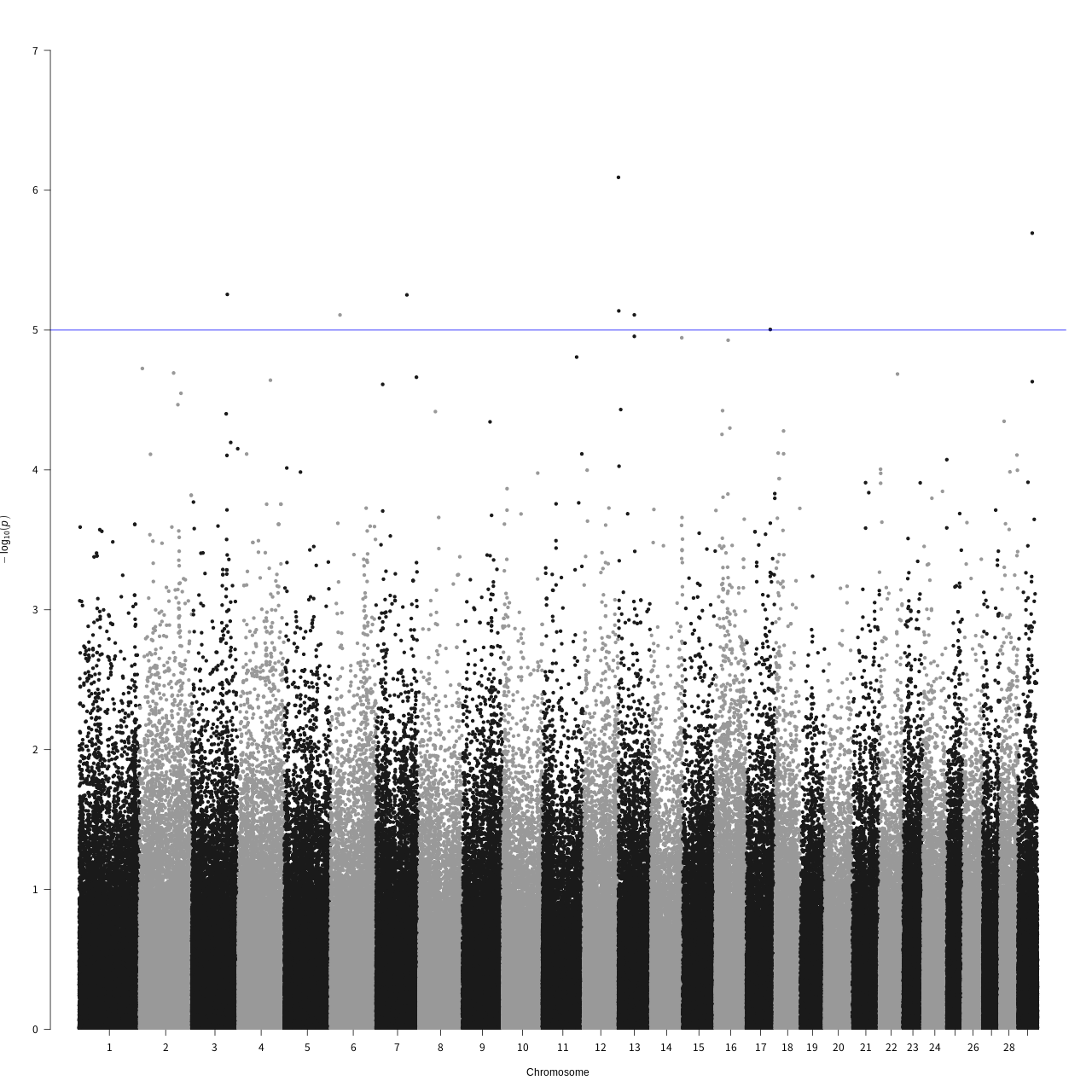


bft_ld_0.2_gemma bft_ld_0.3_gemma bft_ld_0.4_gemma


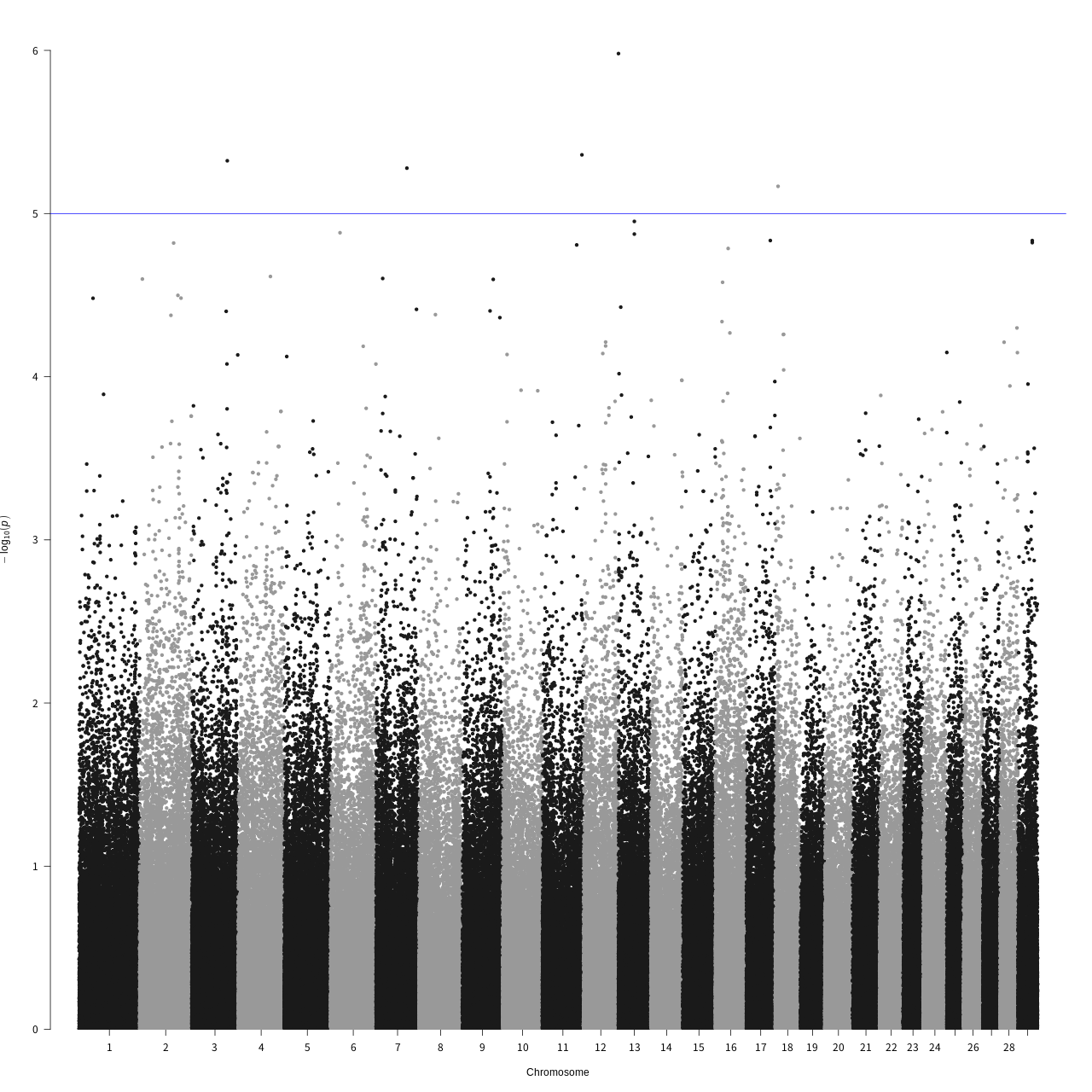

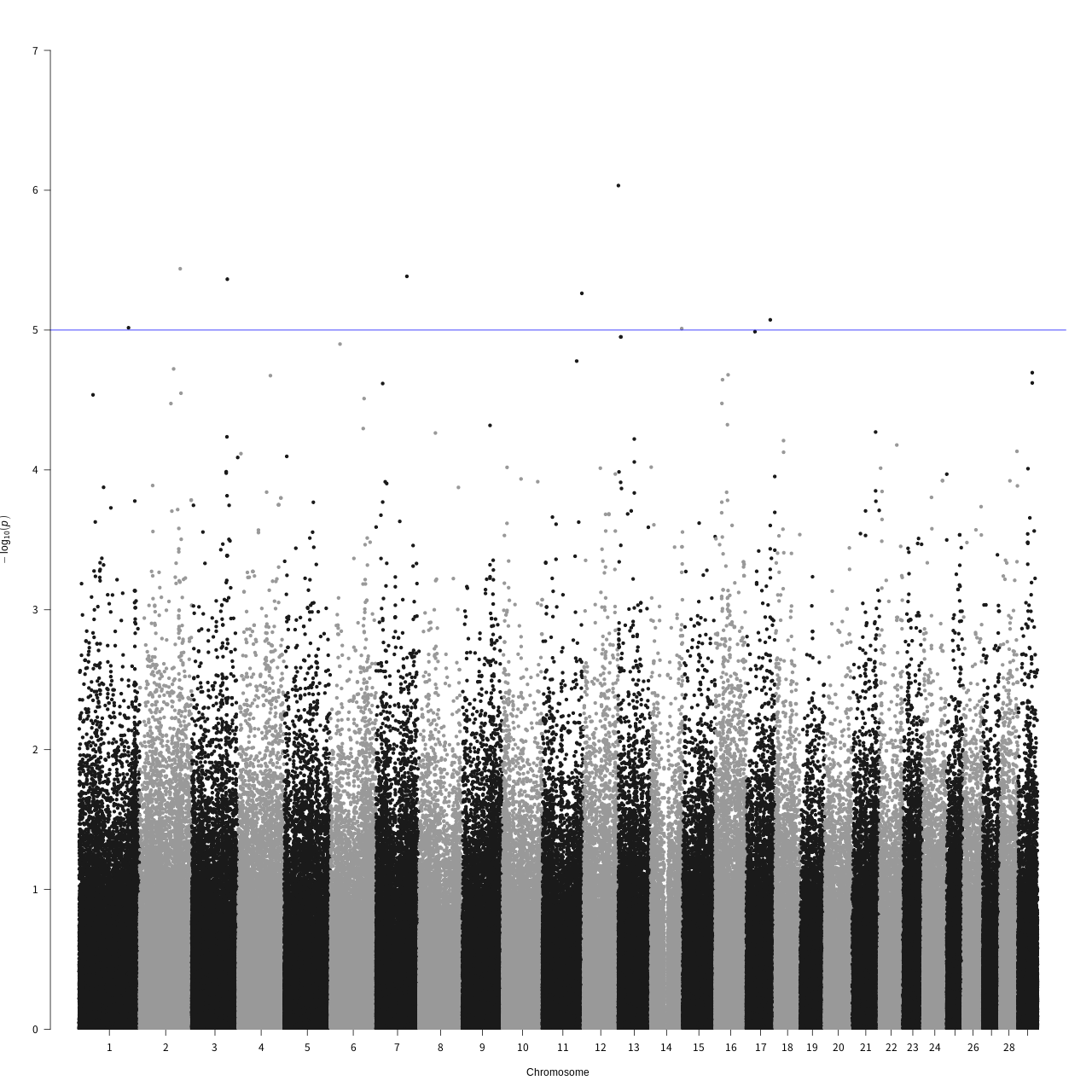

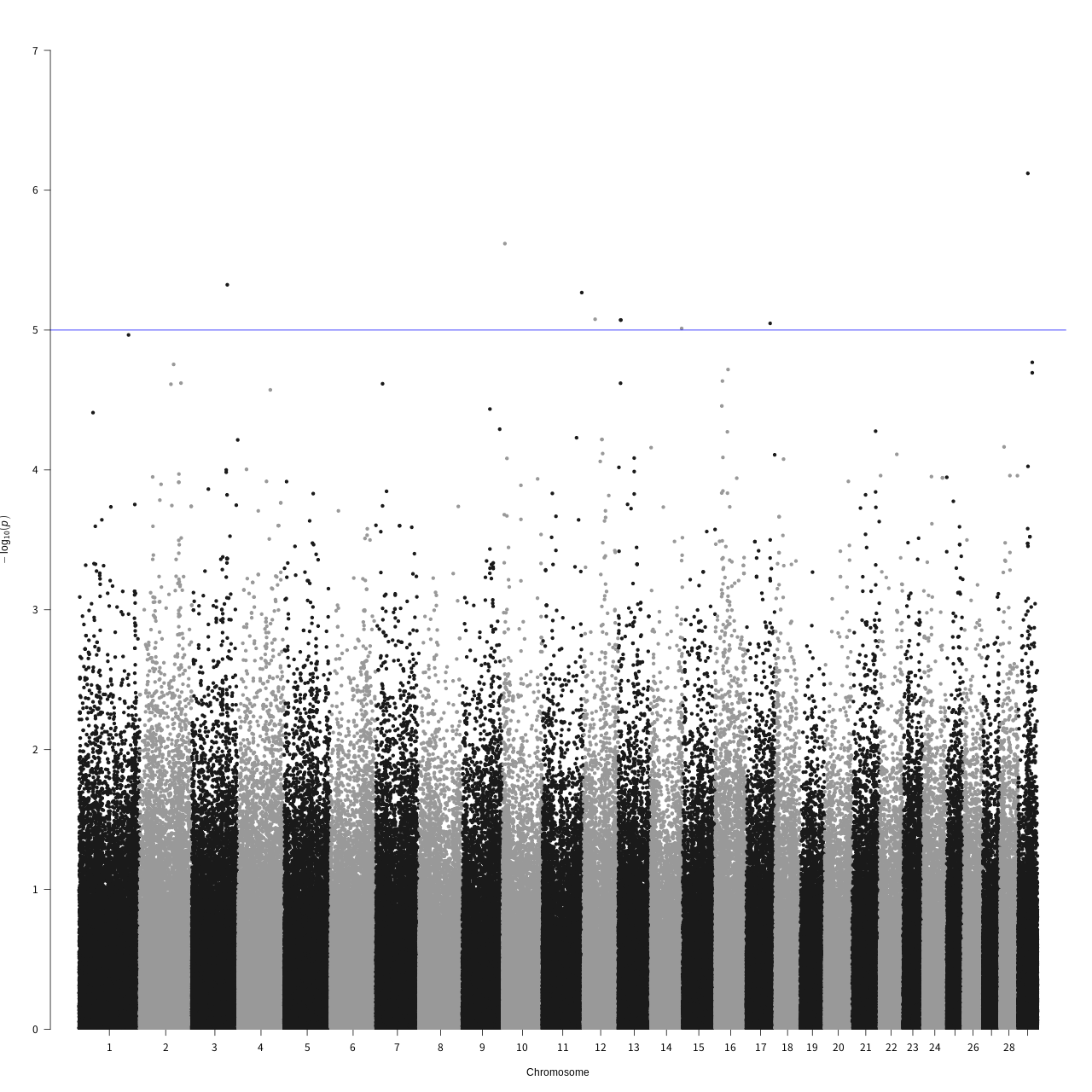


bft_ld_0.5_gemma bft_ld_0.6_gemma bft_ld_0.7_gemma


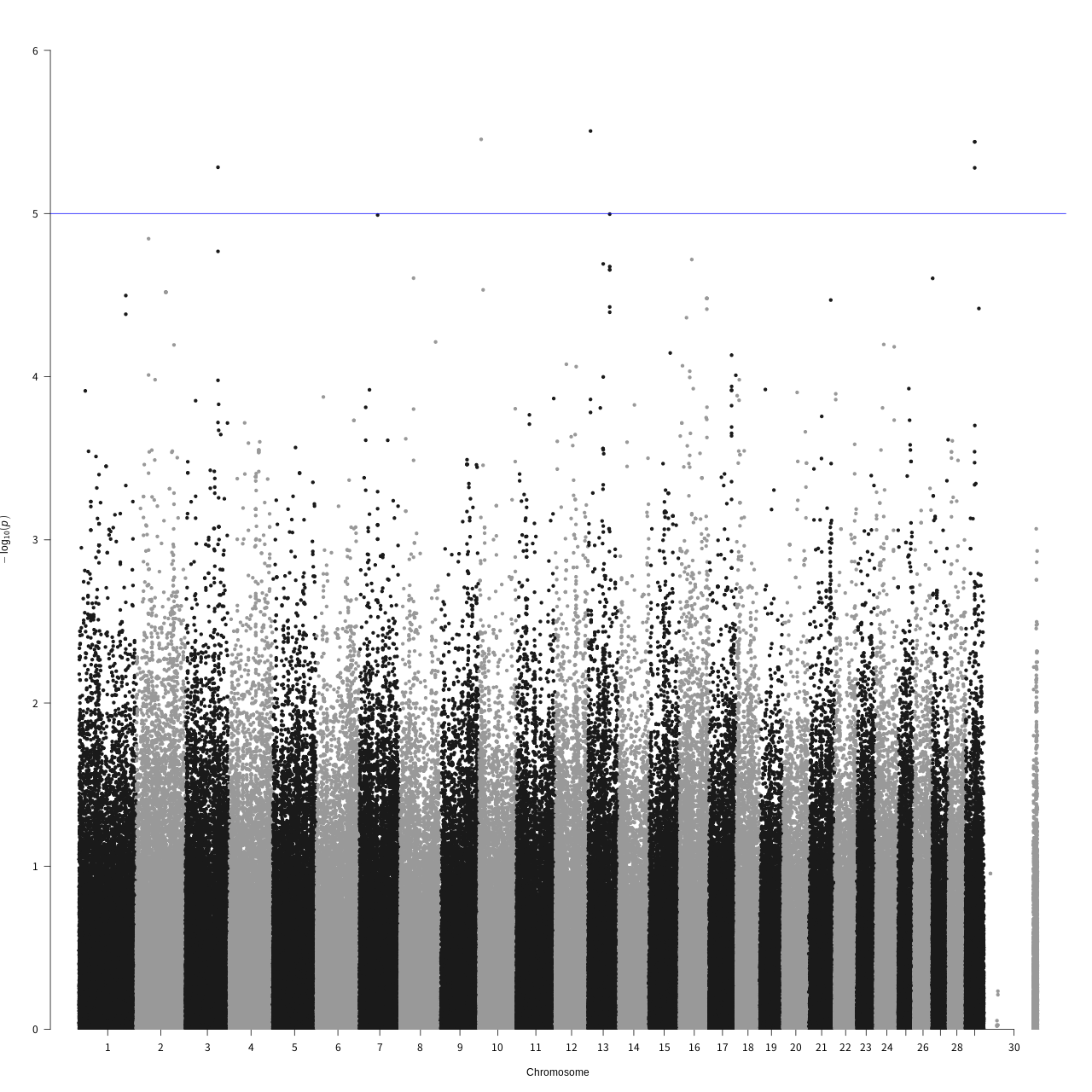

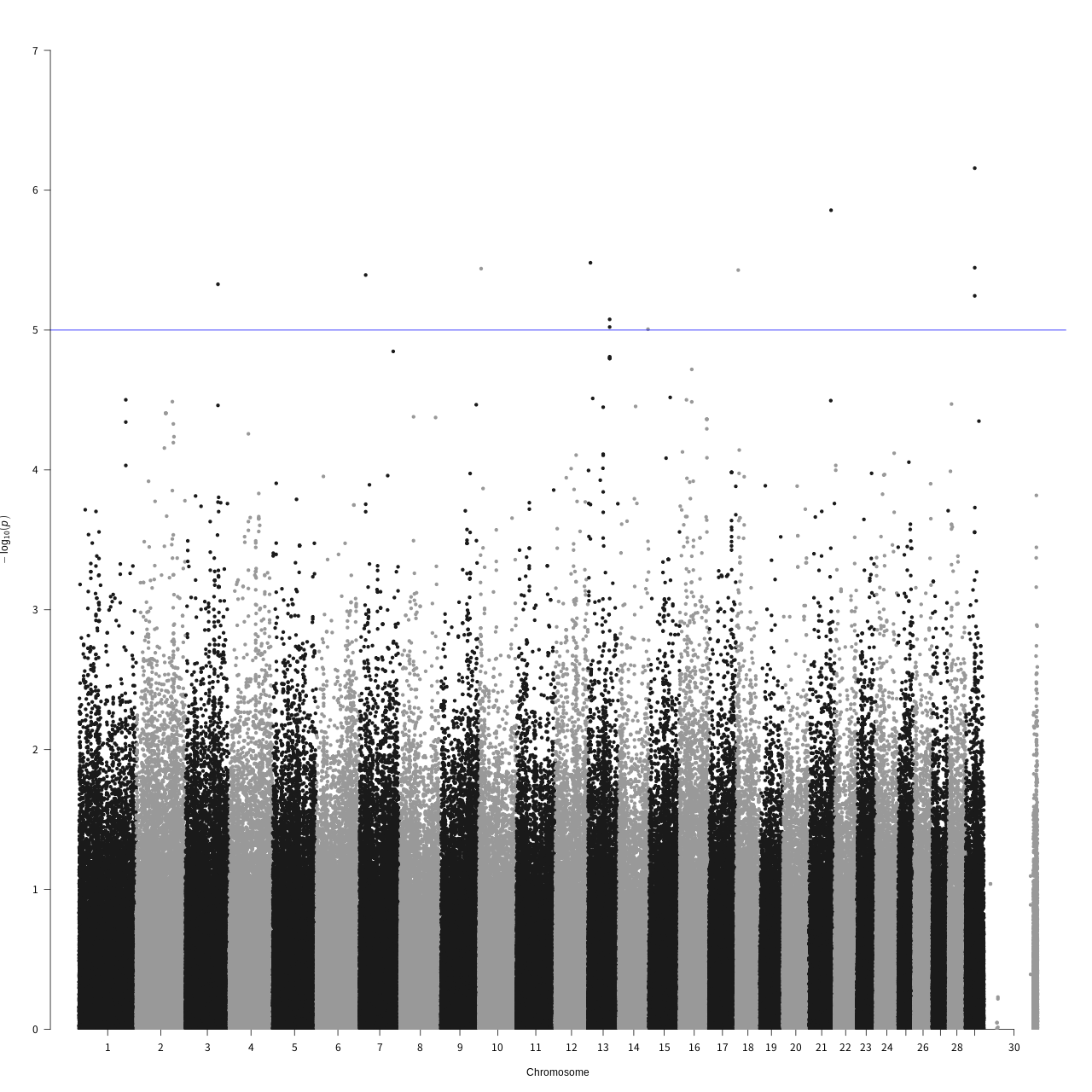

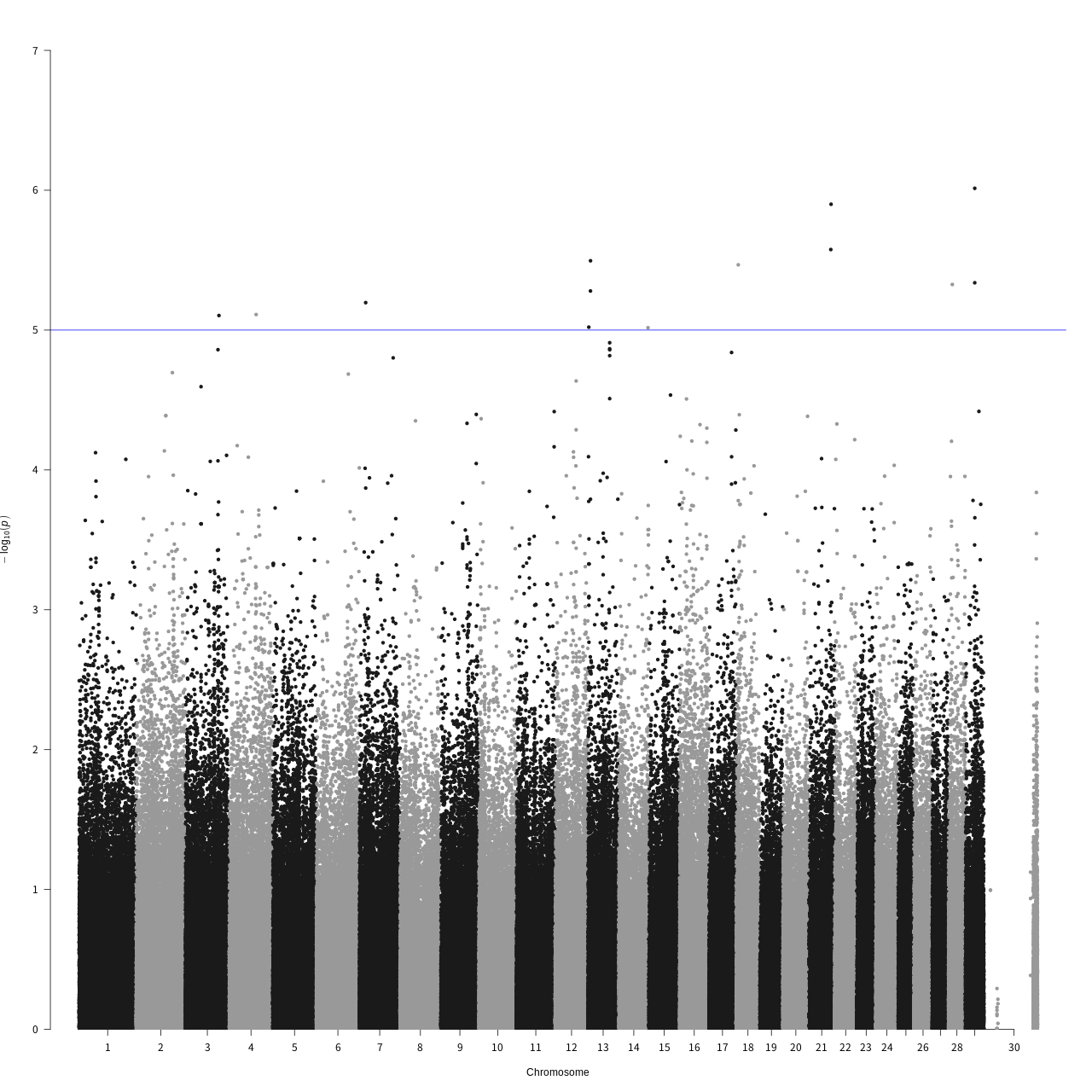


bft_len_5_gemma bft_len_10_gemma bft_len_20_gemma


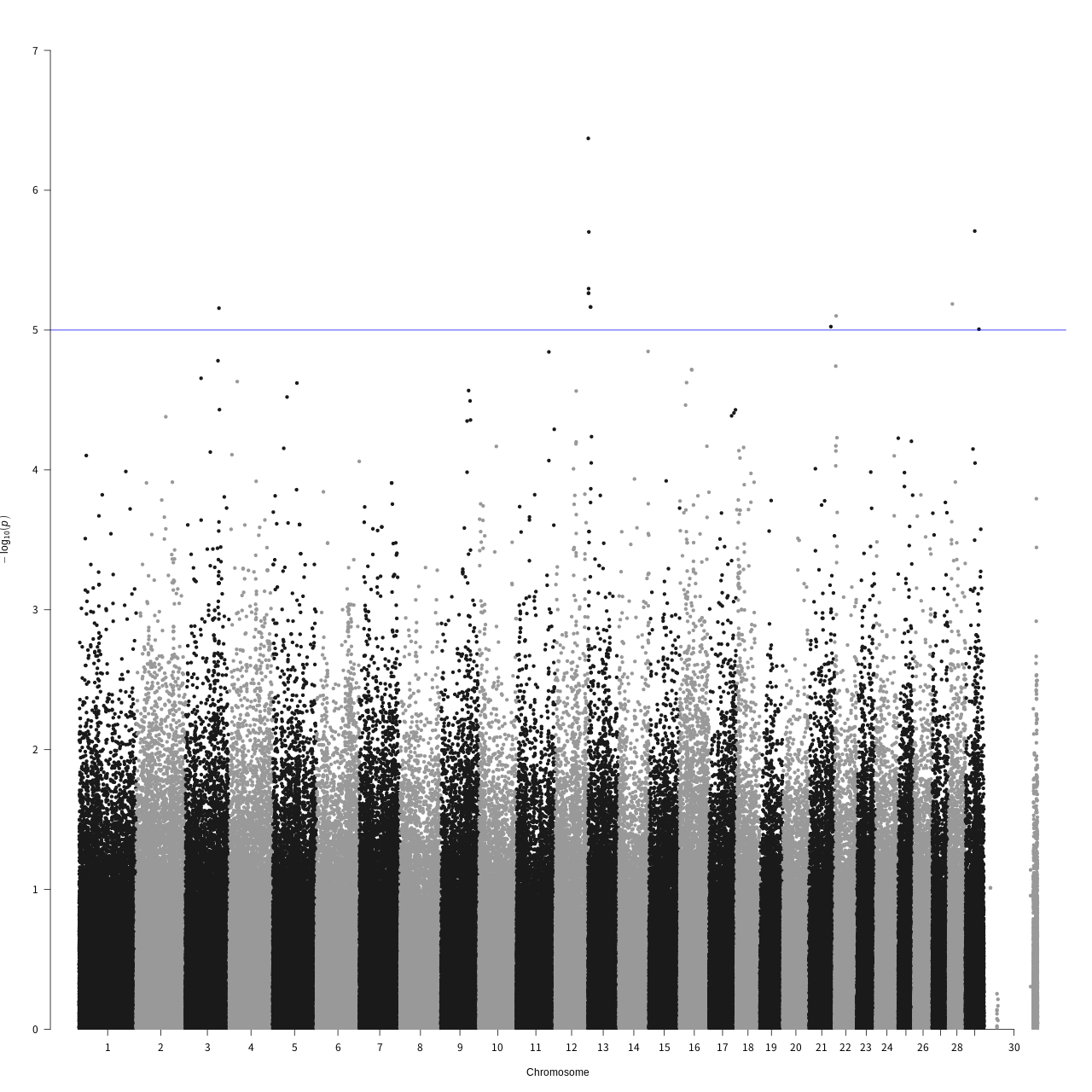

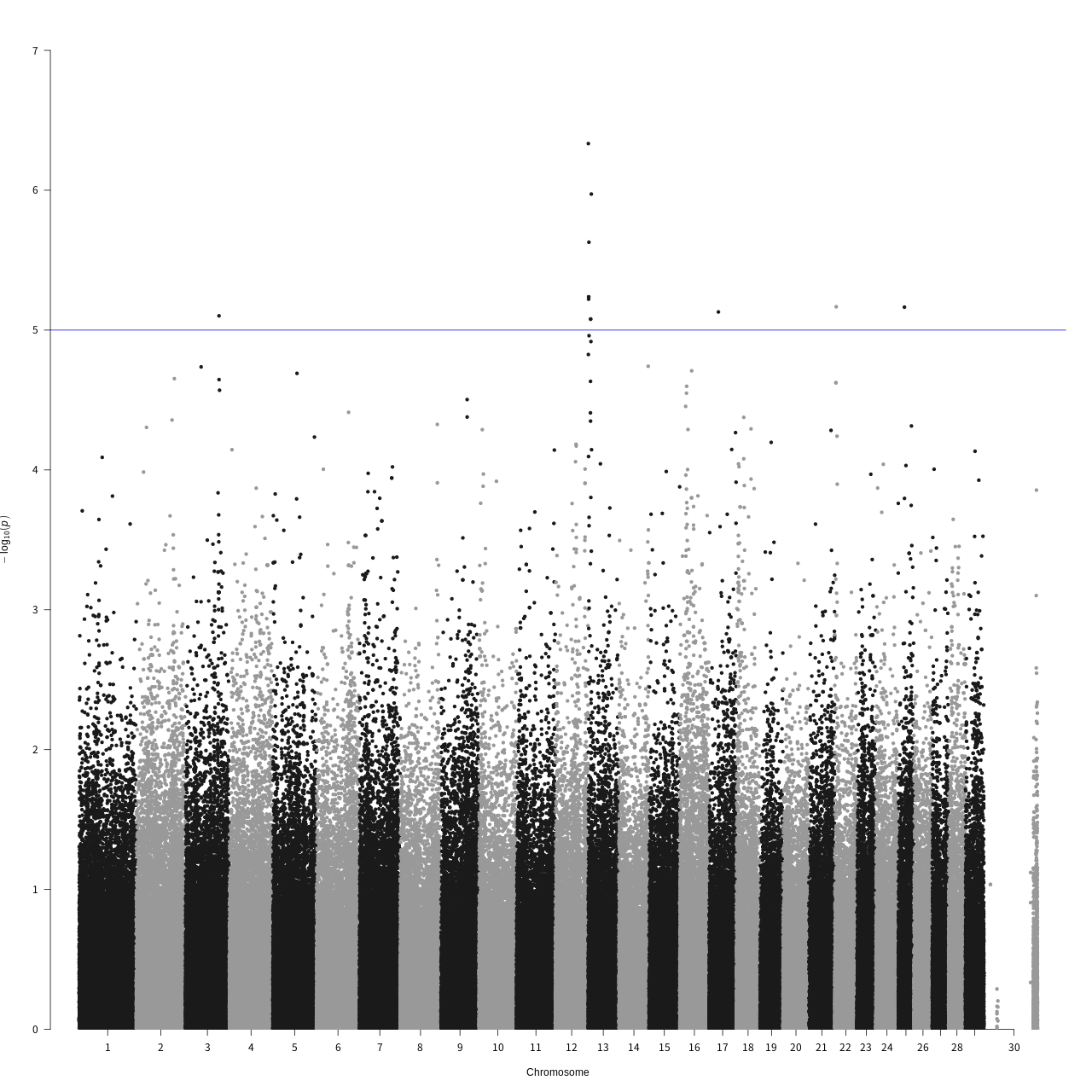

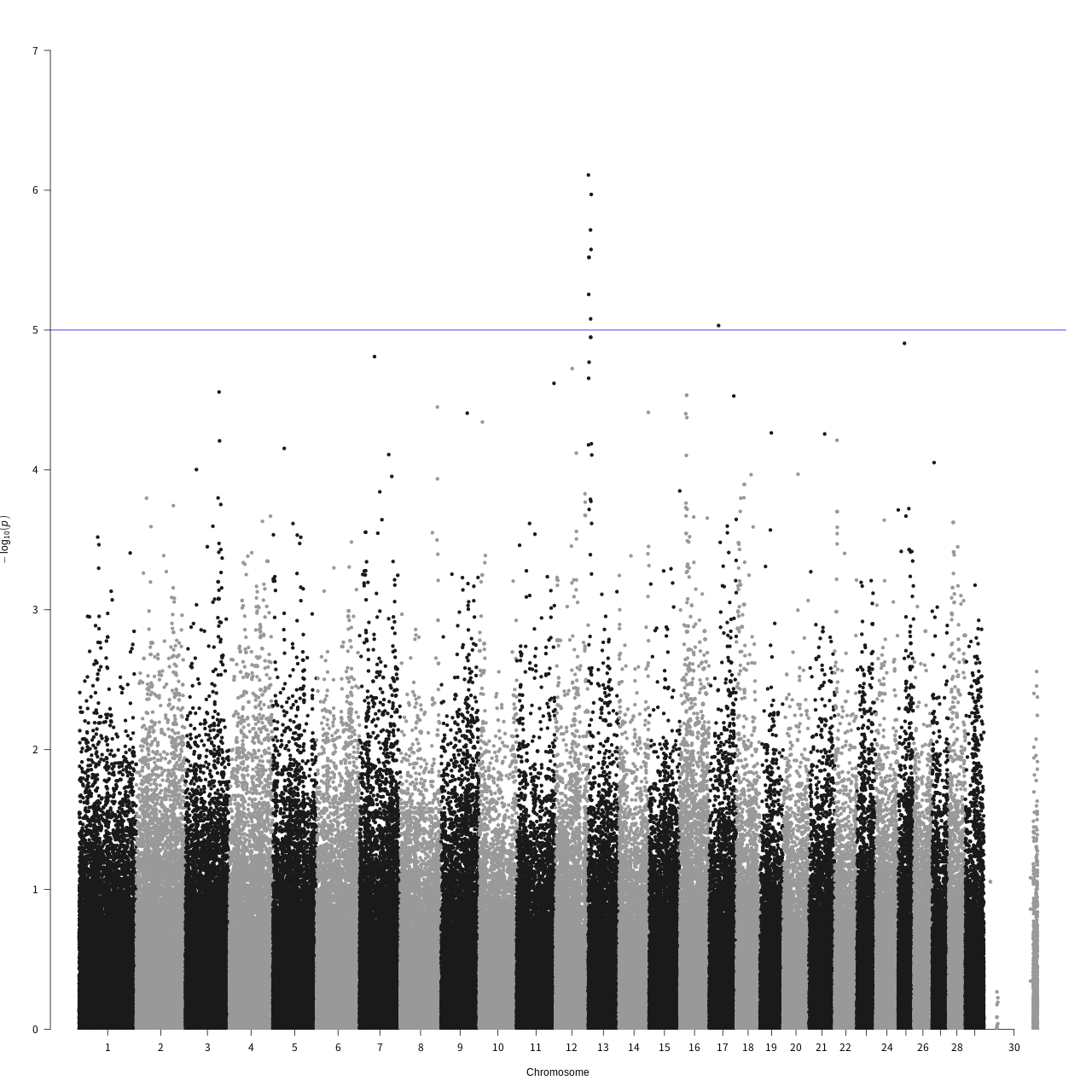


bft_len_50_gemma bft_len_100_gemma bft_len_200_gemma


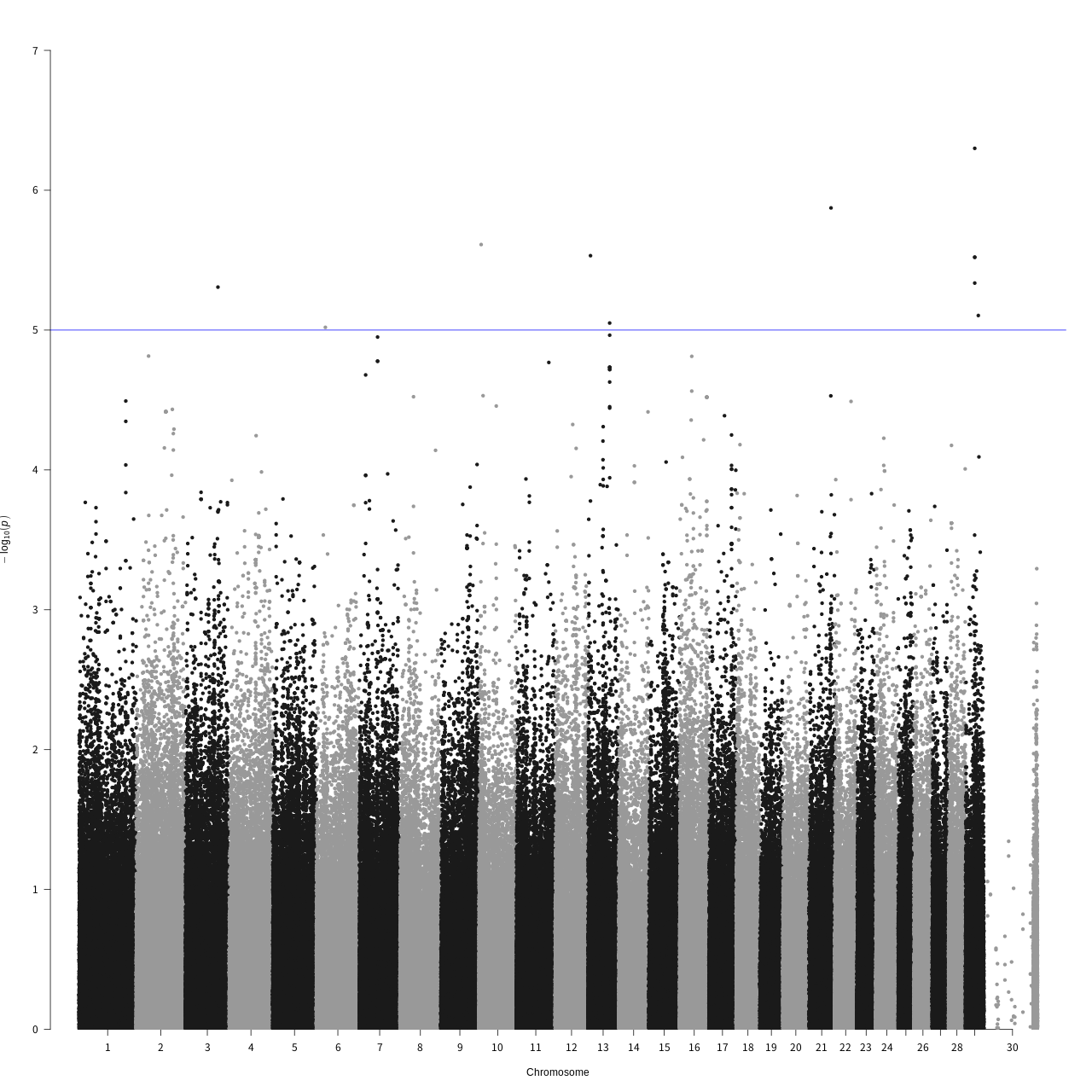

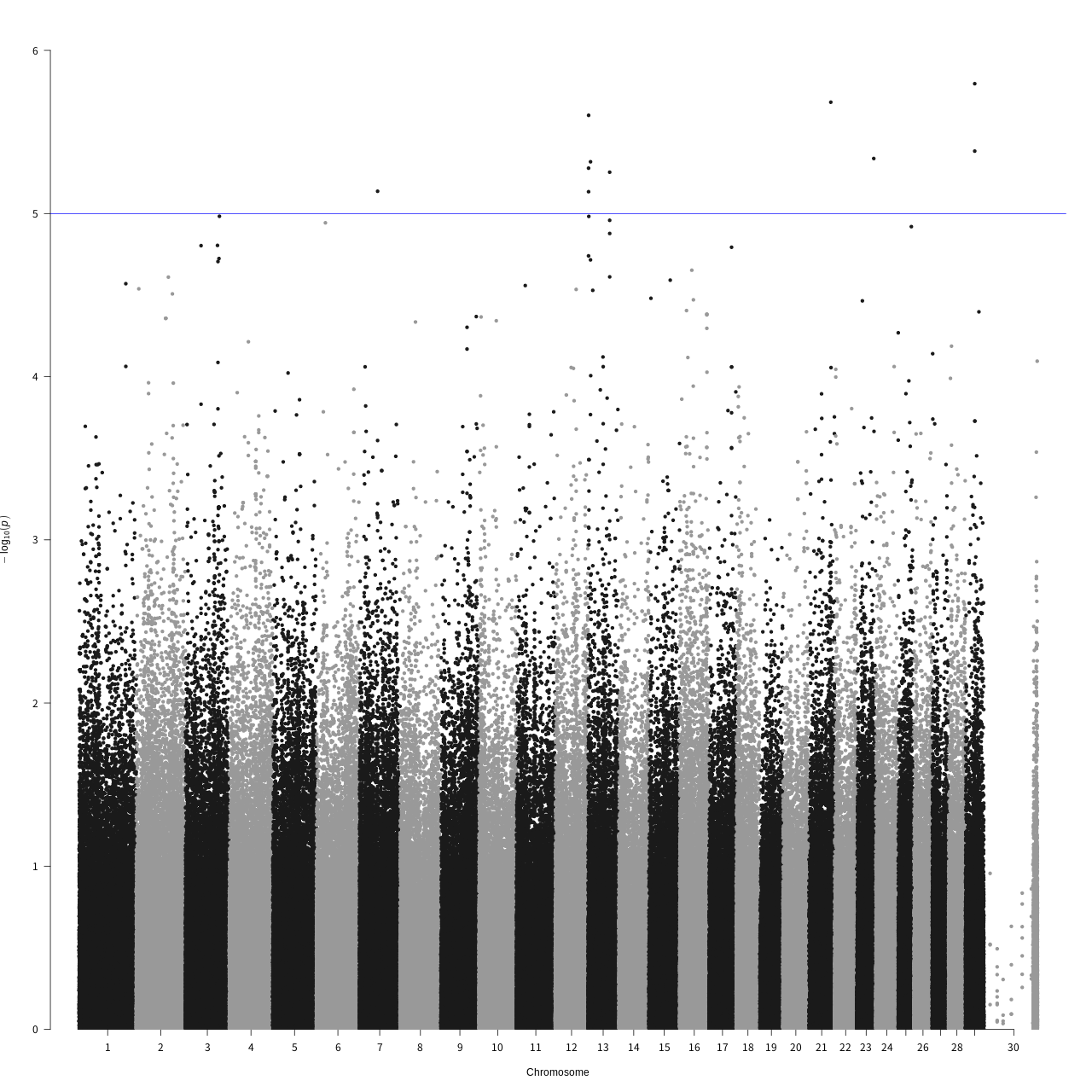

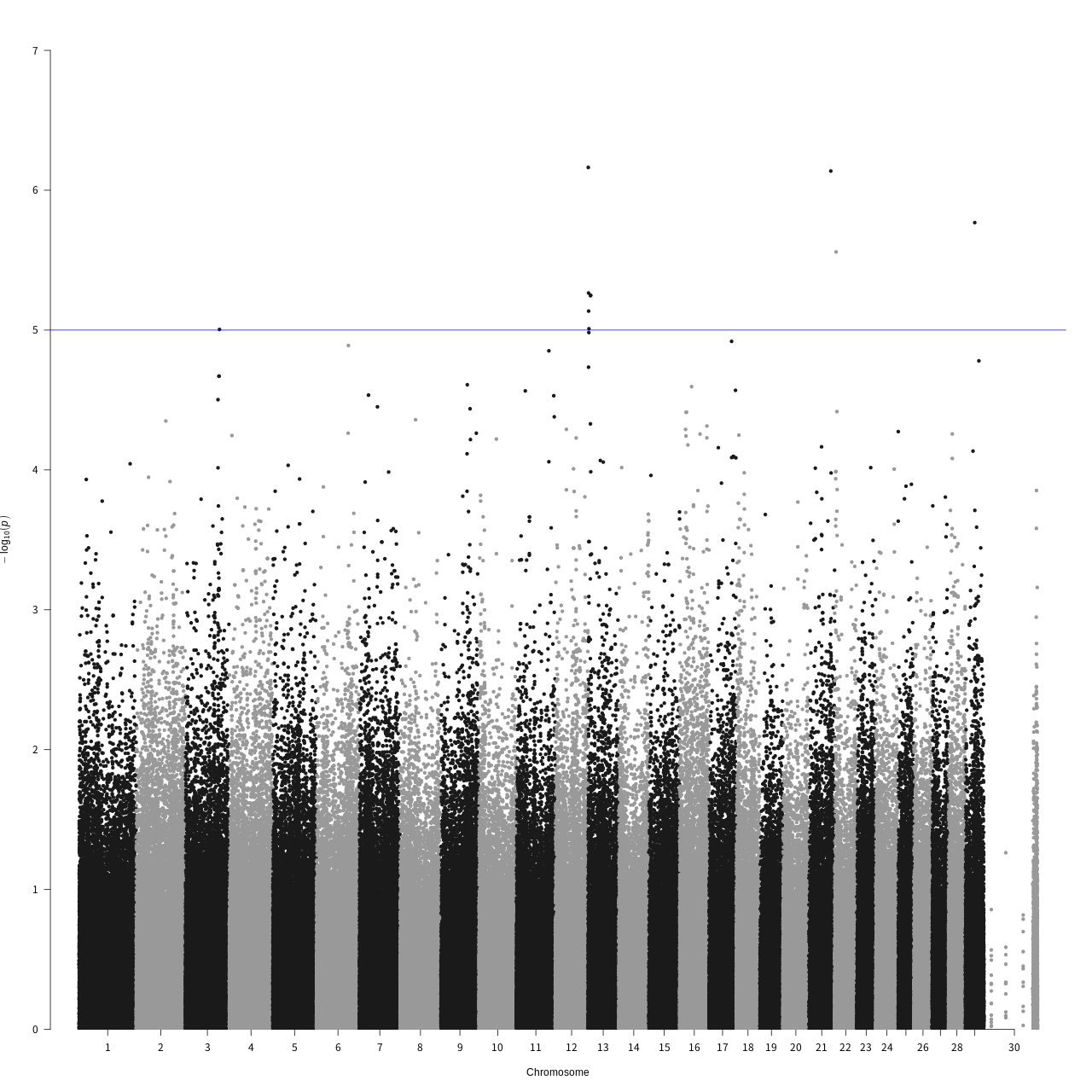


bft_nsnp_2_gemma bft_nsnp_5_gemma bft_nsnp_10_gemma


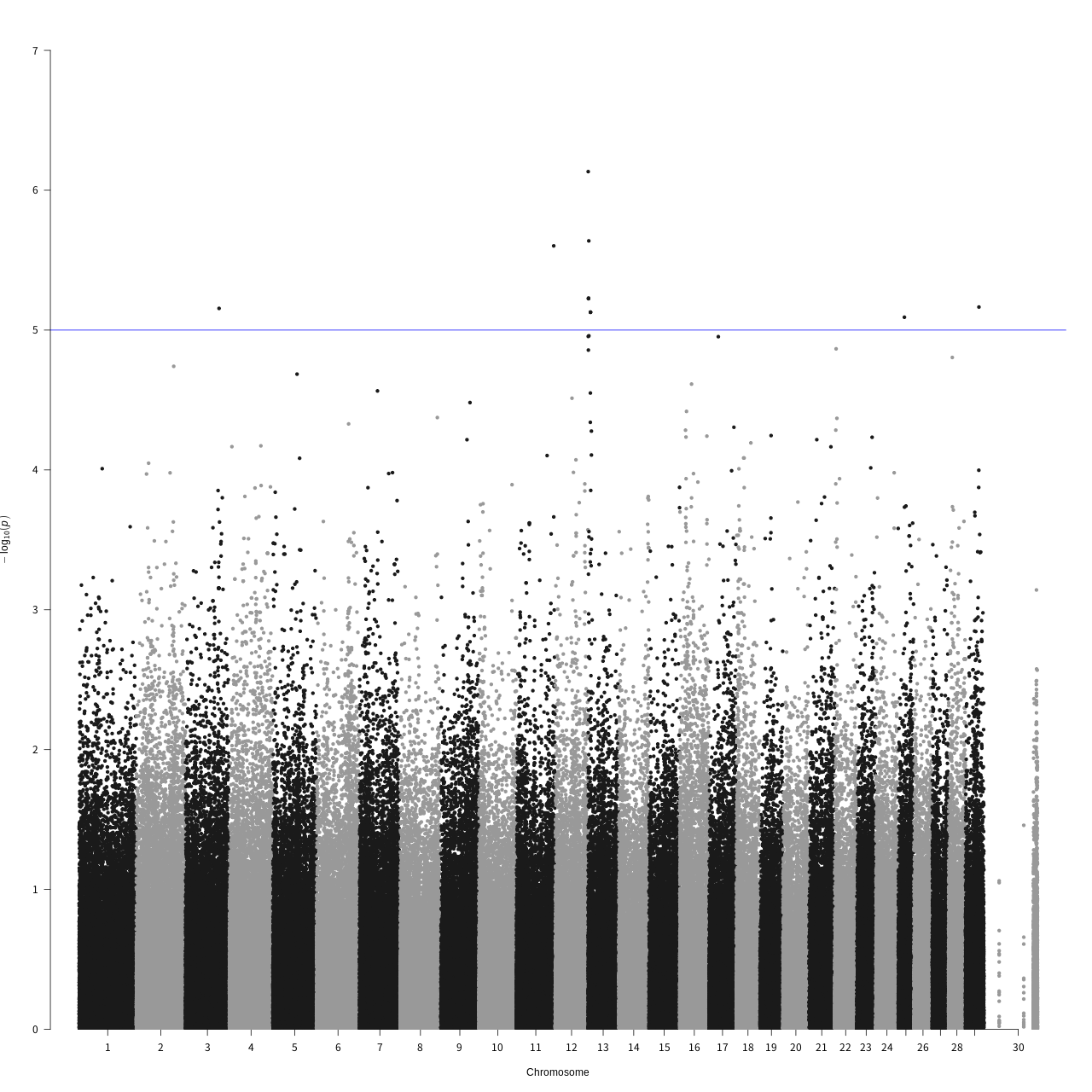

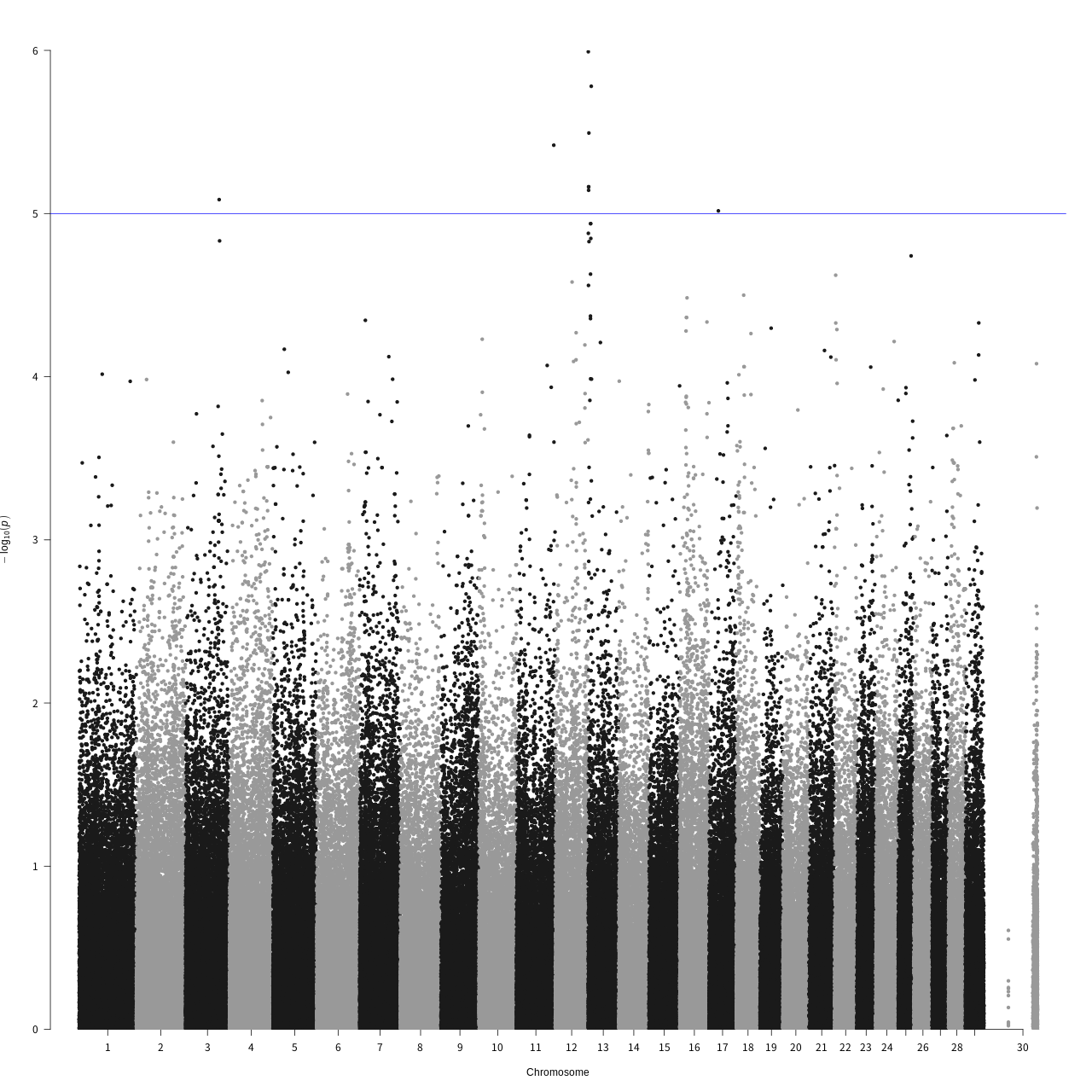

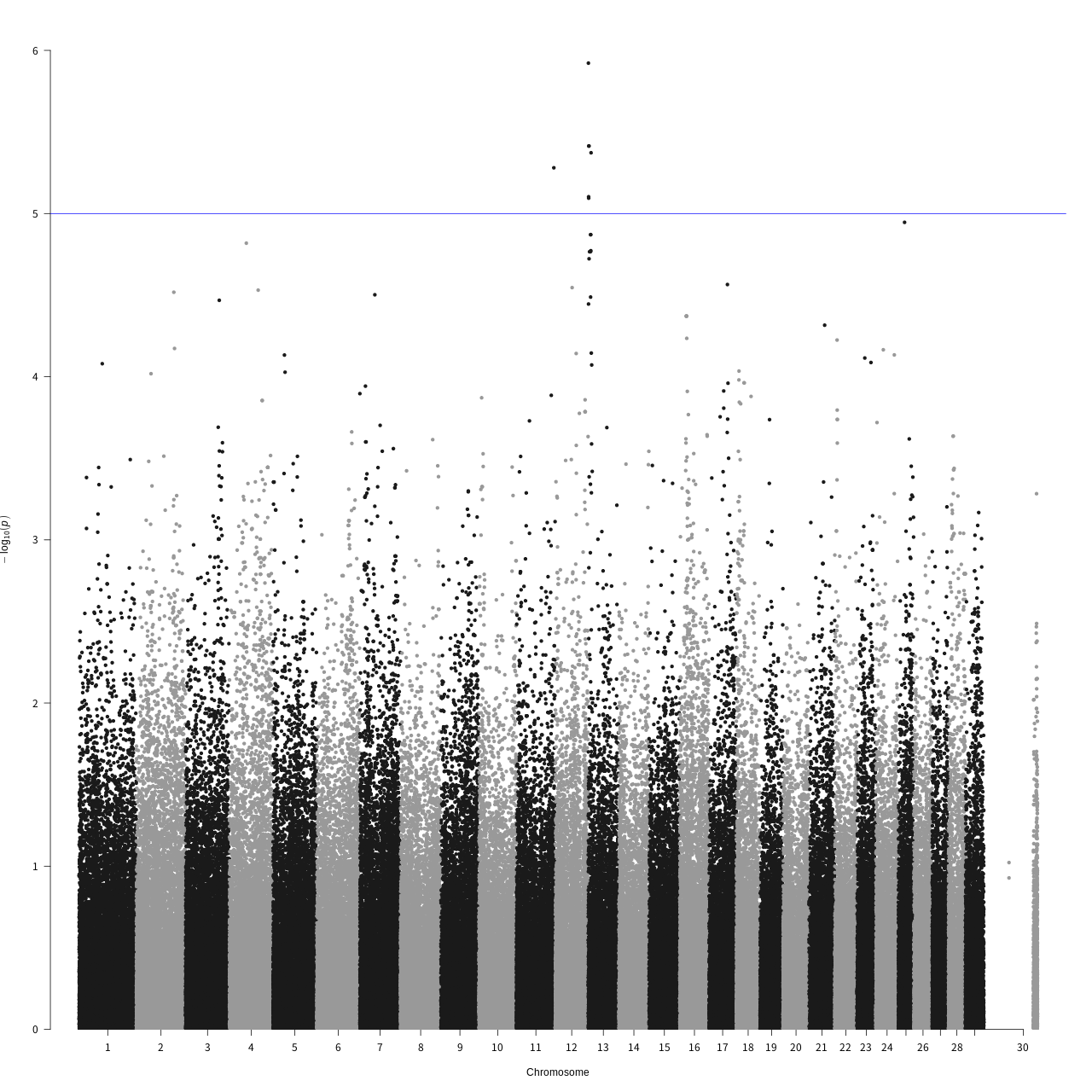


bft_nsnp_20_gemma bft_nsnp_30_gemma bft_nsnp_50_gemma

1. Manhattan for CWT


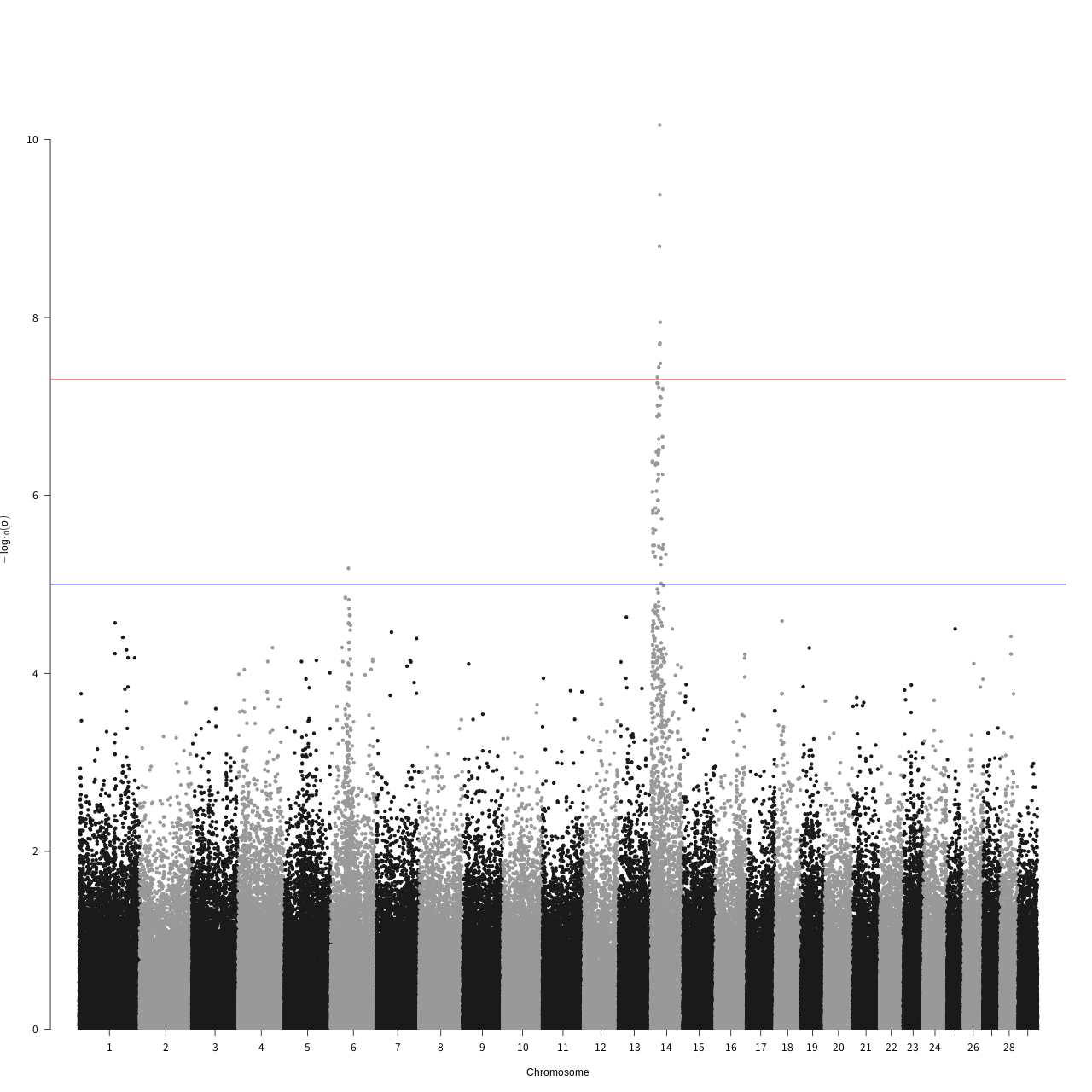

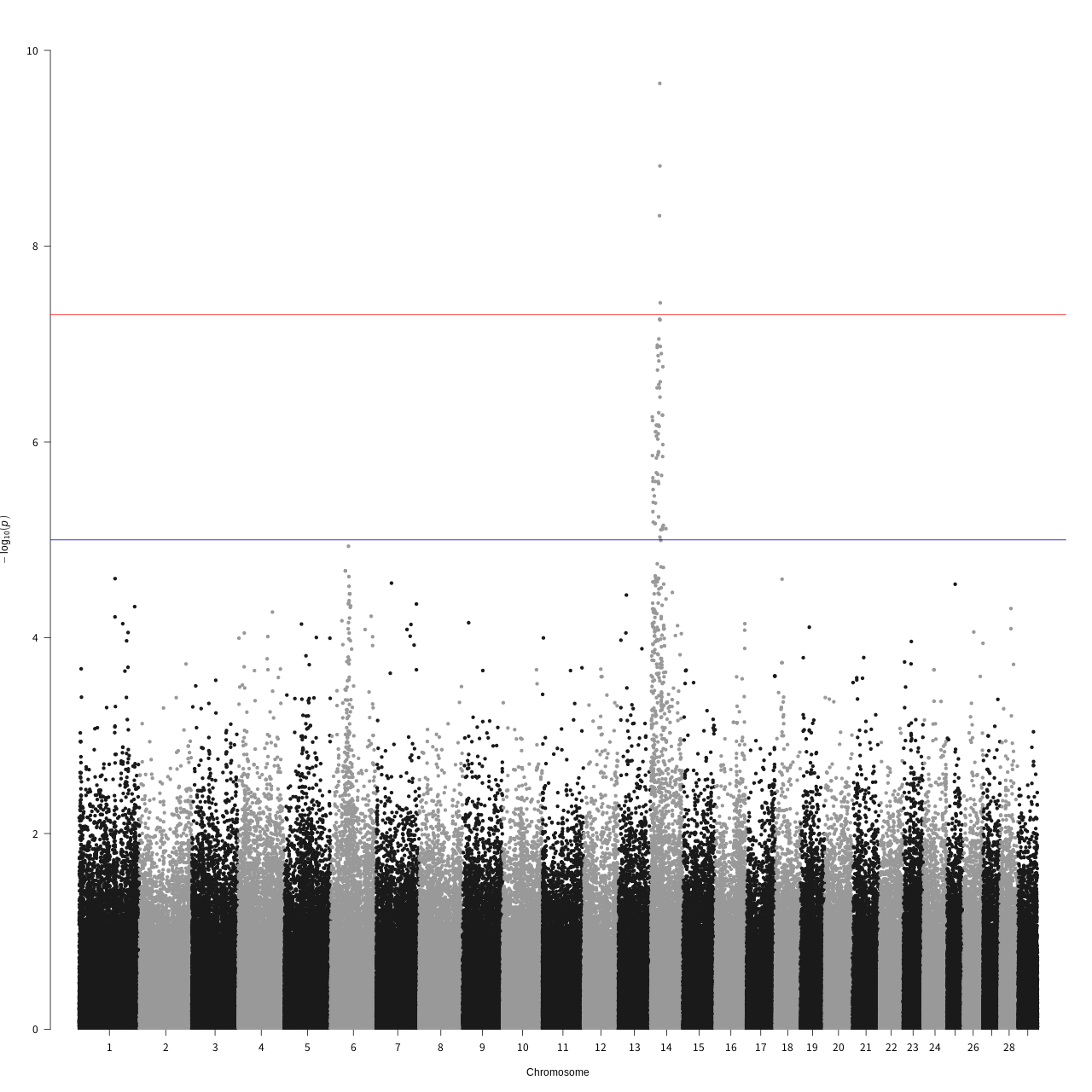

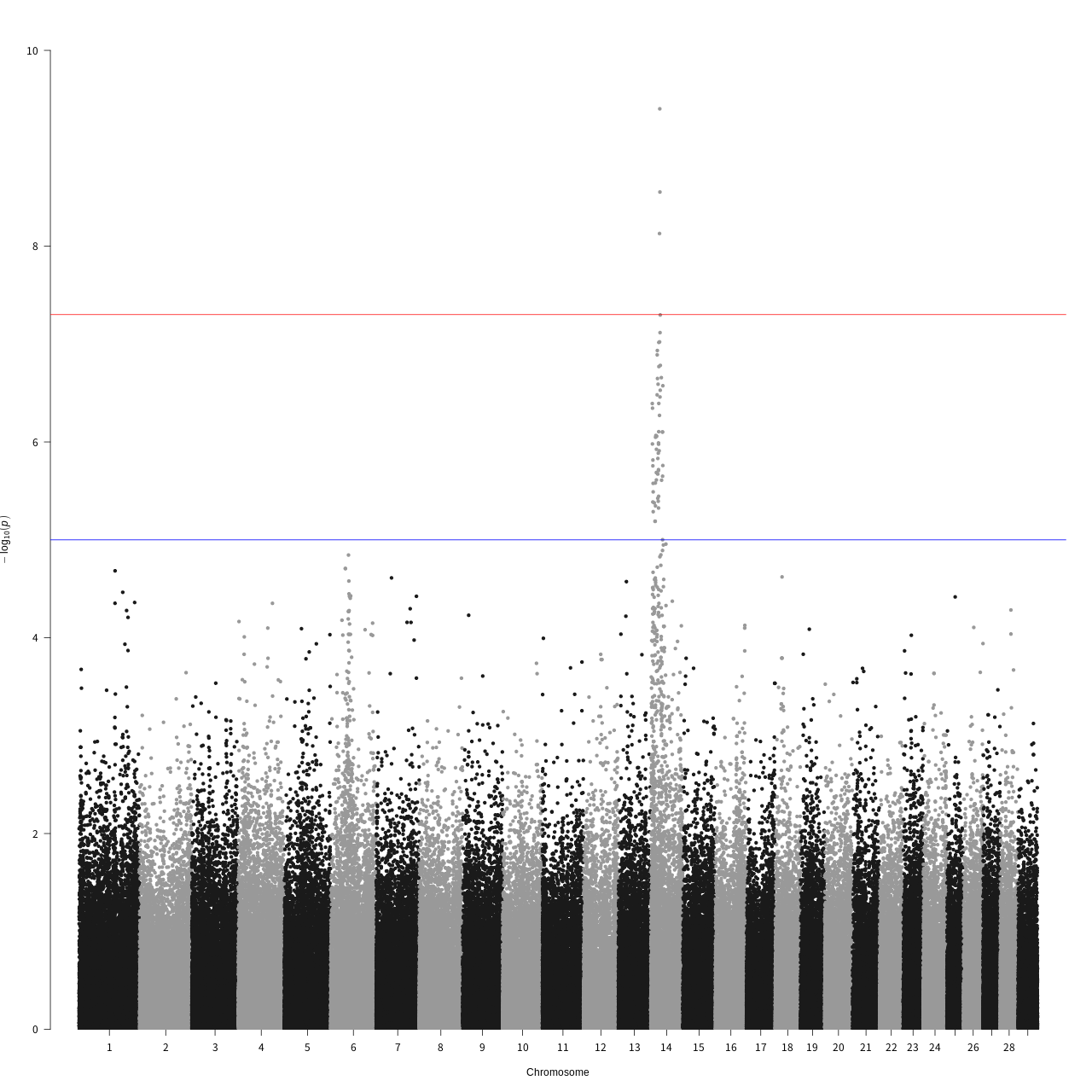


cwt_ld_0.2_gemma cwt_ld_0.3_gemma cwt_ld_0.4_gemma


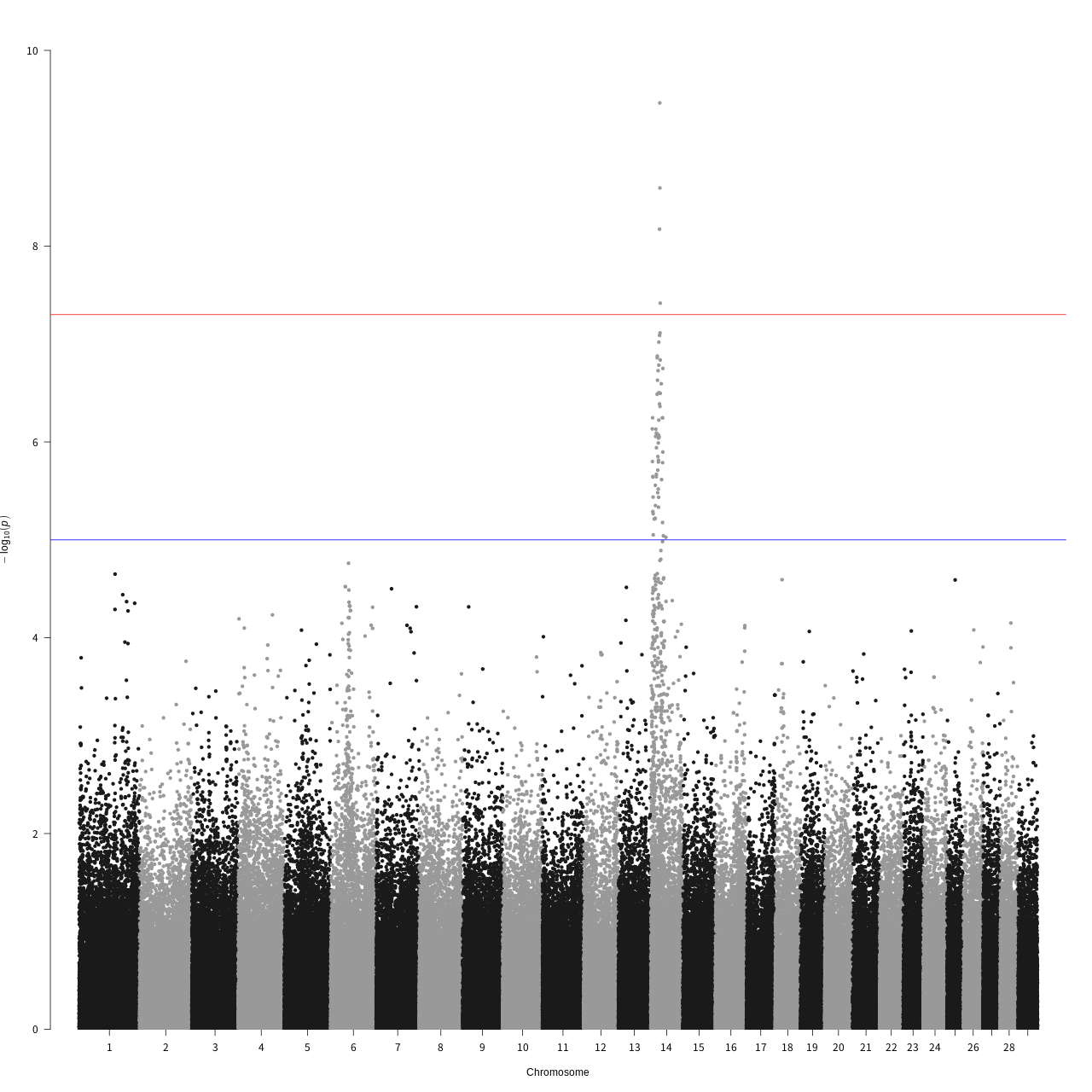

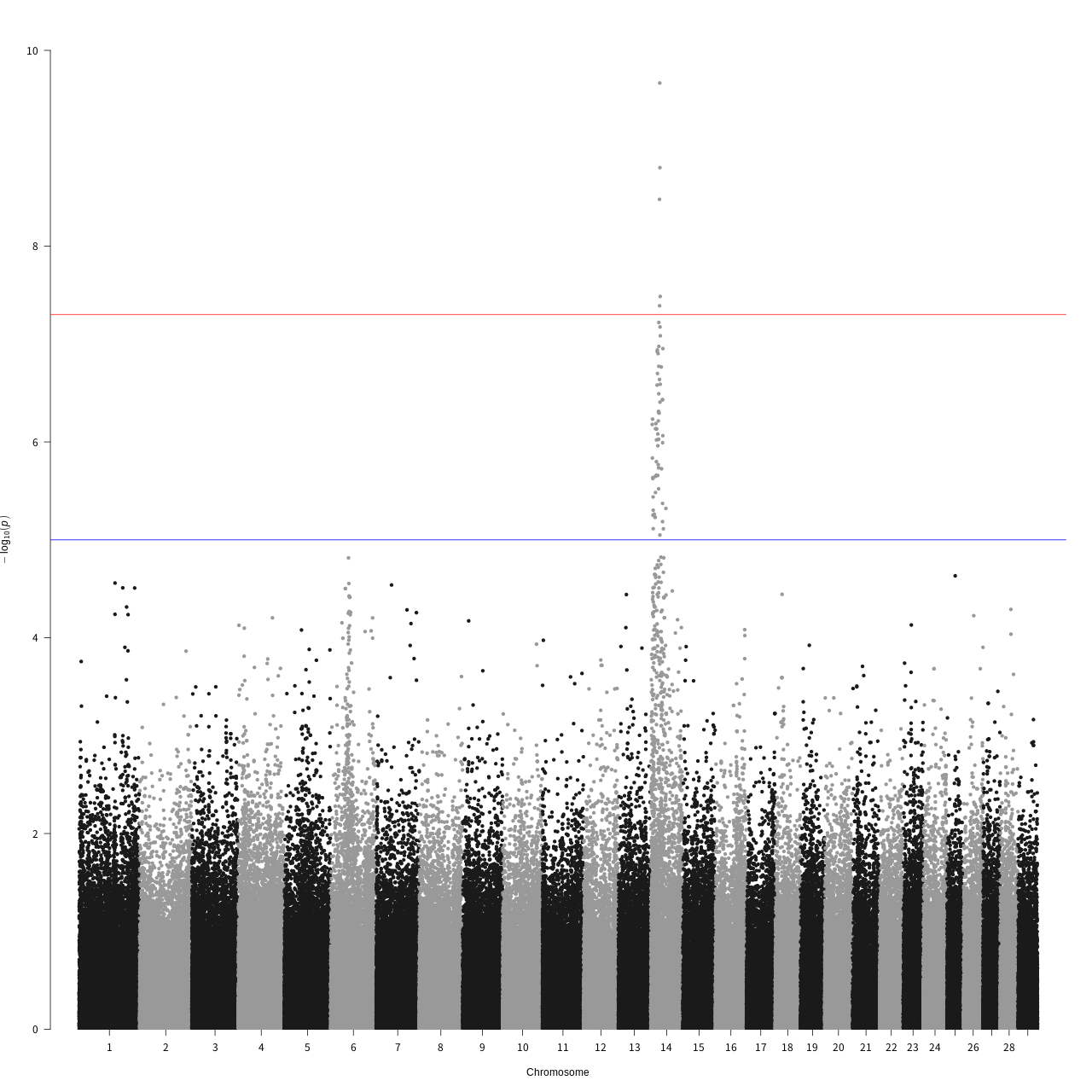

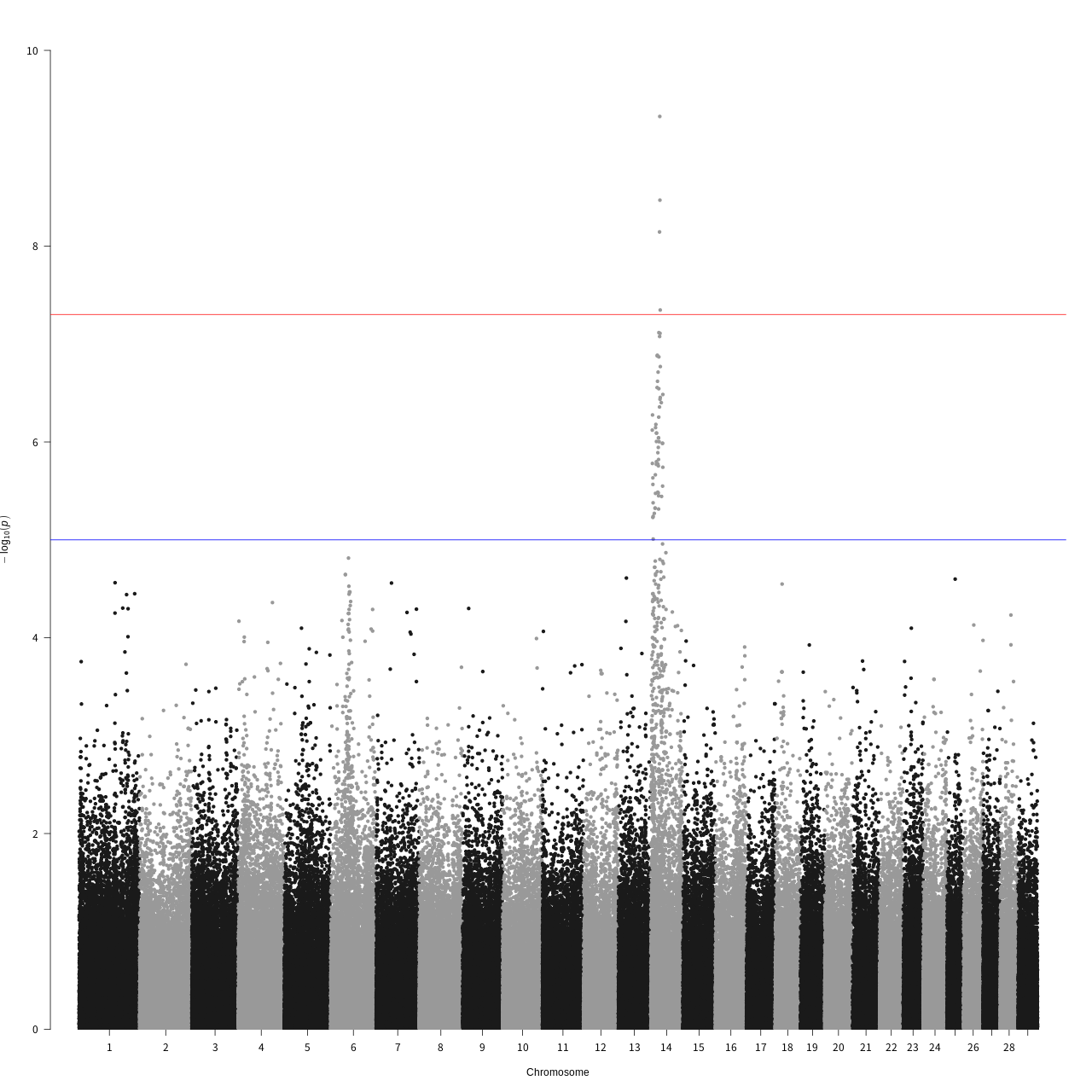


cwt_ld_0.5_gemma cwt_ld_0.6_gemma cwt_ld_0.7_gemma


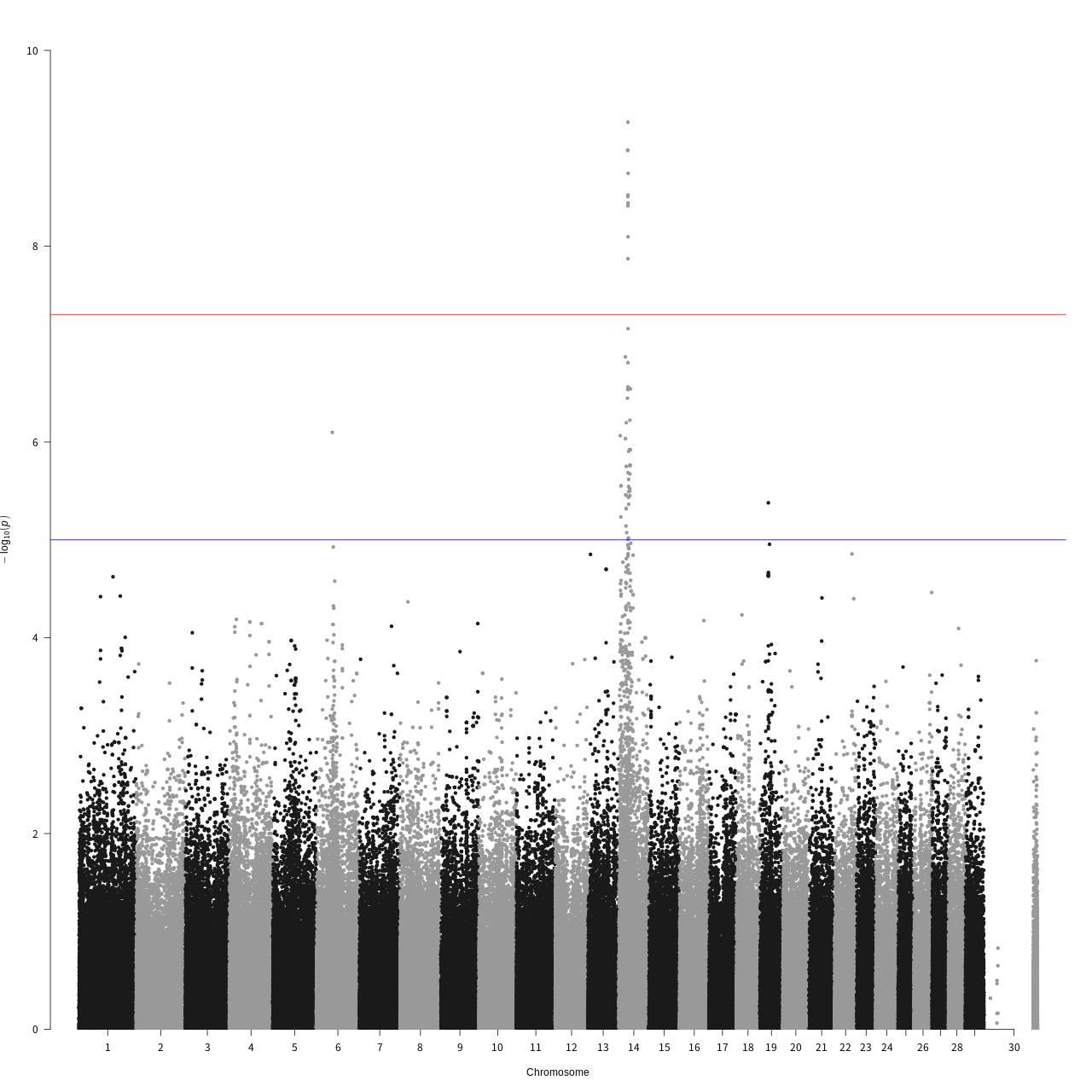

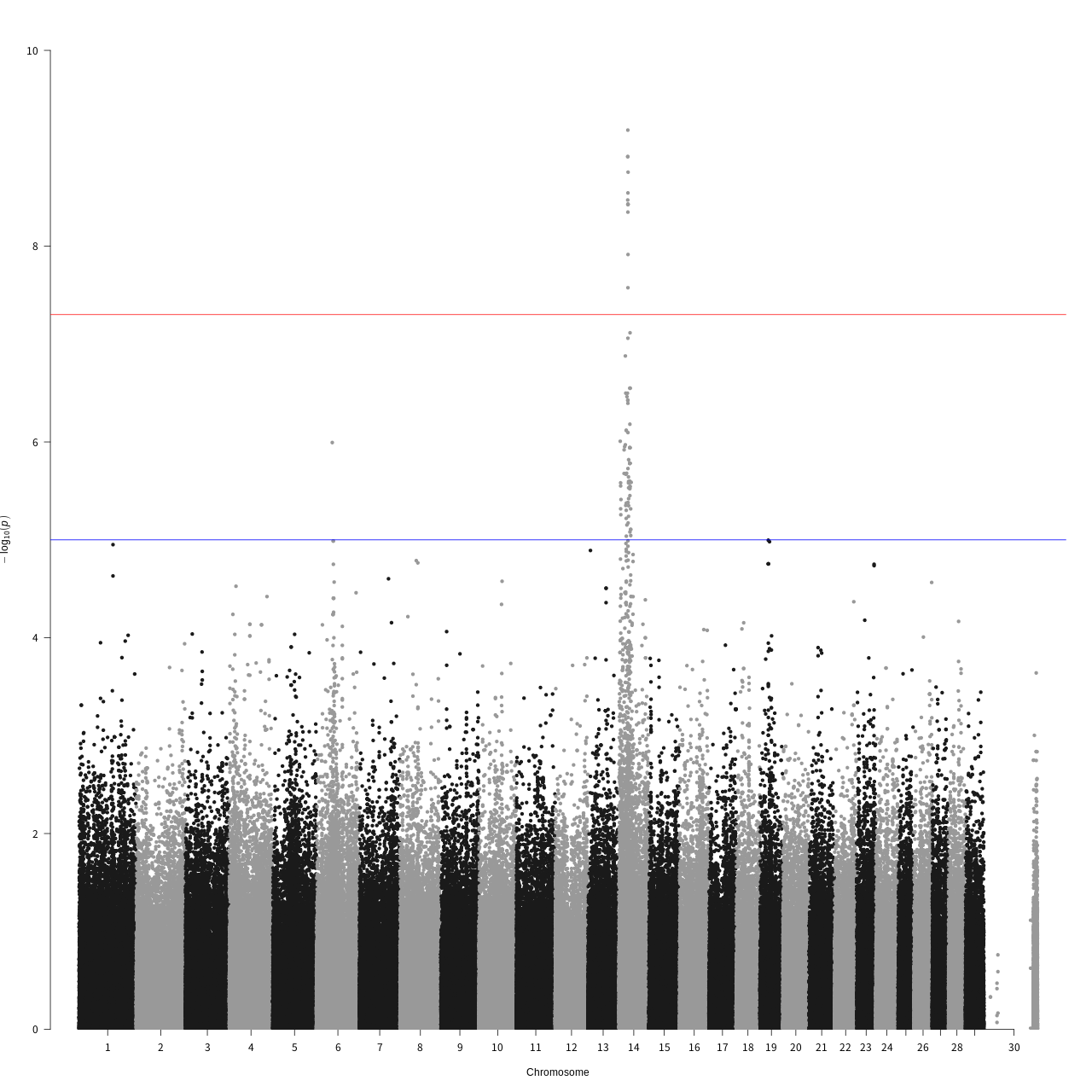

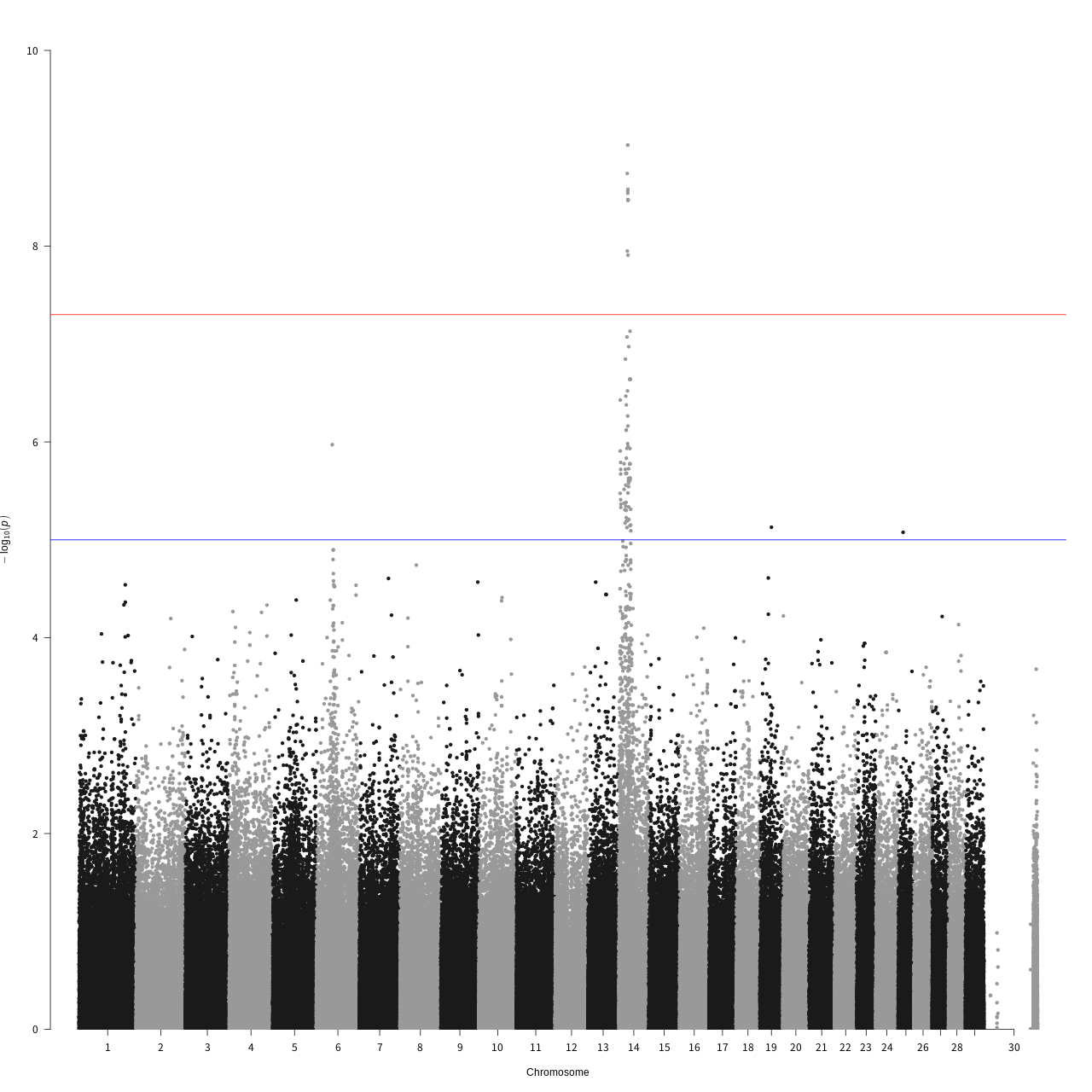


cwt_len_5_gemma cwt_len_10_gemma cwt_len_20_gemma


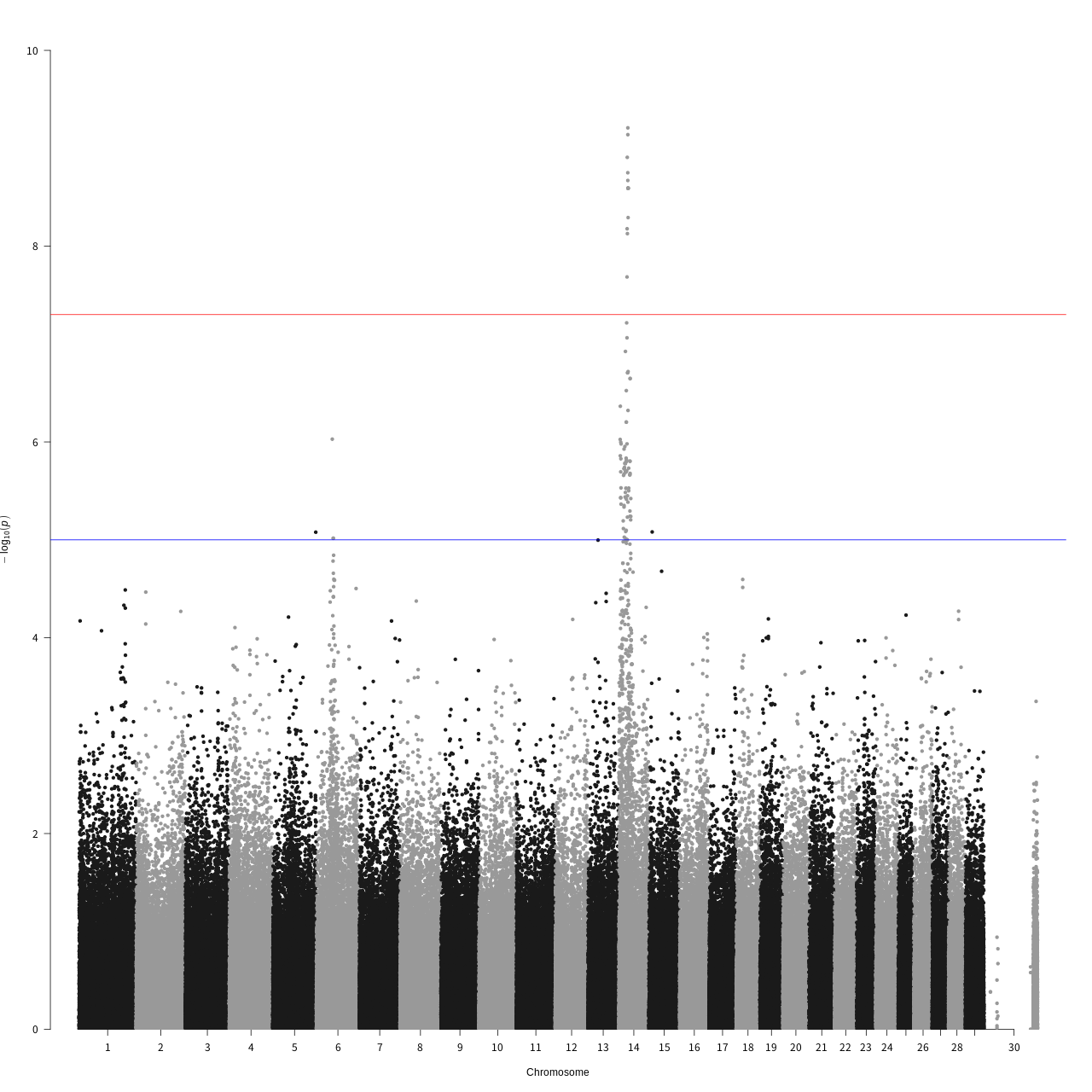

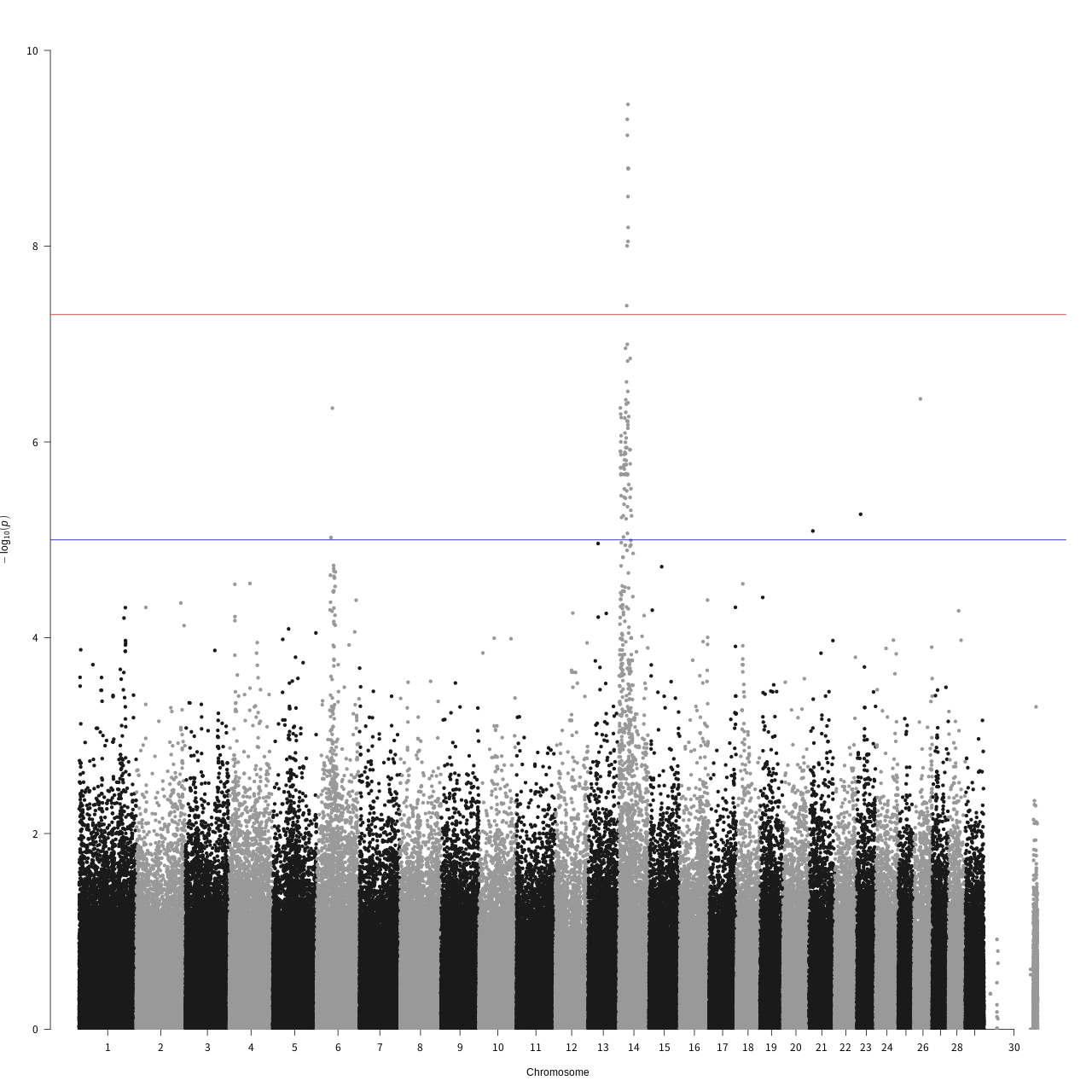

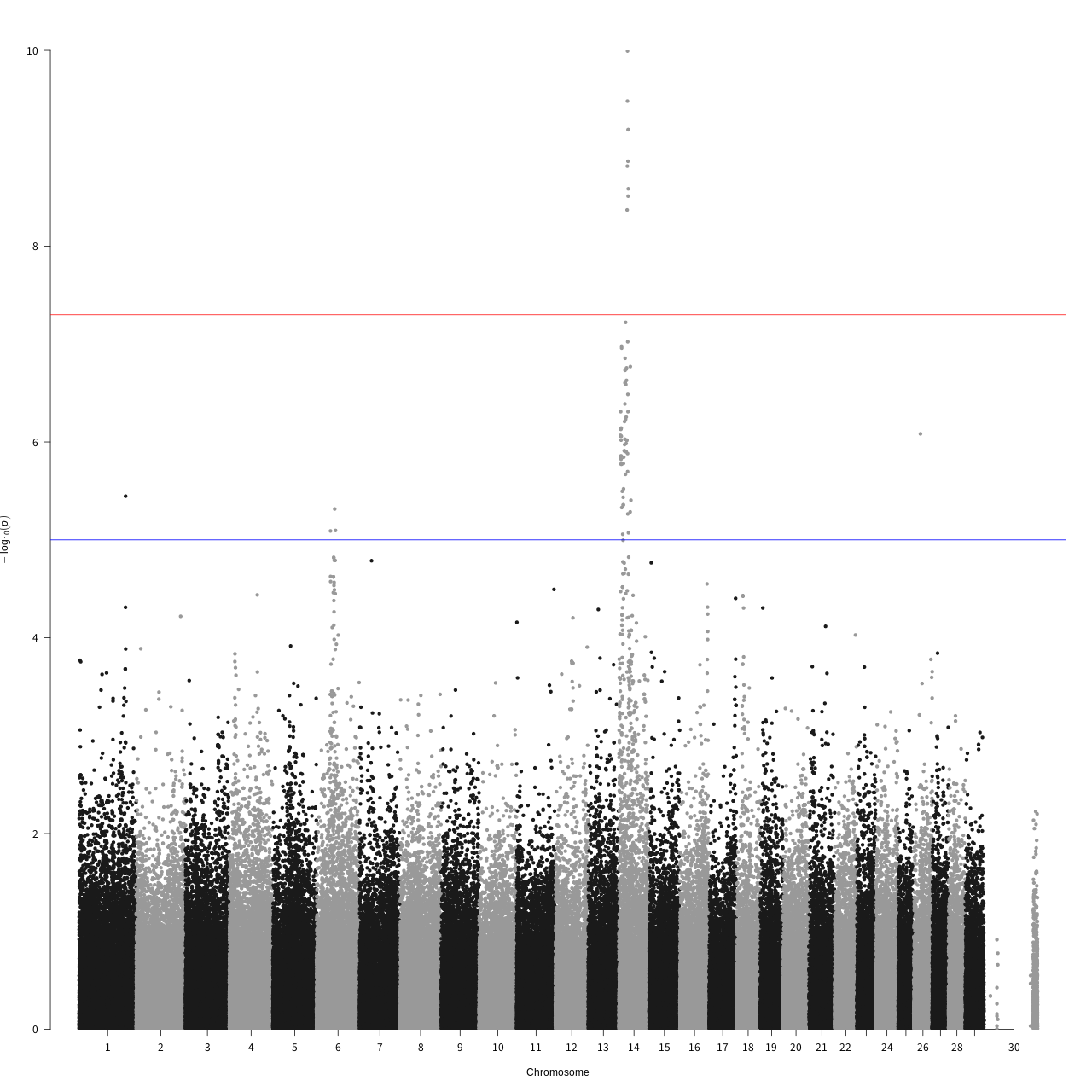


cwt_len_50_gemma cwt_len_100_gemma cwt_len_200_gemma


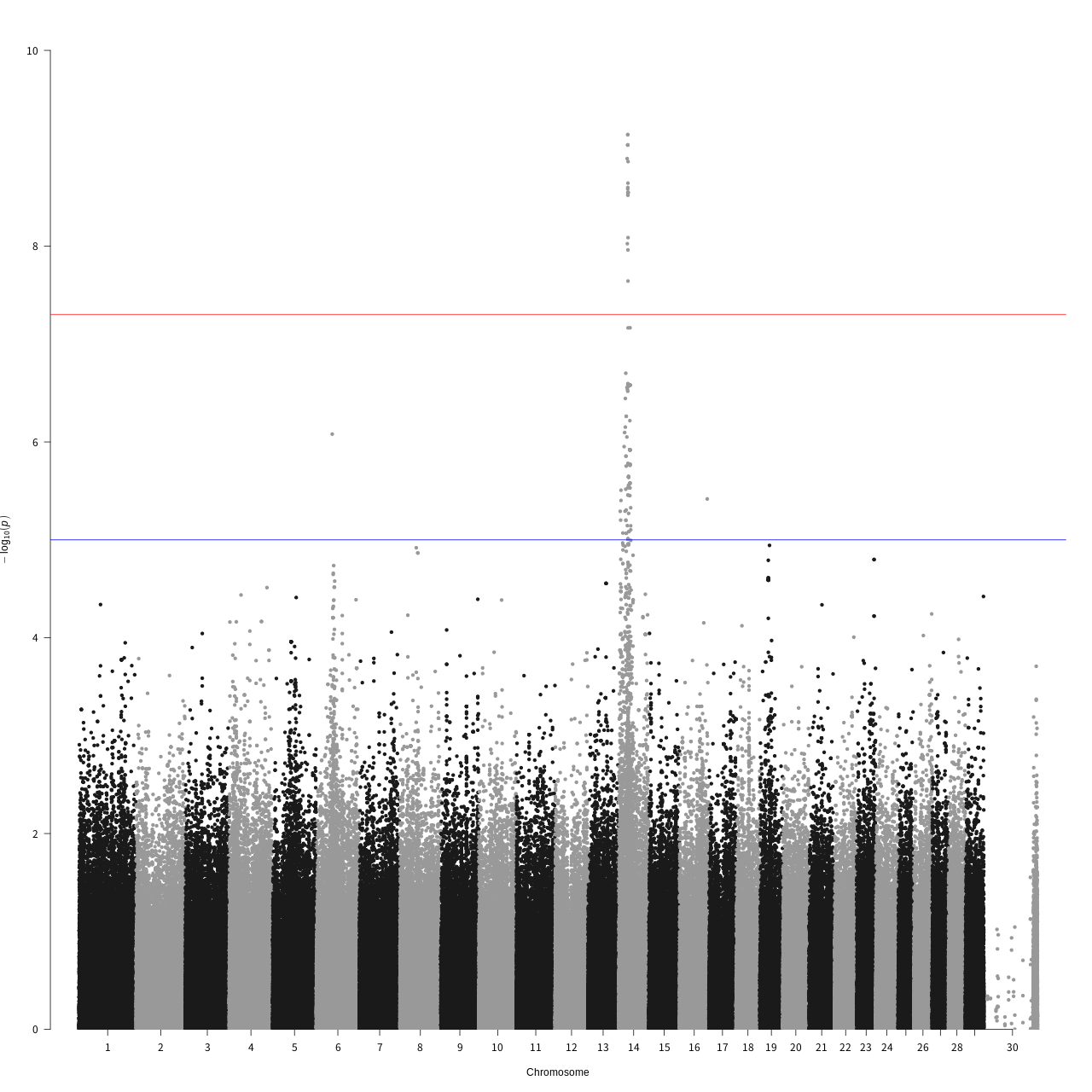

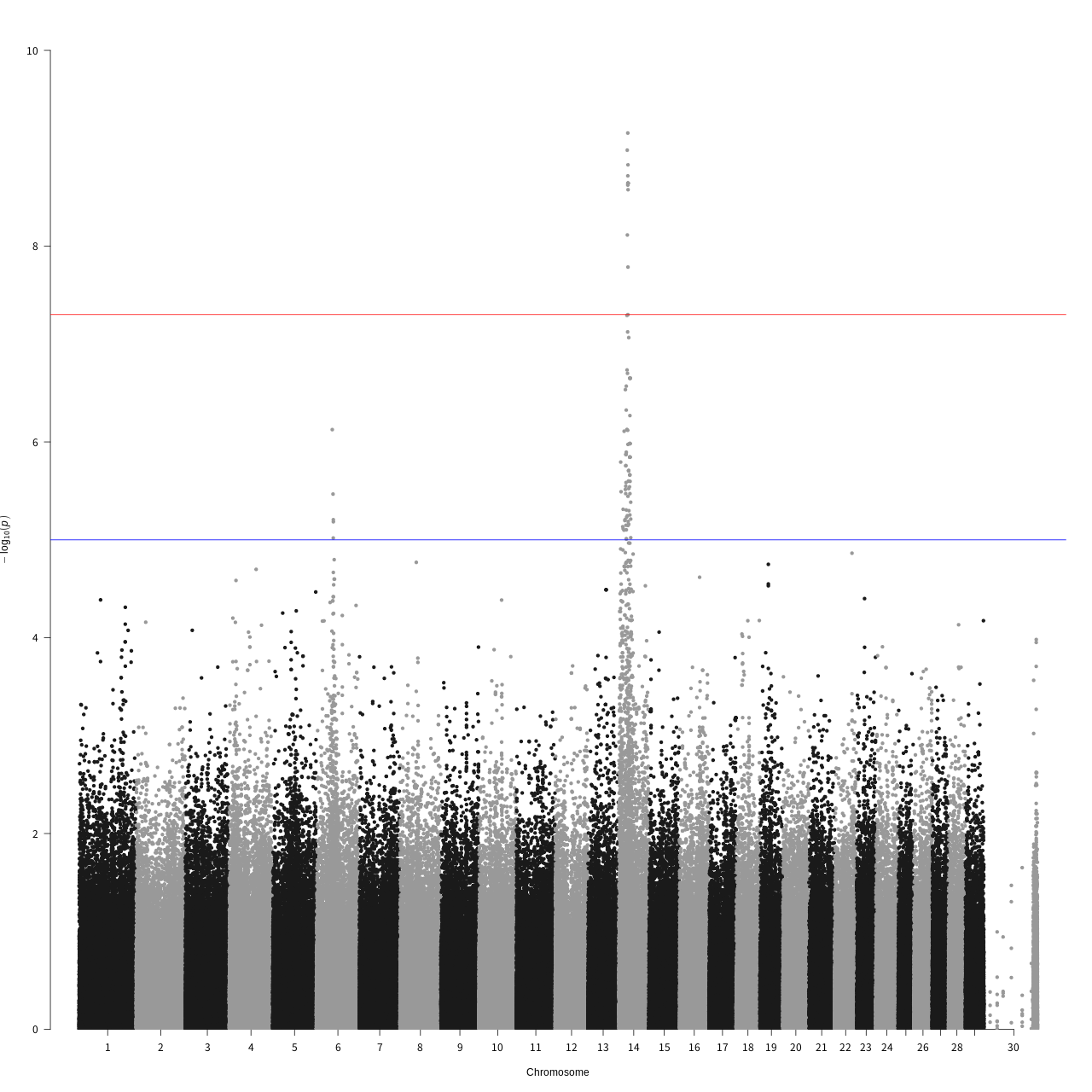

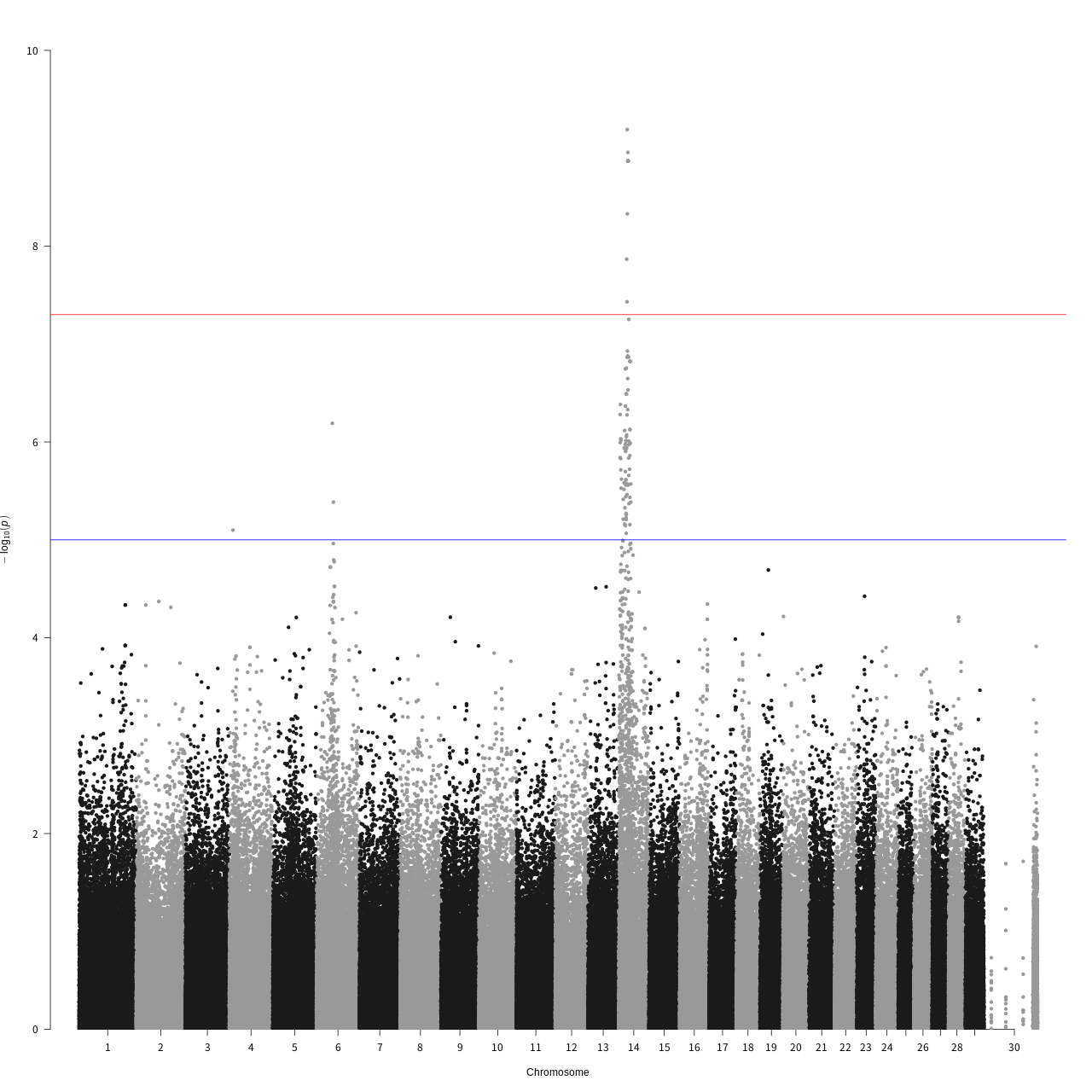


cwt_nsnp_2_gemma cwt_nsnp_5_gemma cwt_nsnp_10_gemma


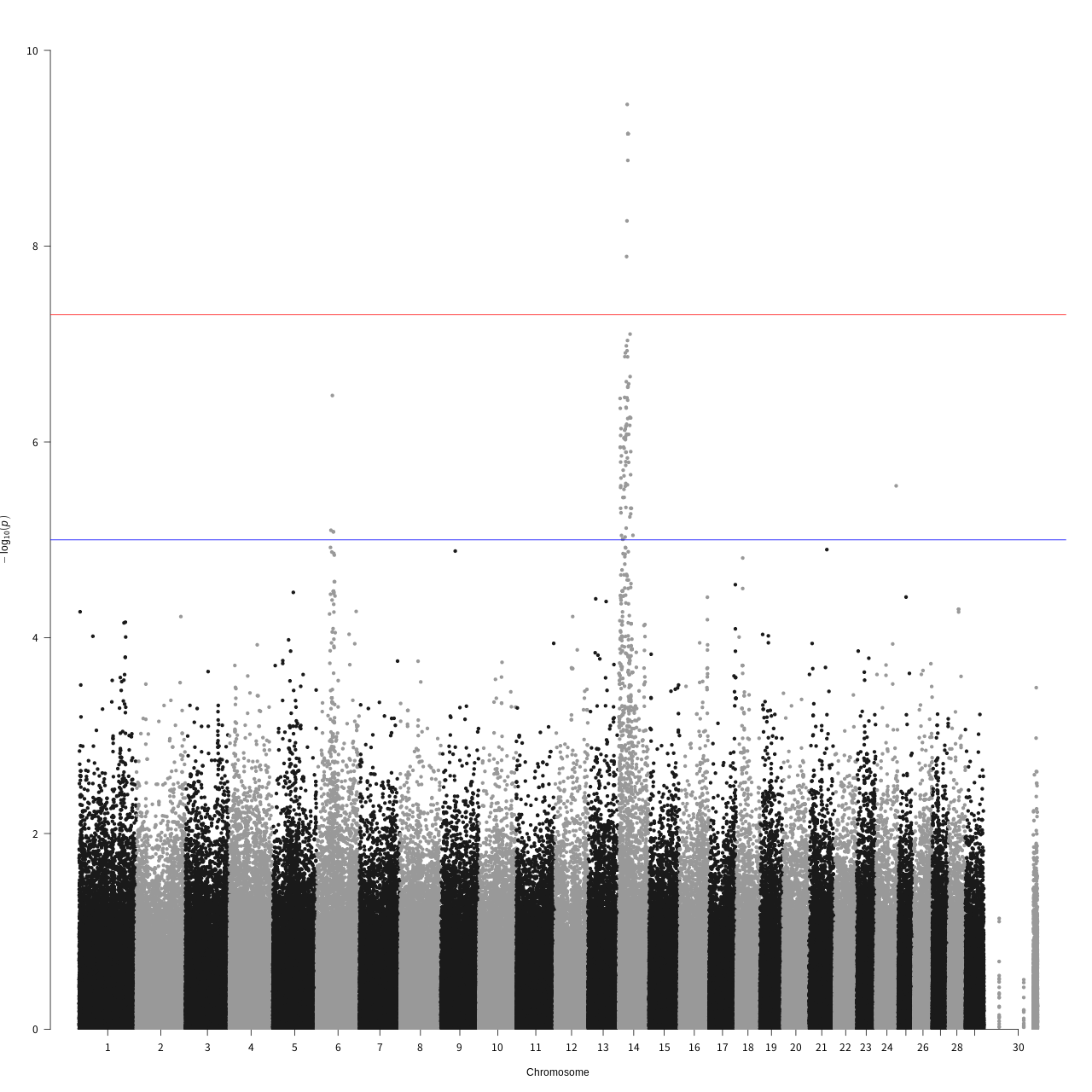

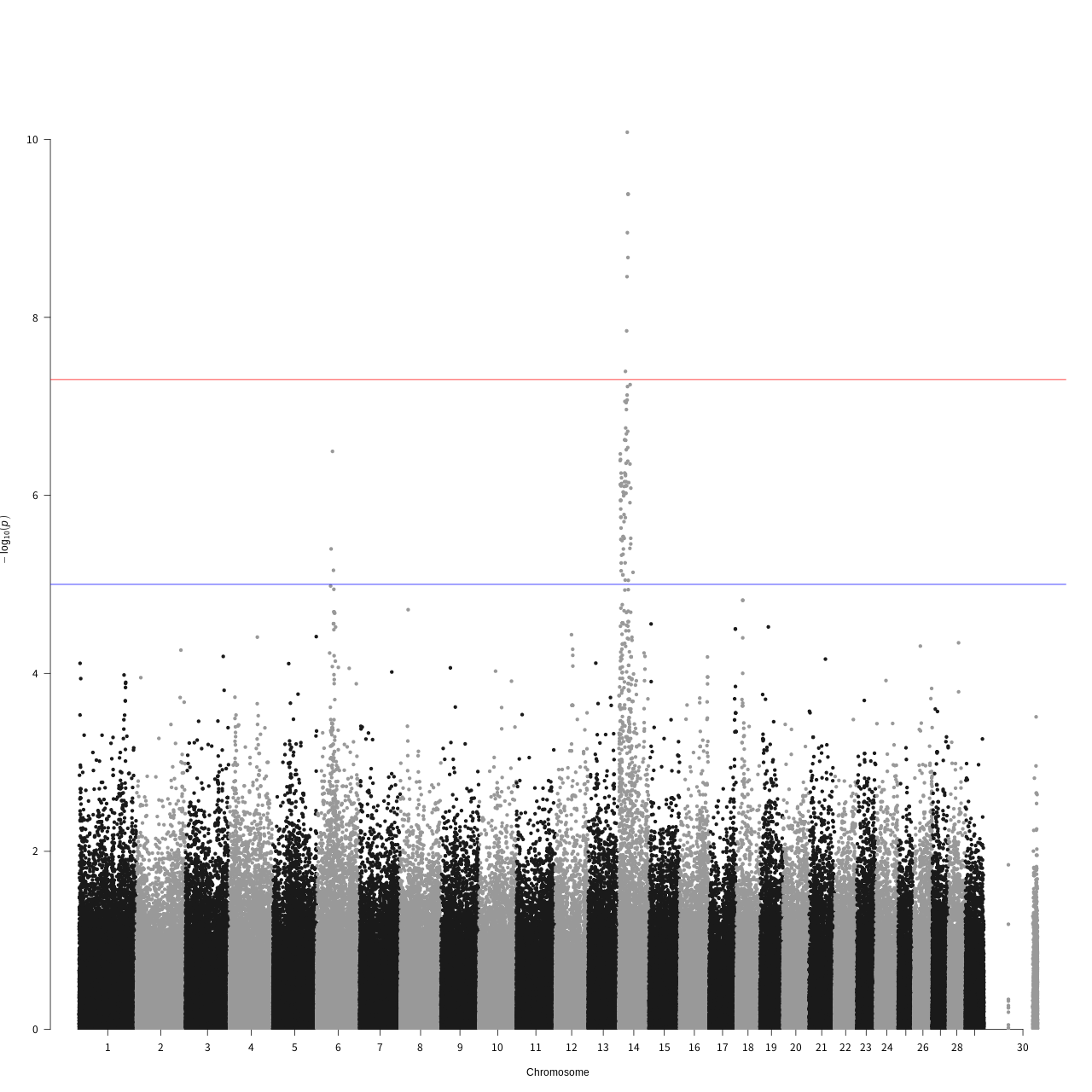

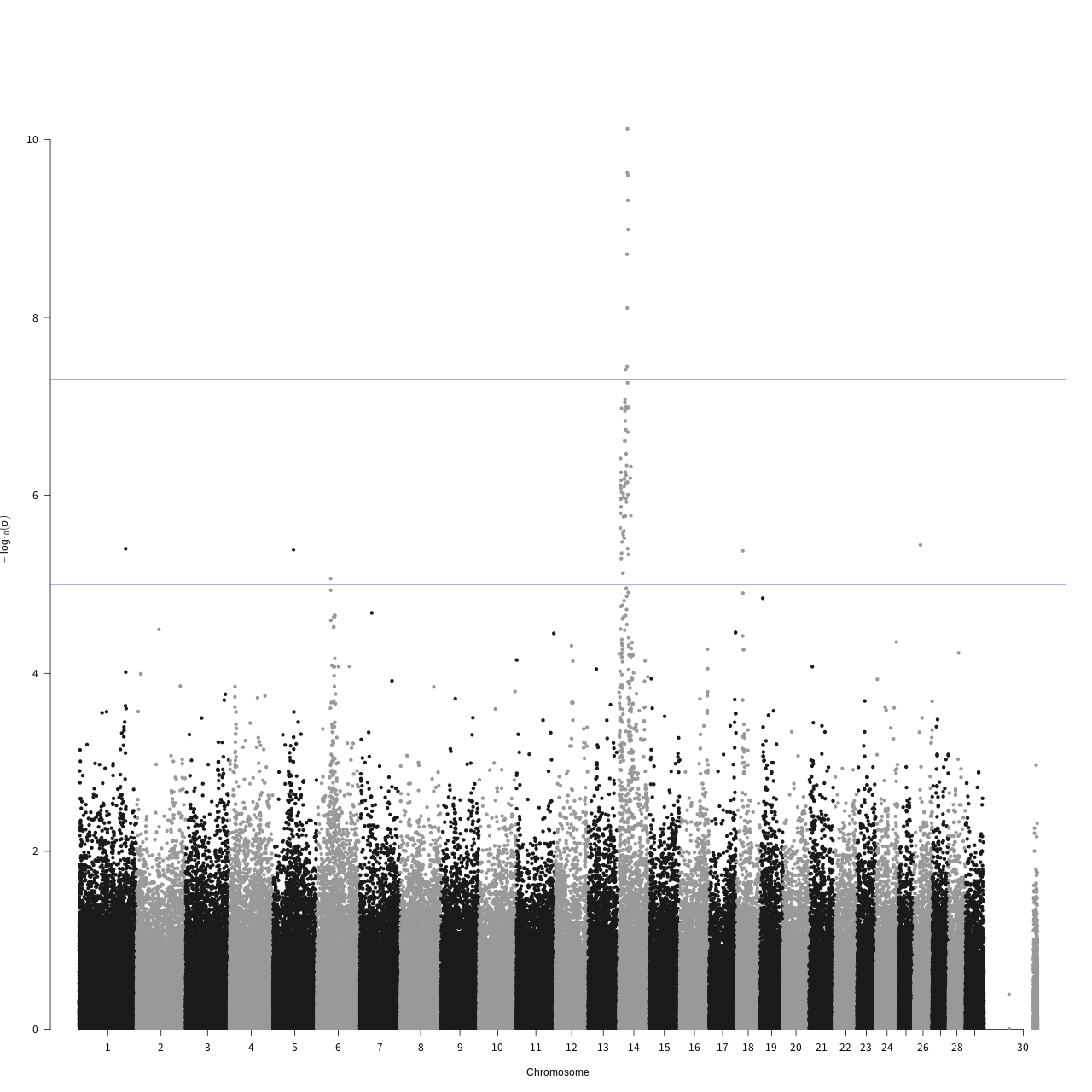


cwt_nsnp_20_gemma cwt_nsnp_30_gemma cwt_nsnp_50_gemma

1. Manhattan for EMA


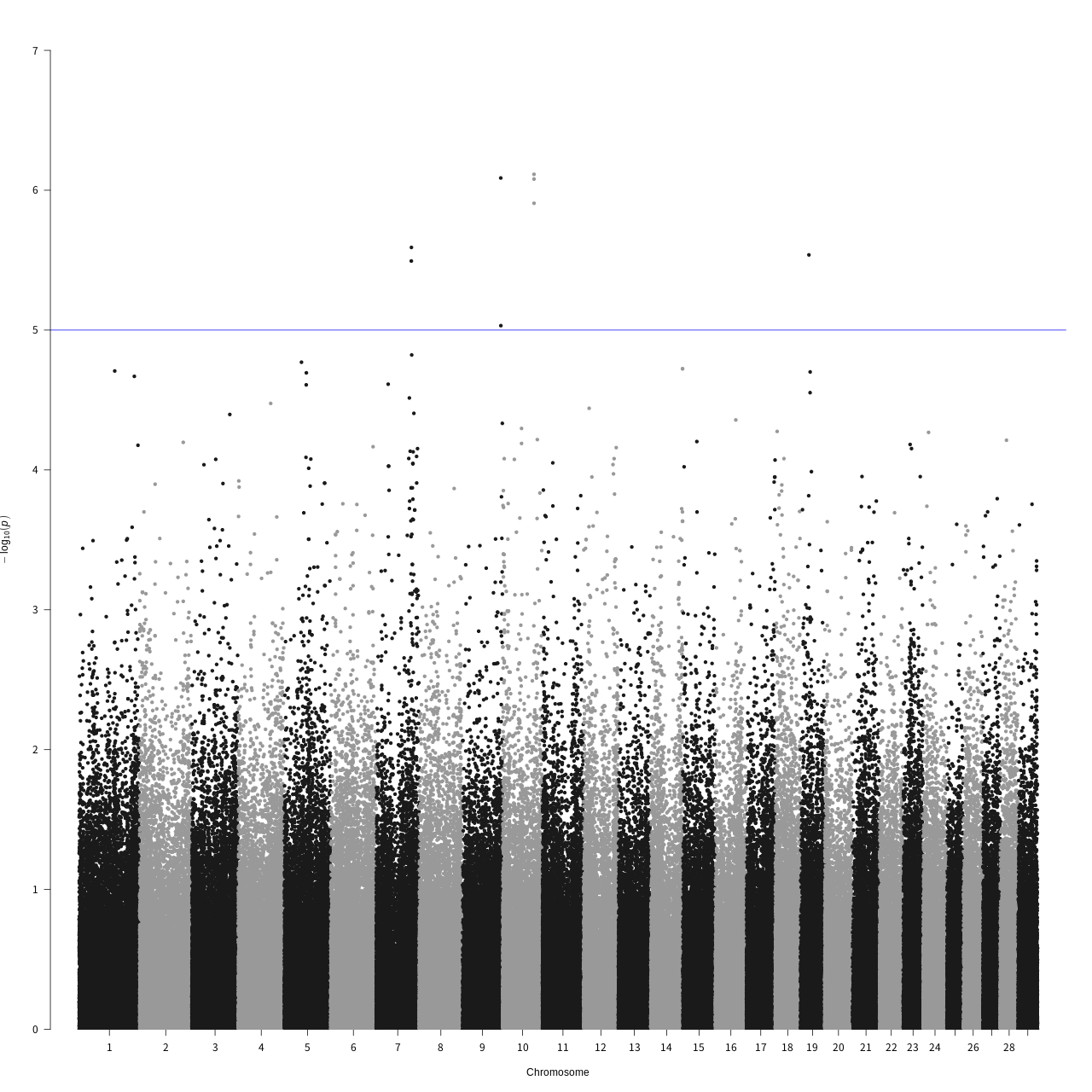

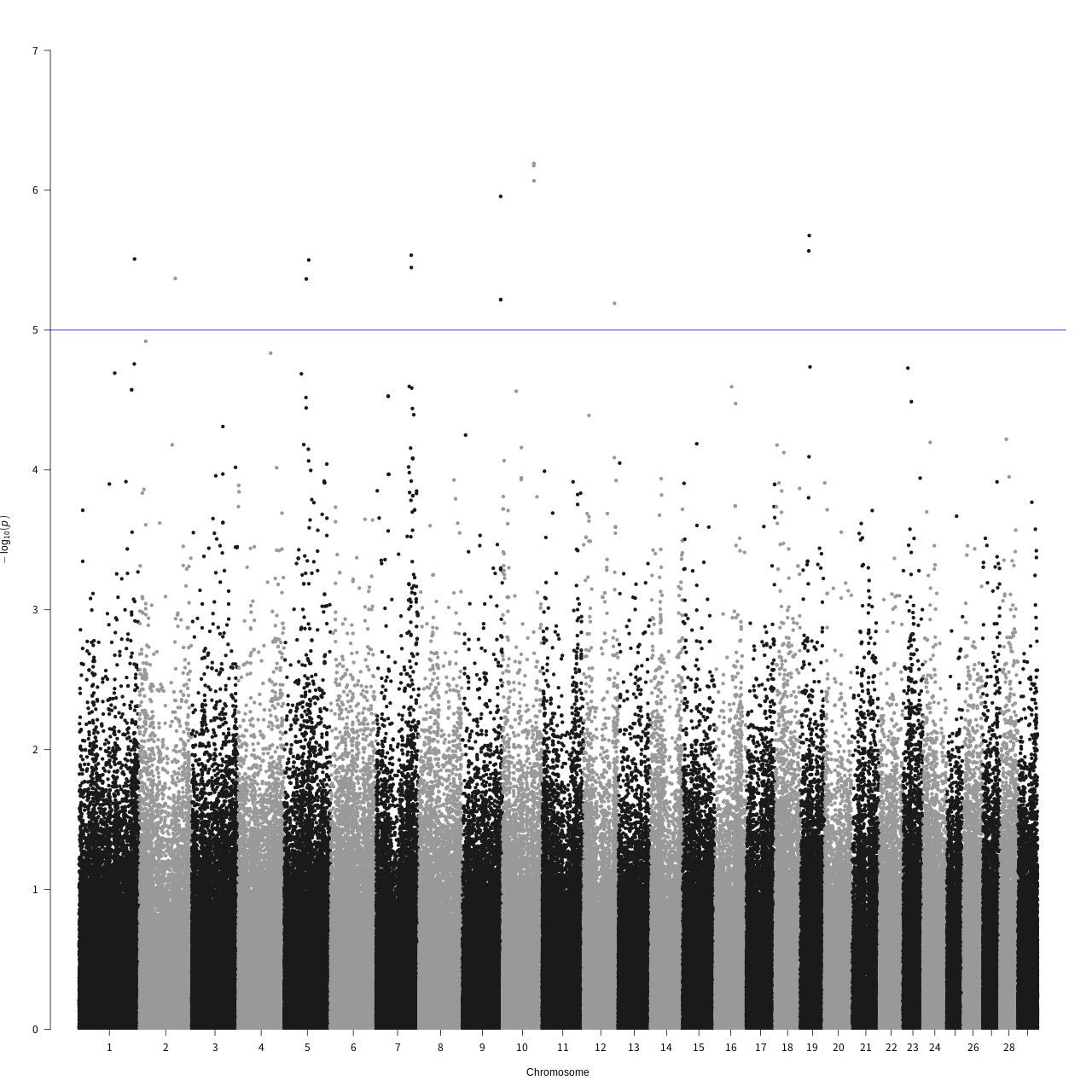

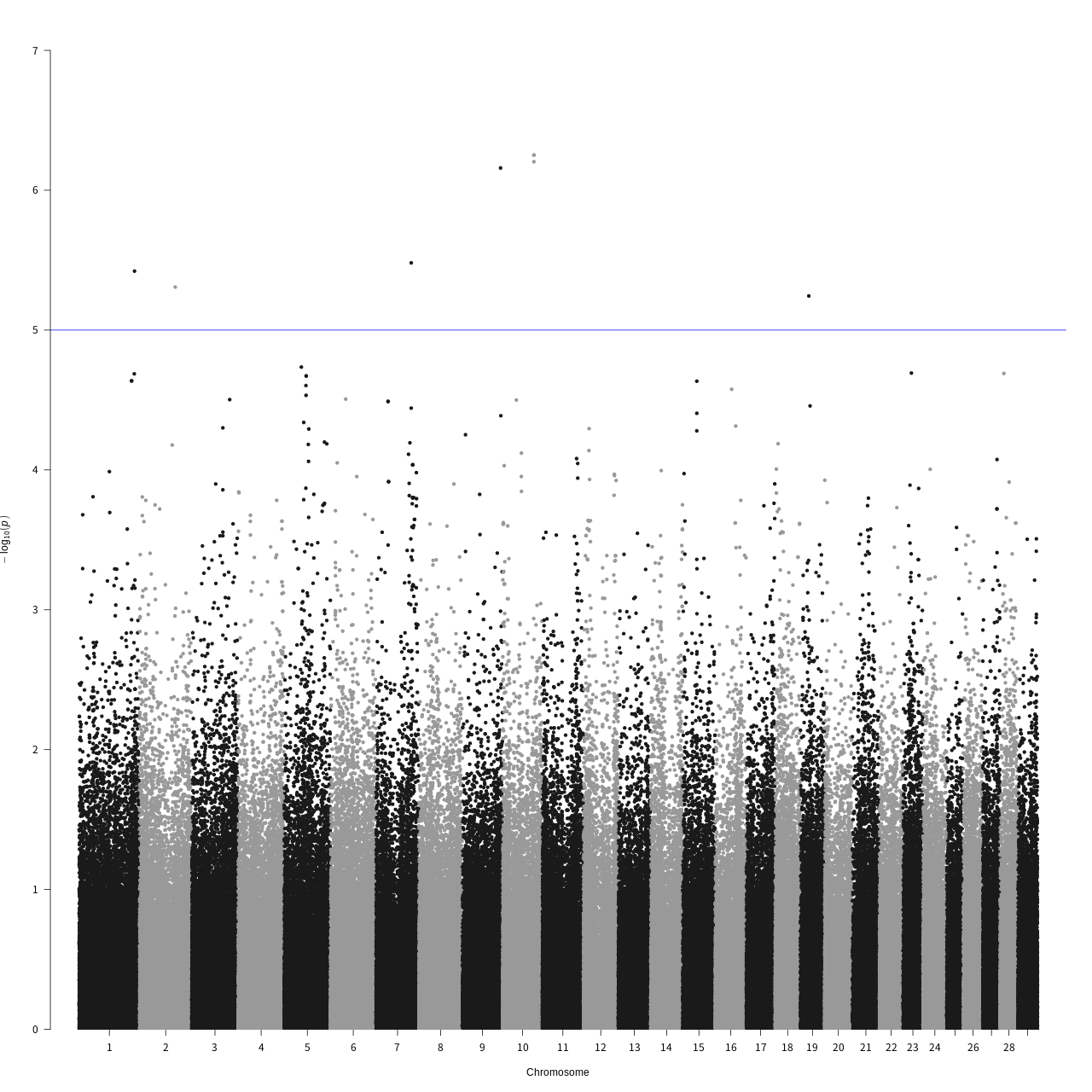


ema_ld_0.2_gemma ema_ld_0.3_gemma ema_ld_0.4_gemma


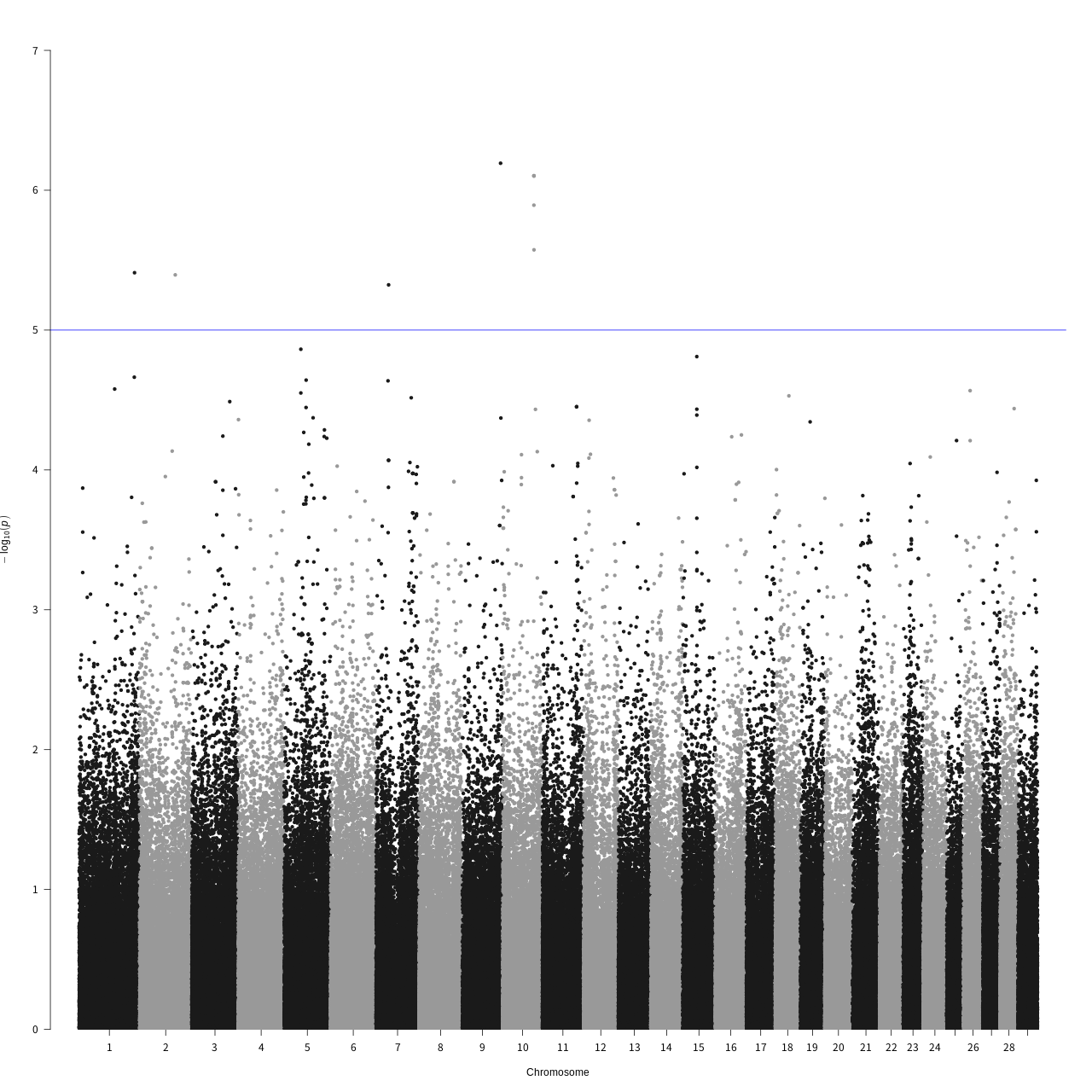

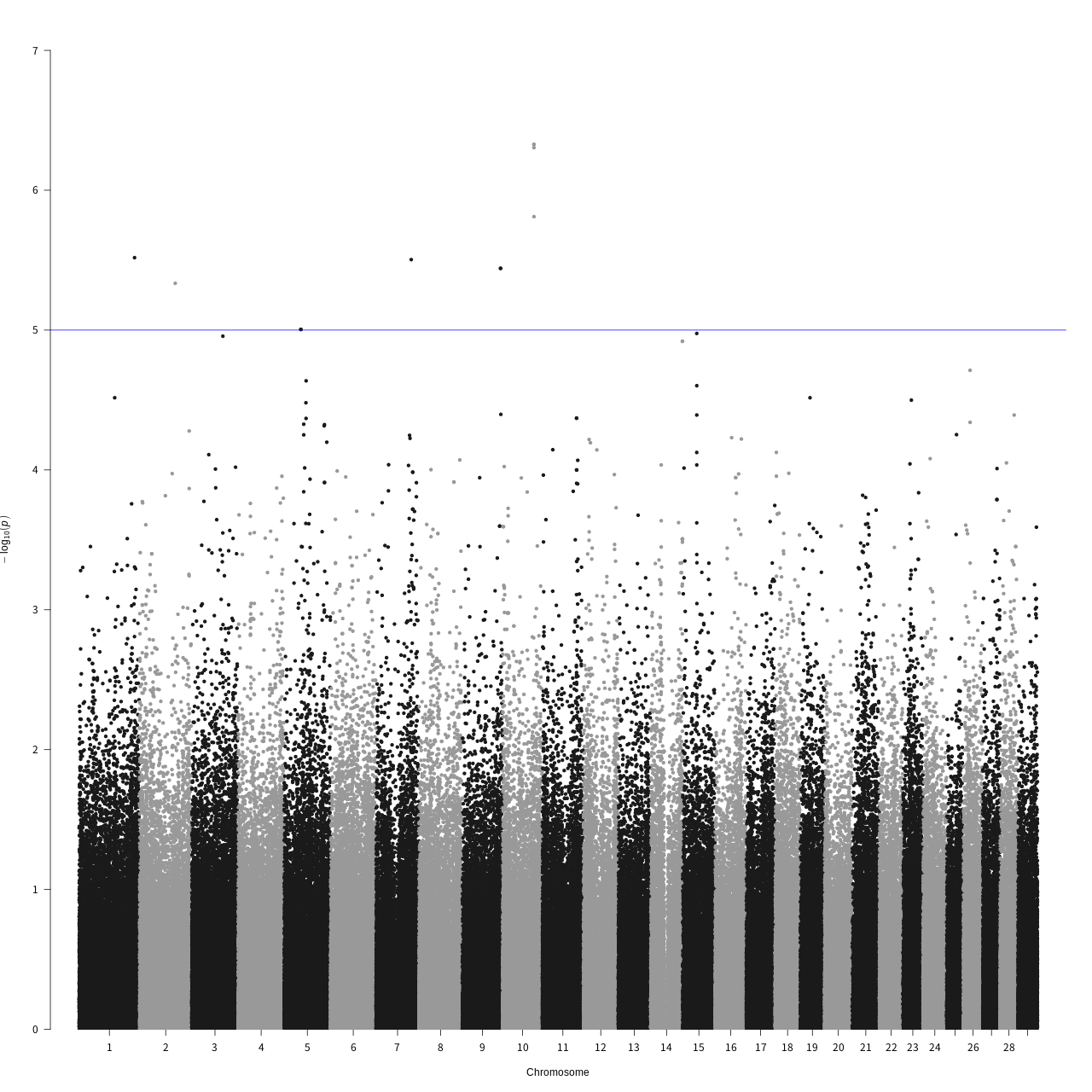

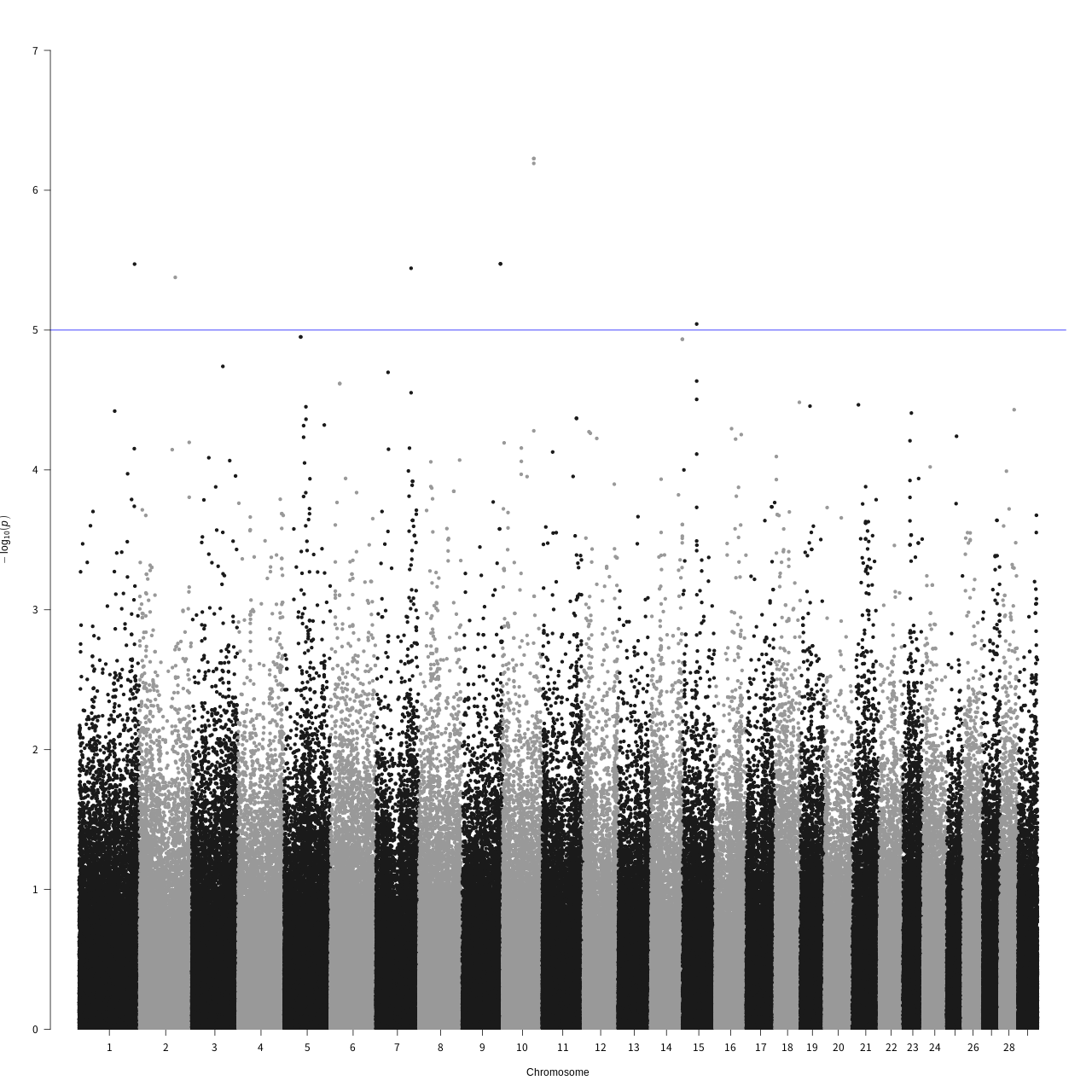


ema_ld_0.5_gemma ema_ld_0.6_gemma ema_ld_0.7_gemma


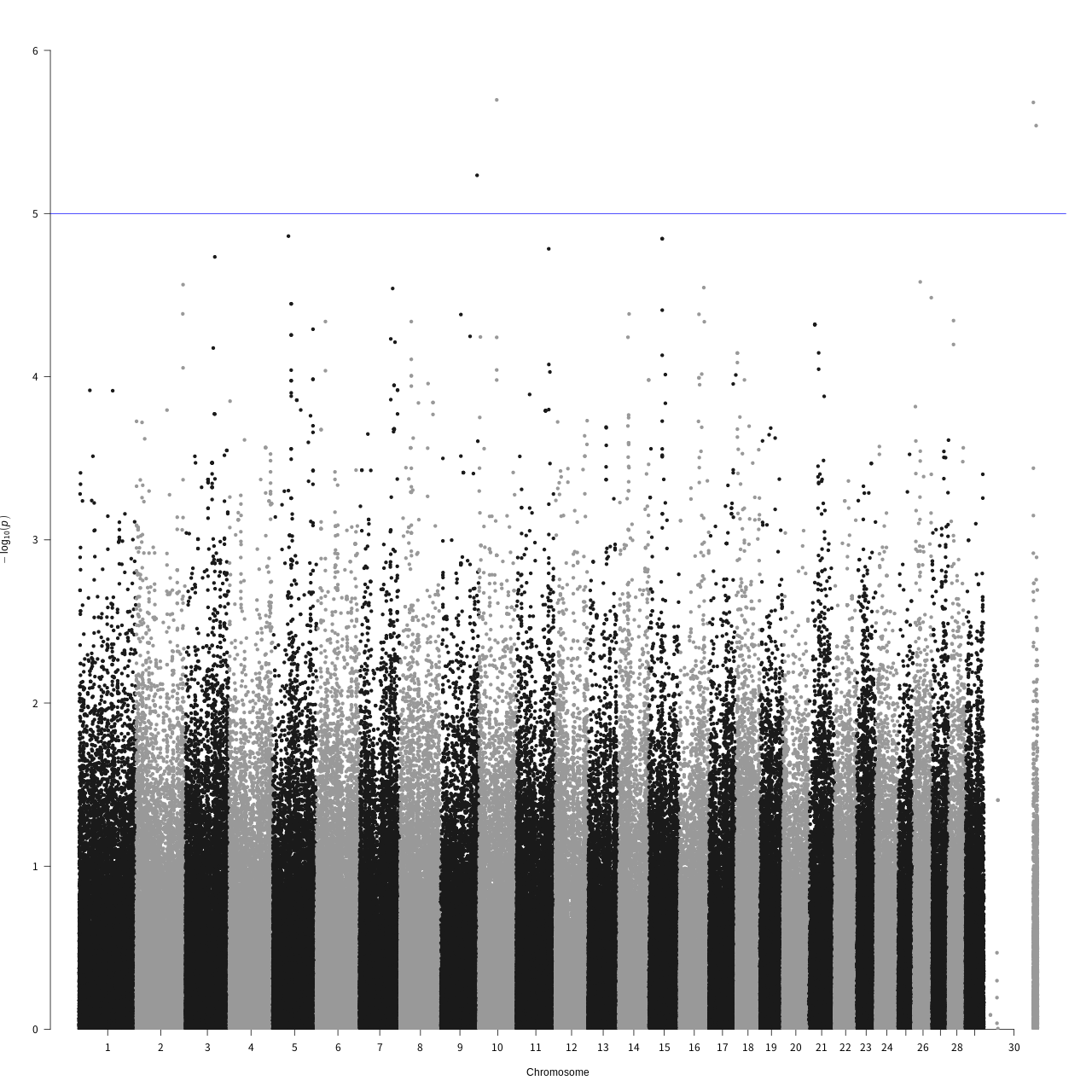

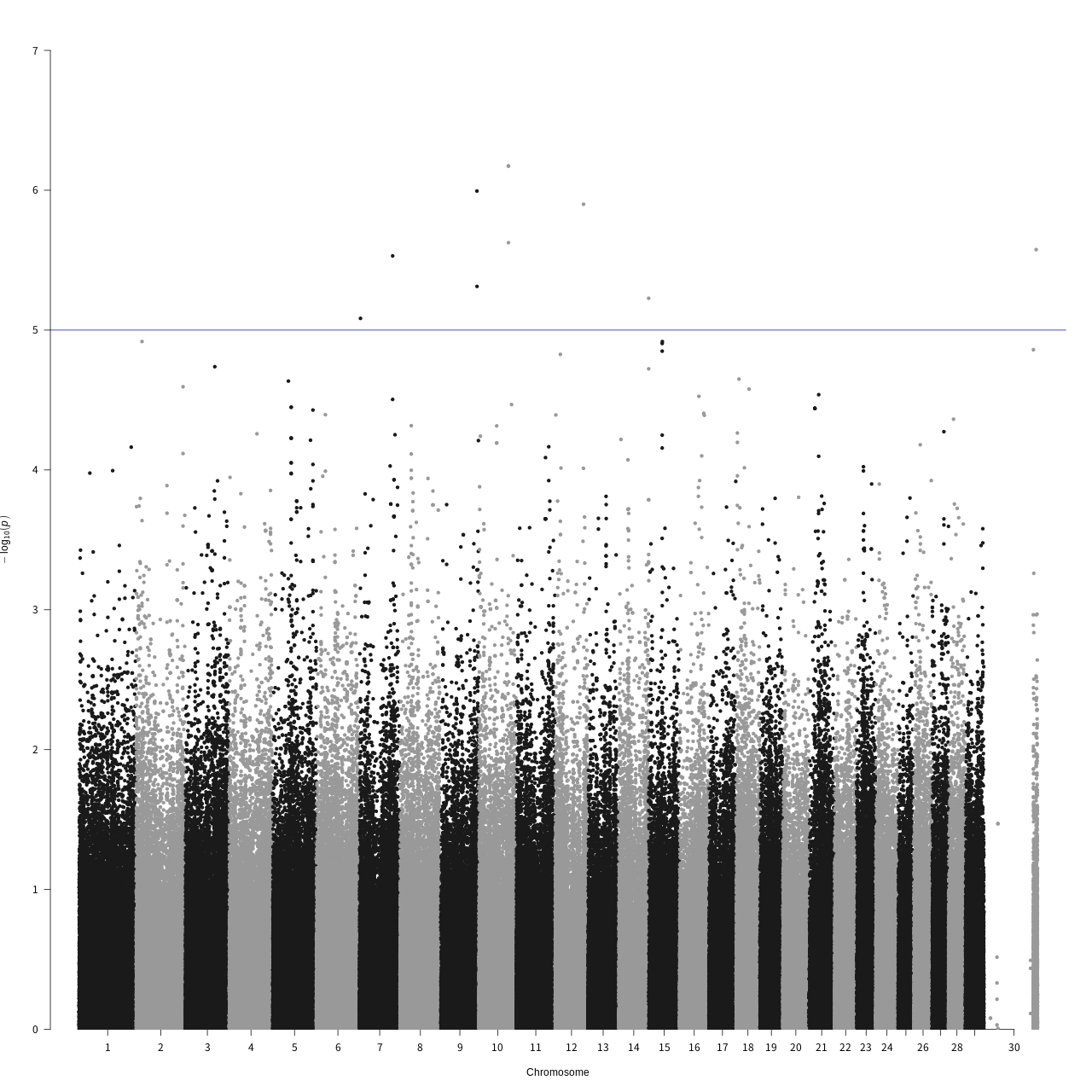

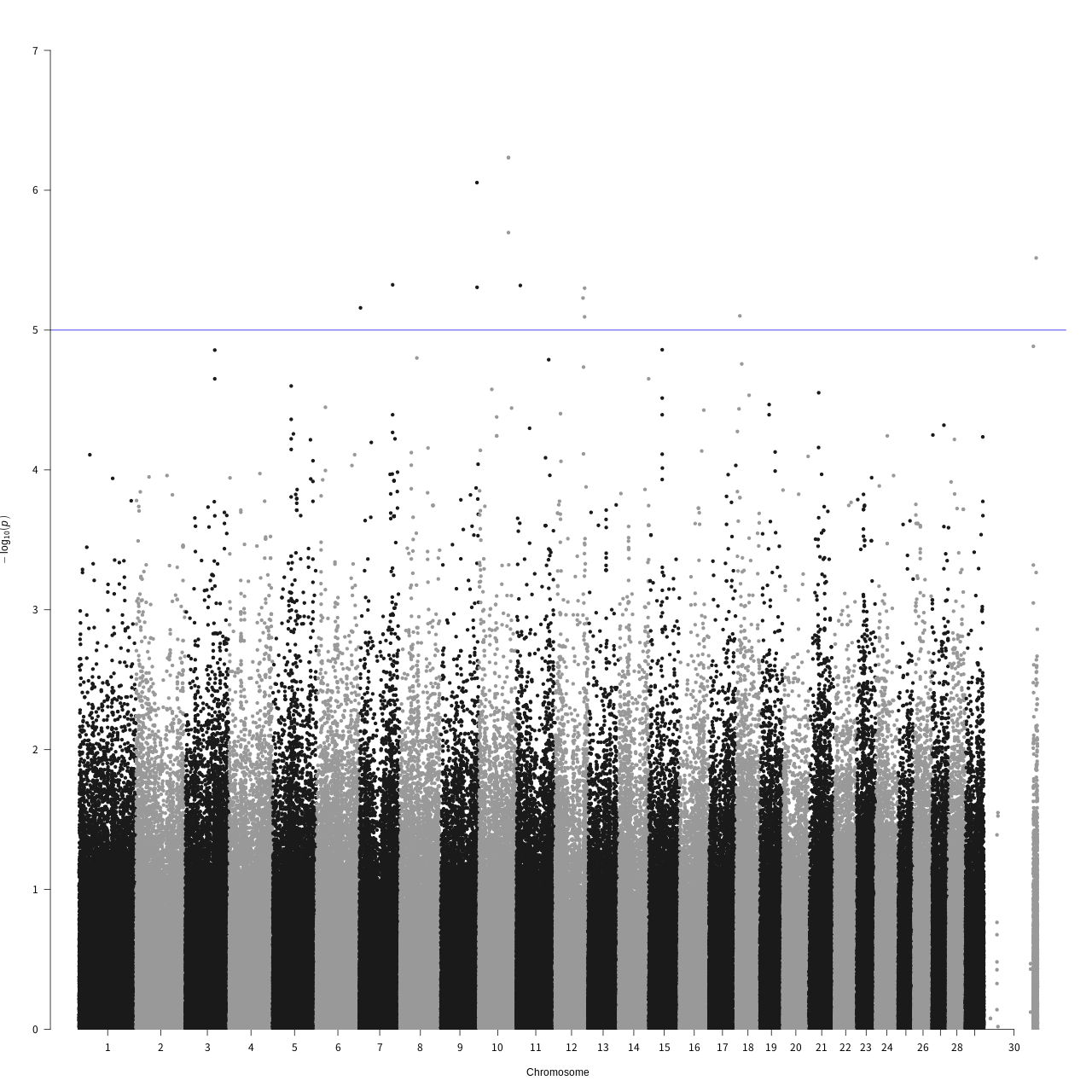


ema_len_5_gemma ema_len_10_gemma ema_len_20_gemma


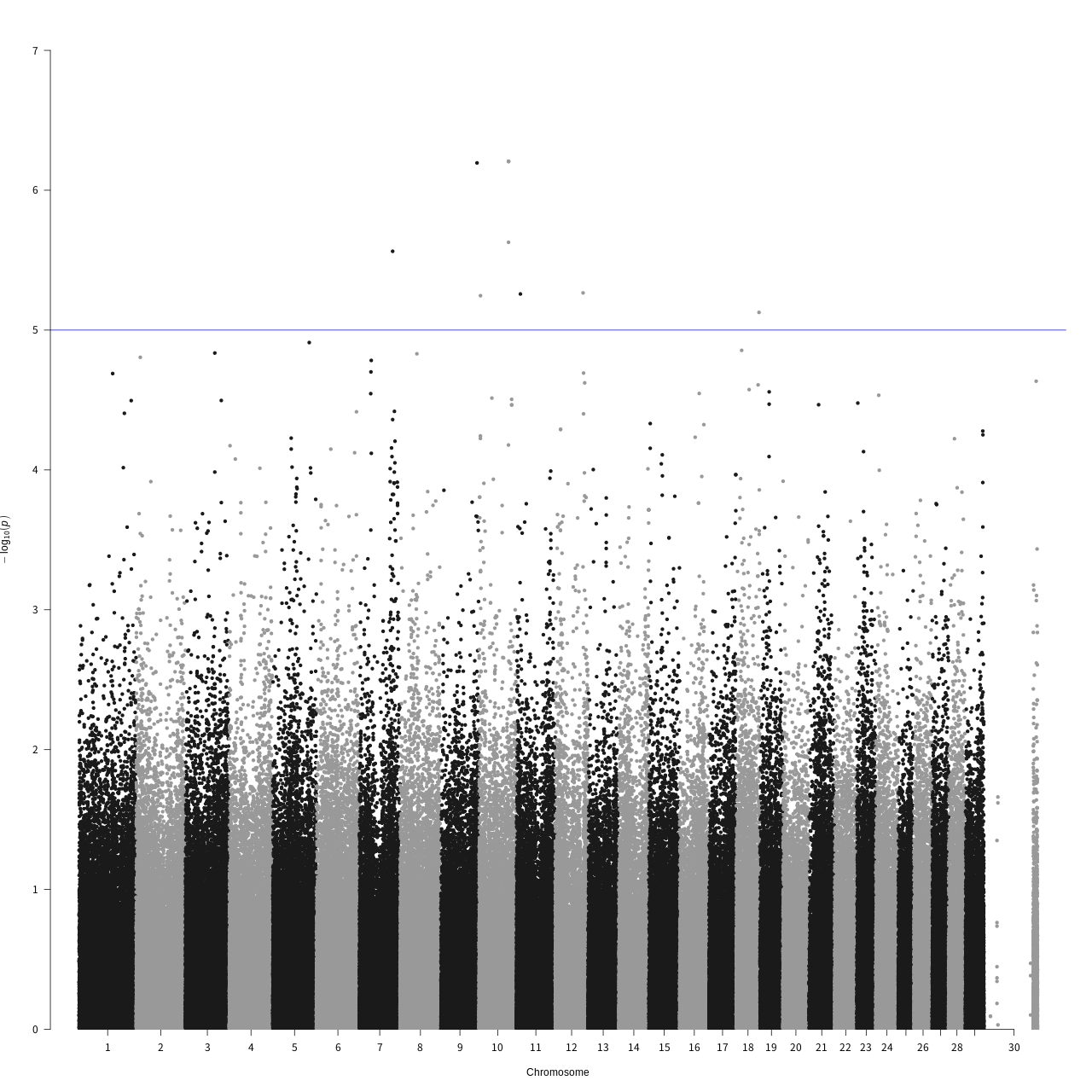

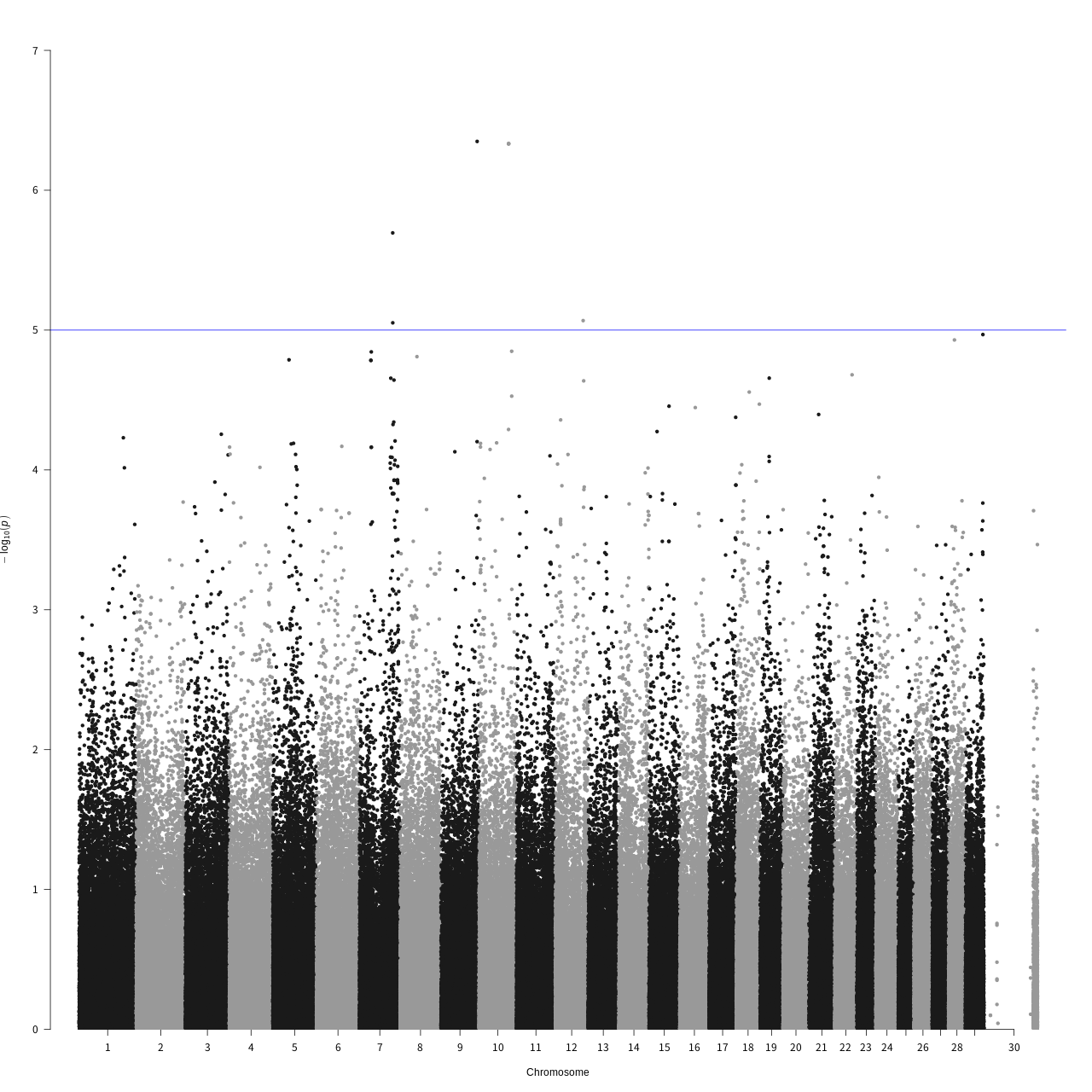

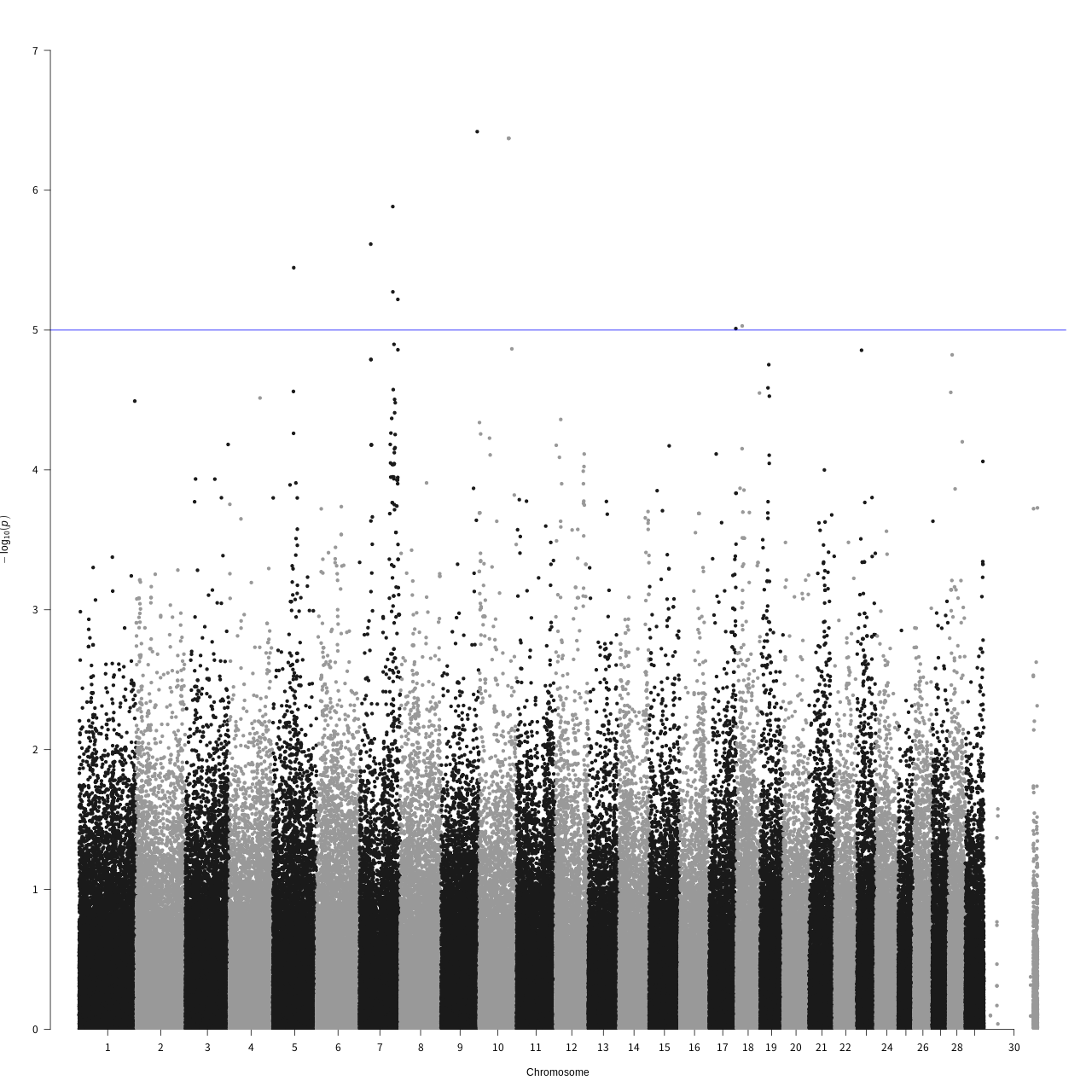


ema_len_50_gemma ema_len_100_gemma ema_len_200_gemma


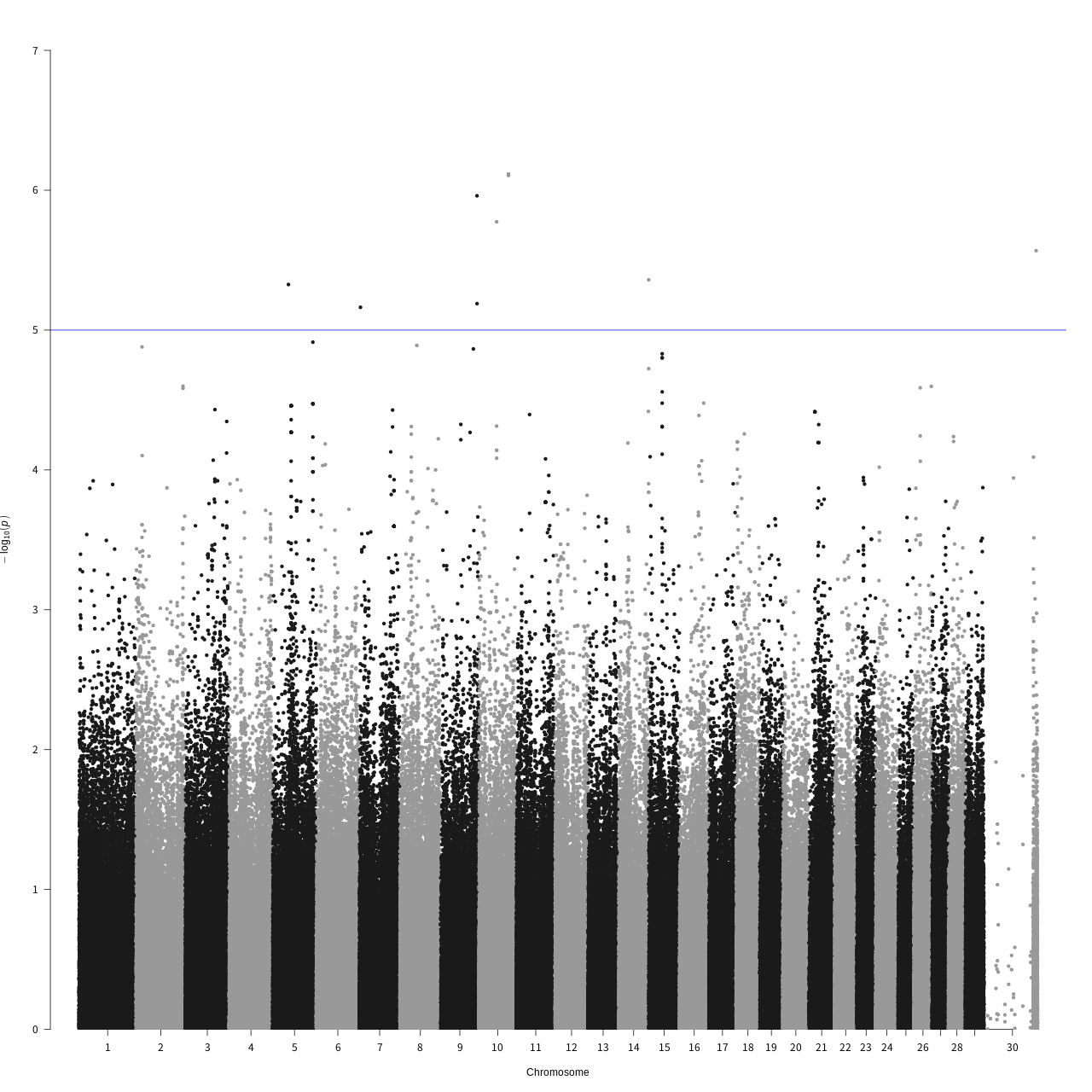

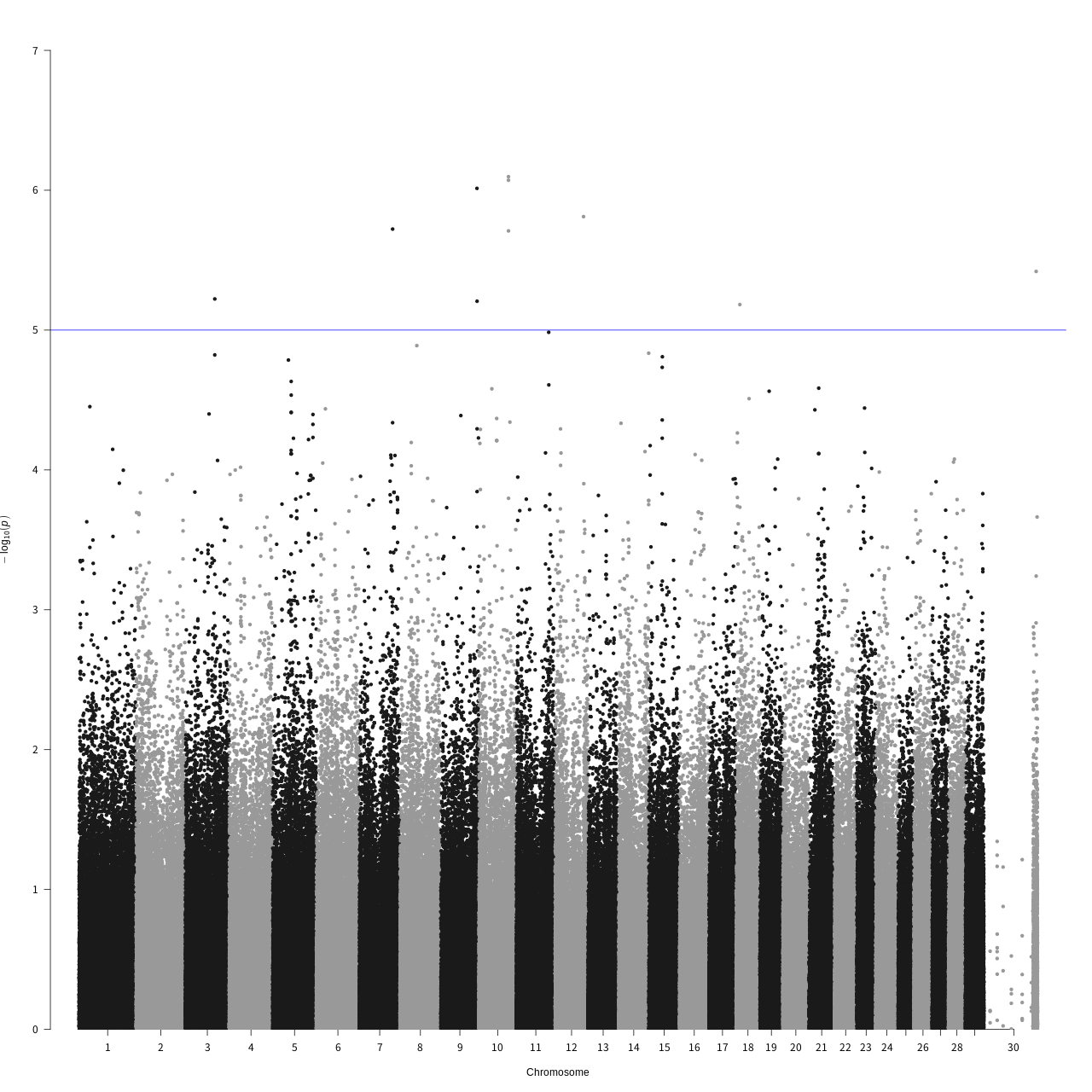

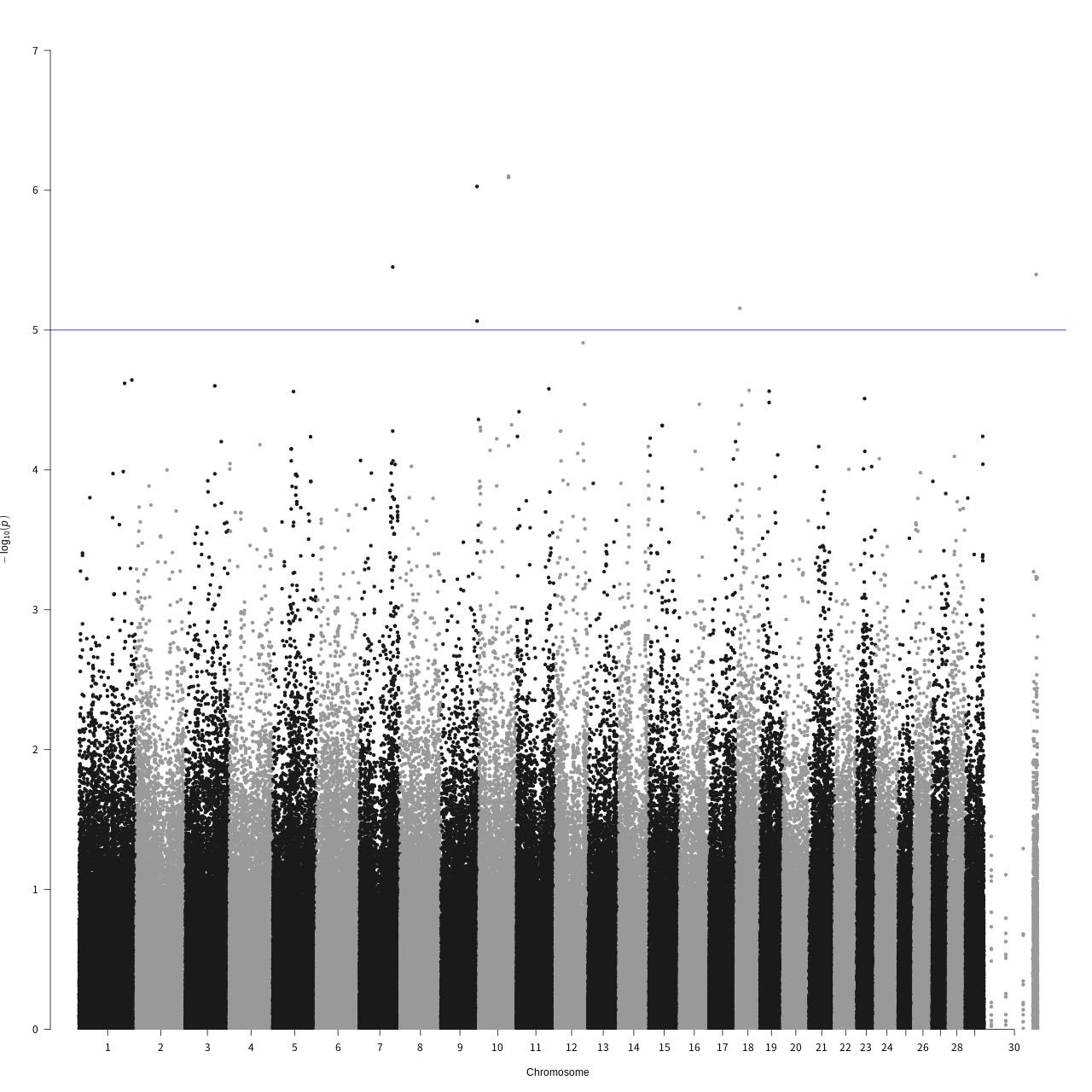


ema_nsnp_2_gemma ema_nsnp_5_gemma ema_nsnp_10_gemma


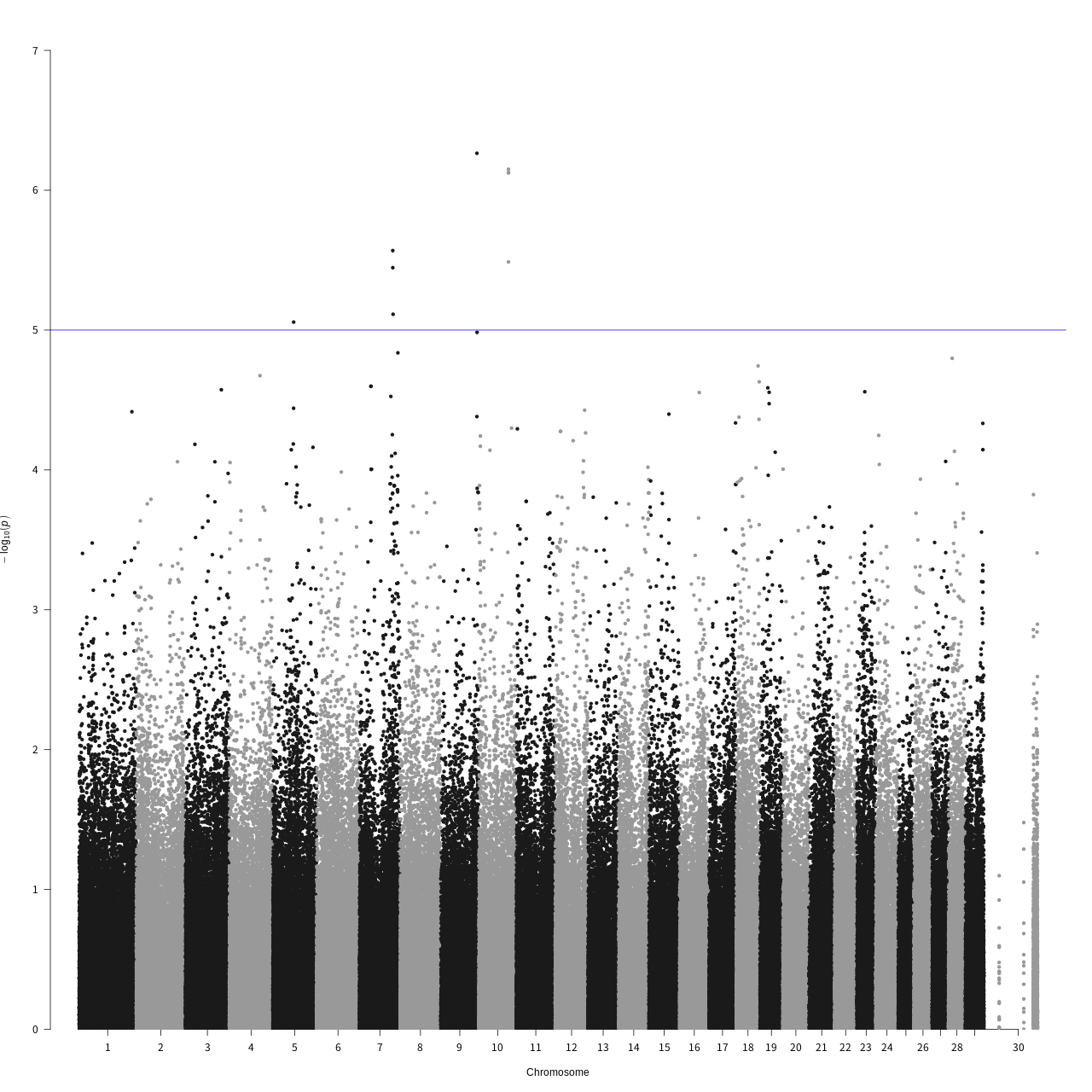

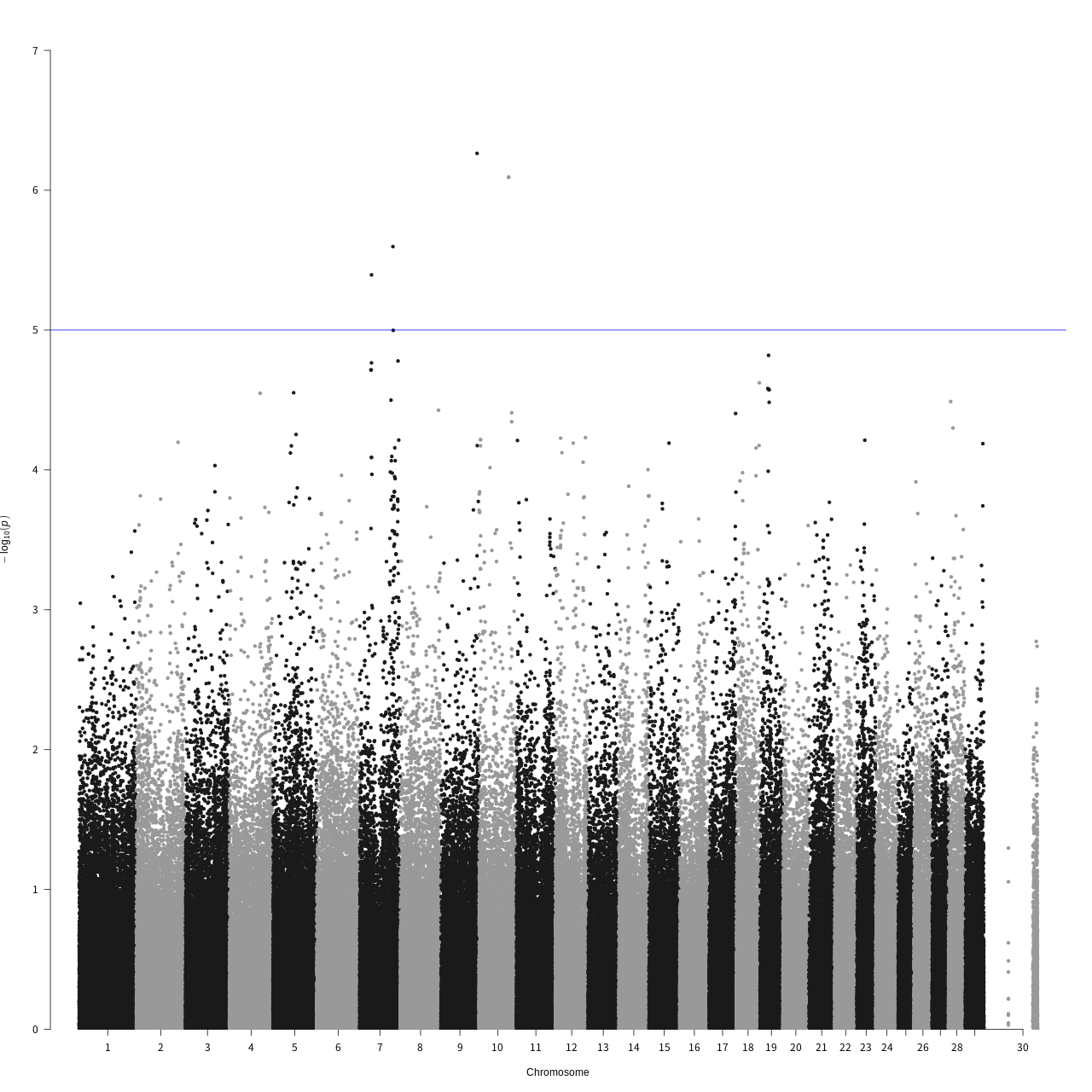

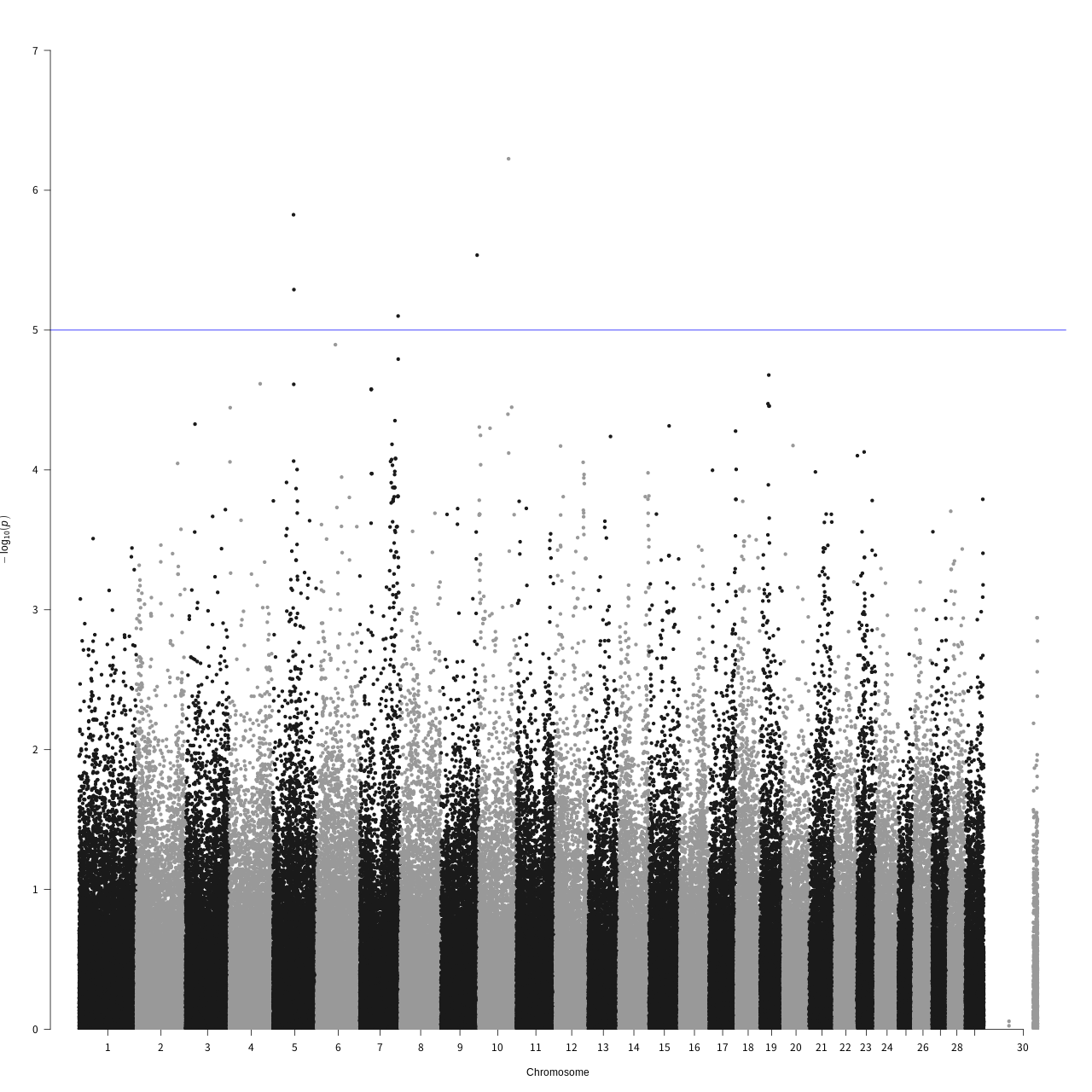


ema_nsnp_20_gemma ema_nsnp_30_gemma ema_nsnp_50_gemma

1. Manhattan plot for MS

ms_ld_0.2_gemma ms_ld_0.3_gemma ms_ld_0.4 _gemmams_ld_0.5_gemma ms_ld_0.6_gemma ms_ld_0.7_gemma


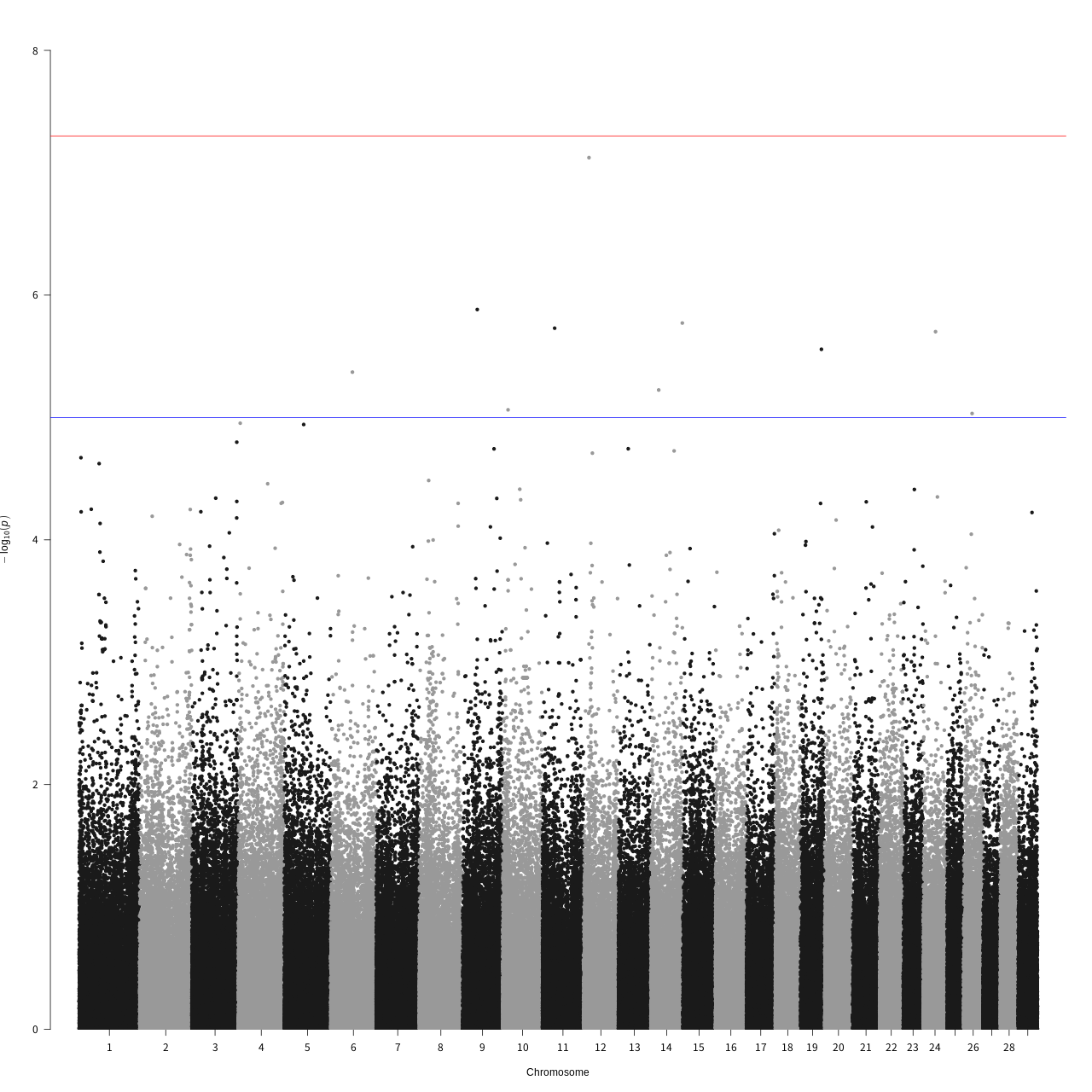

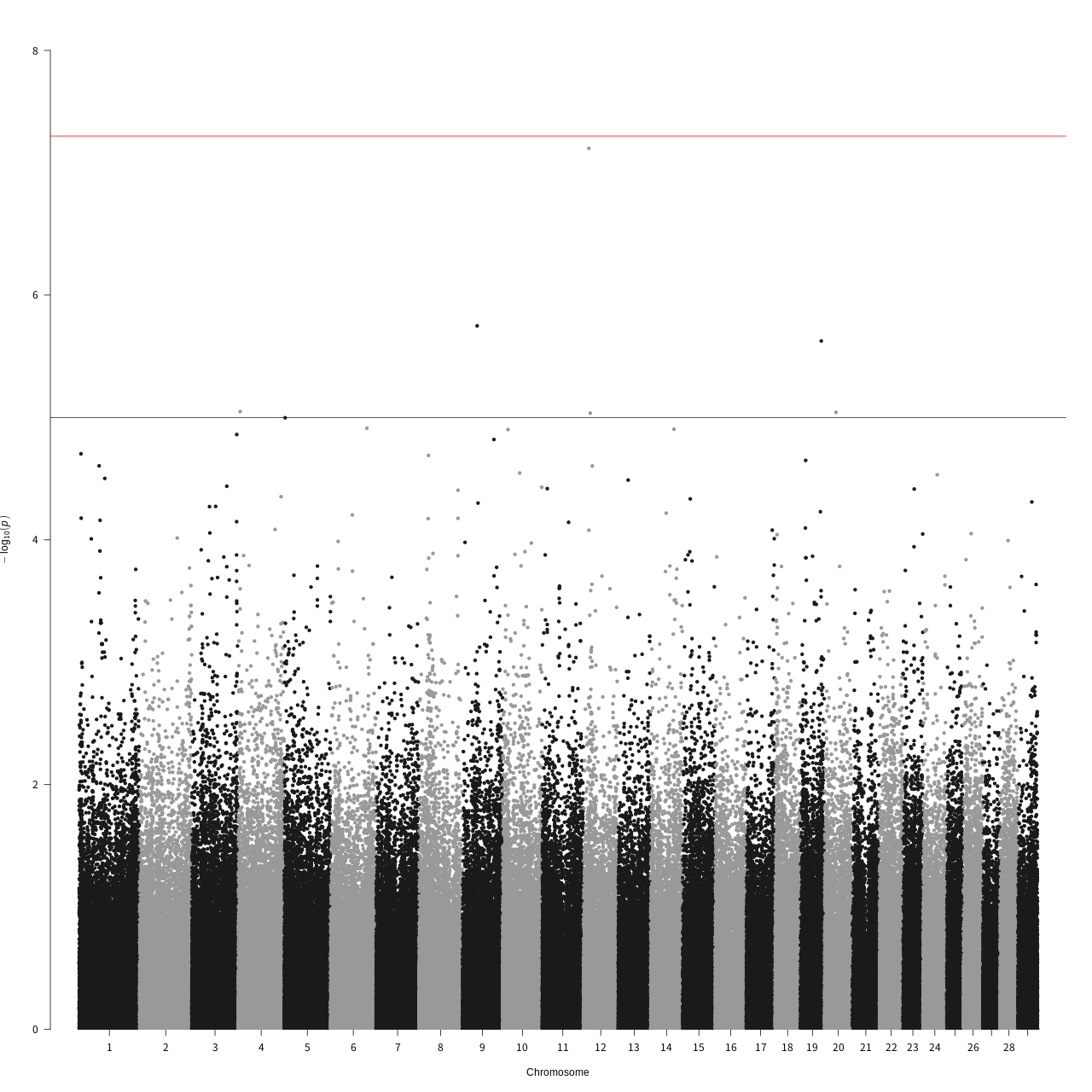

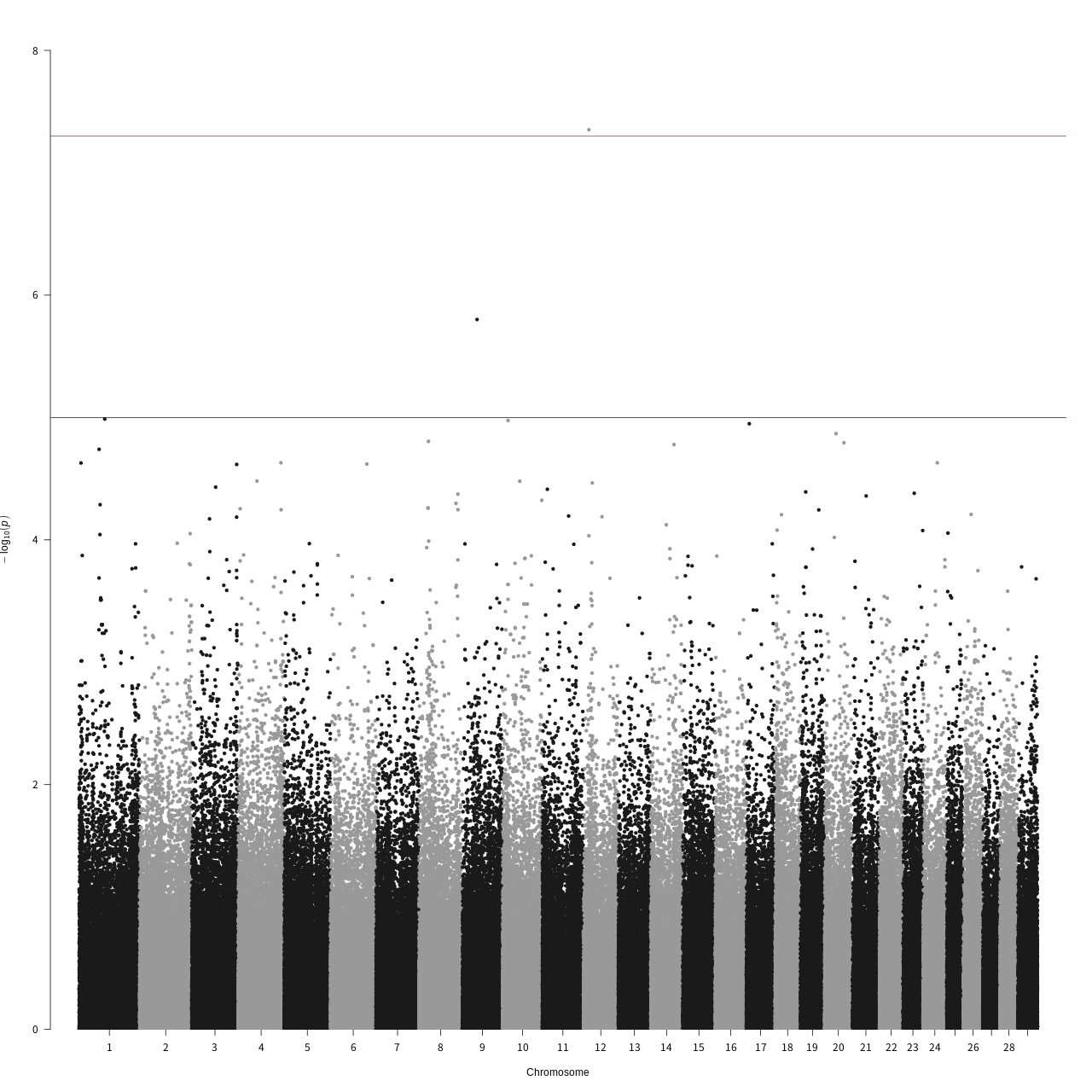

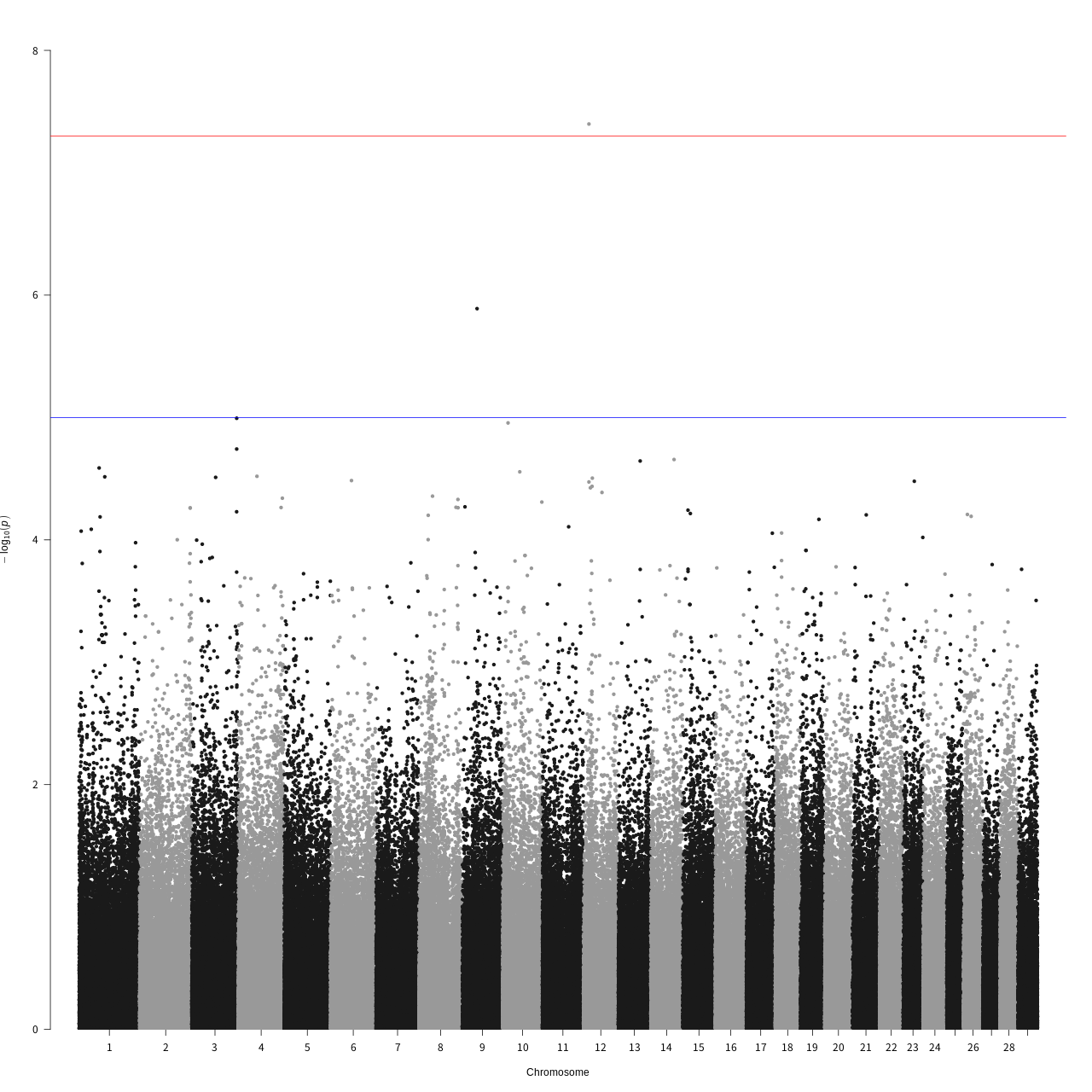

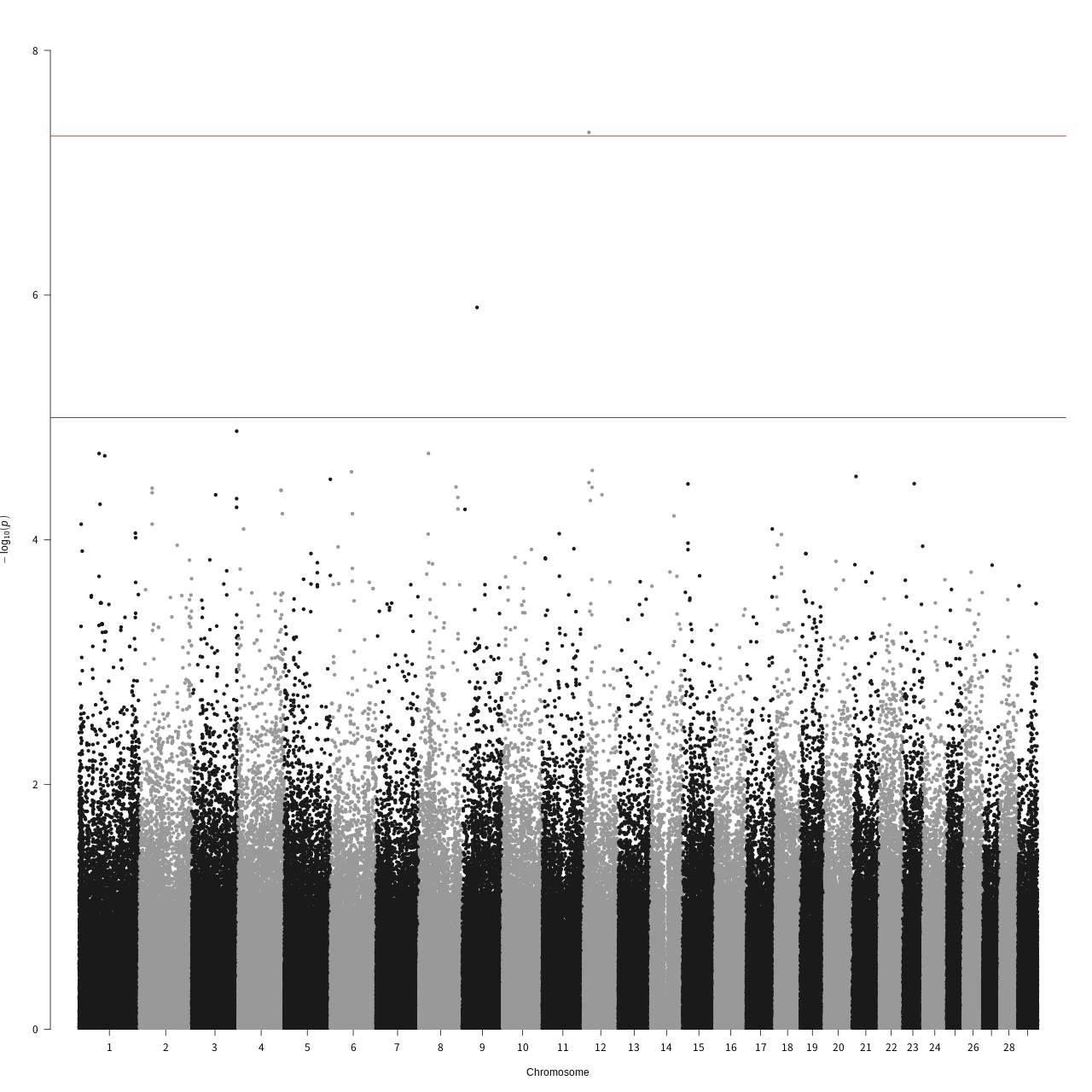

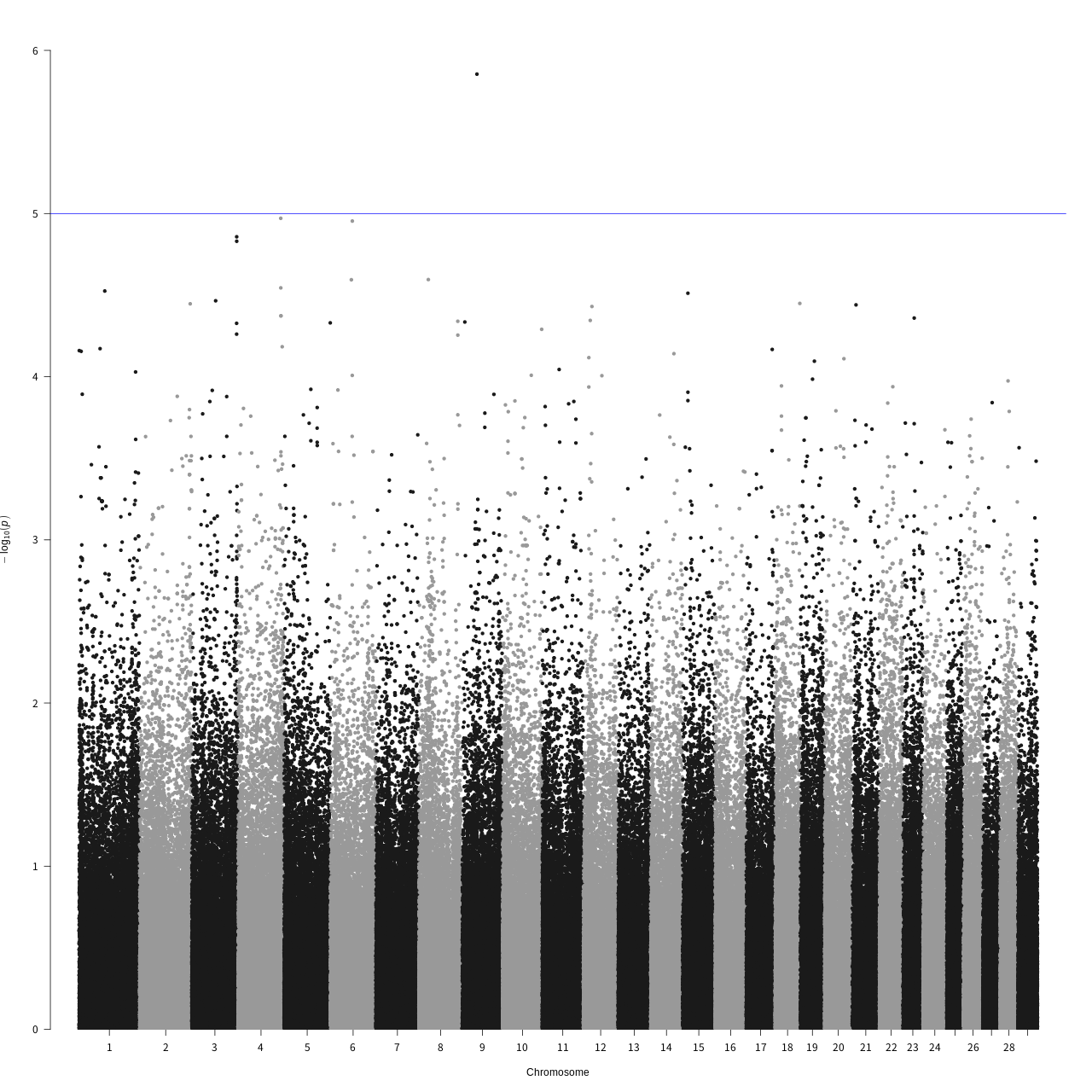

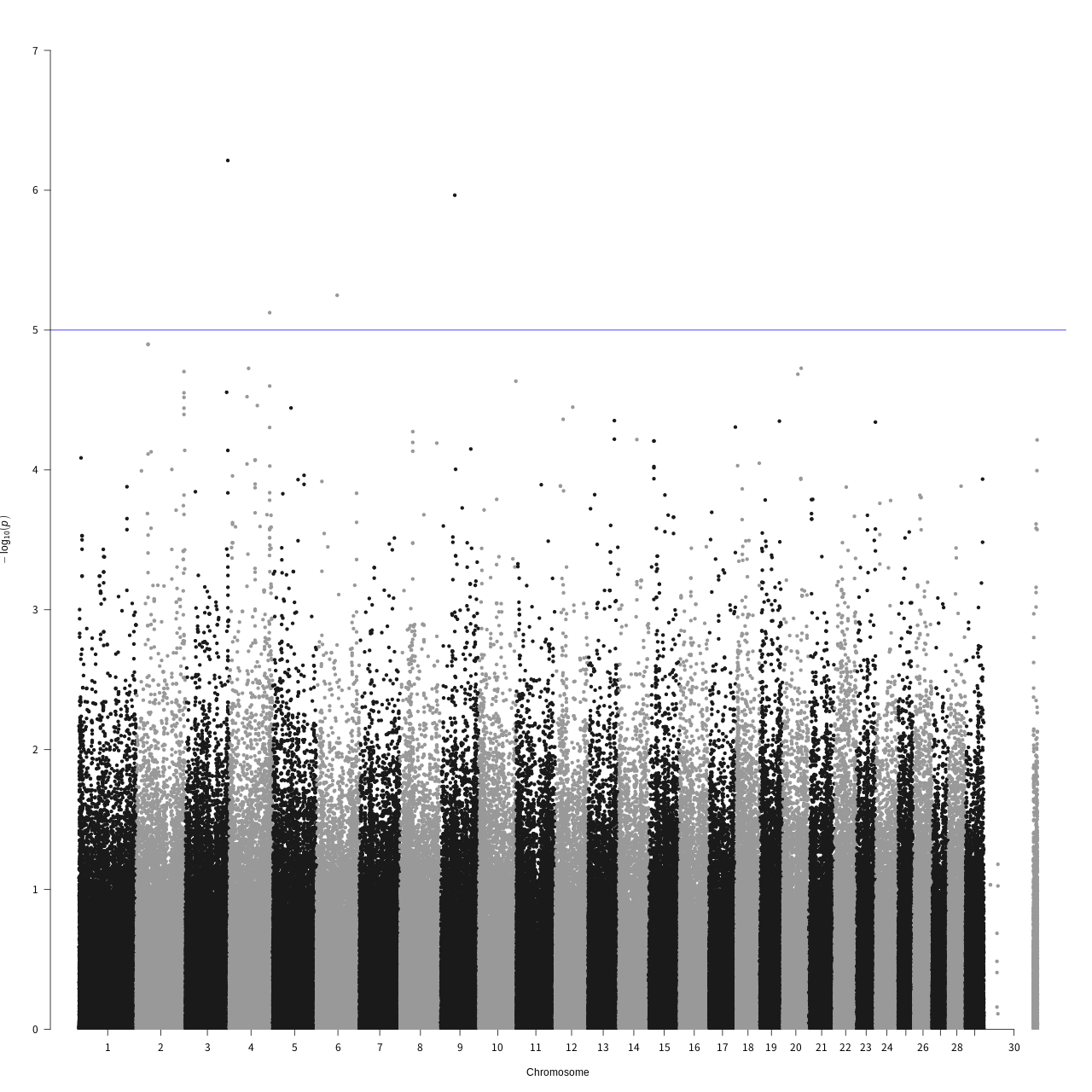

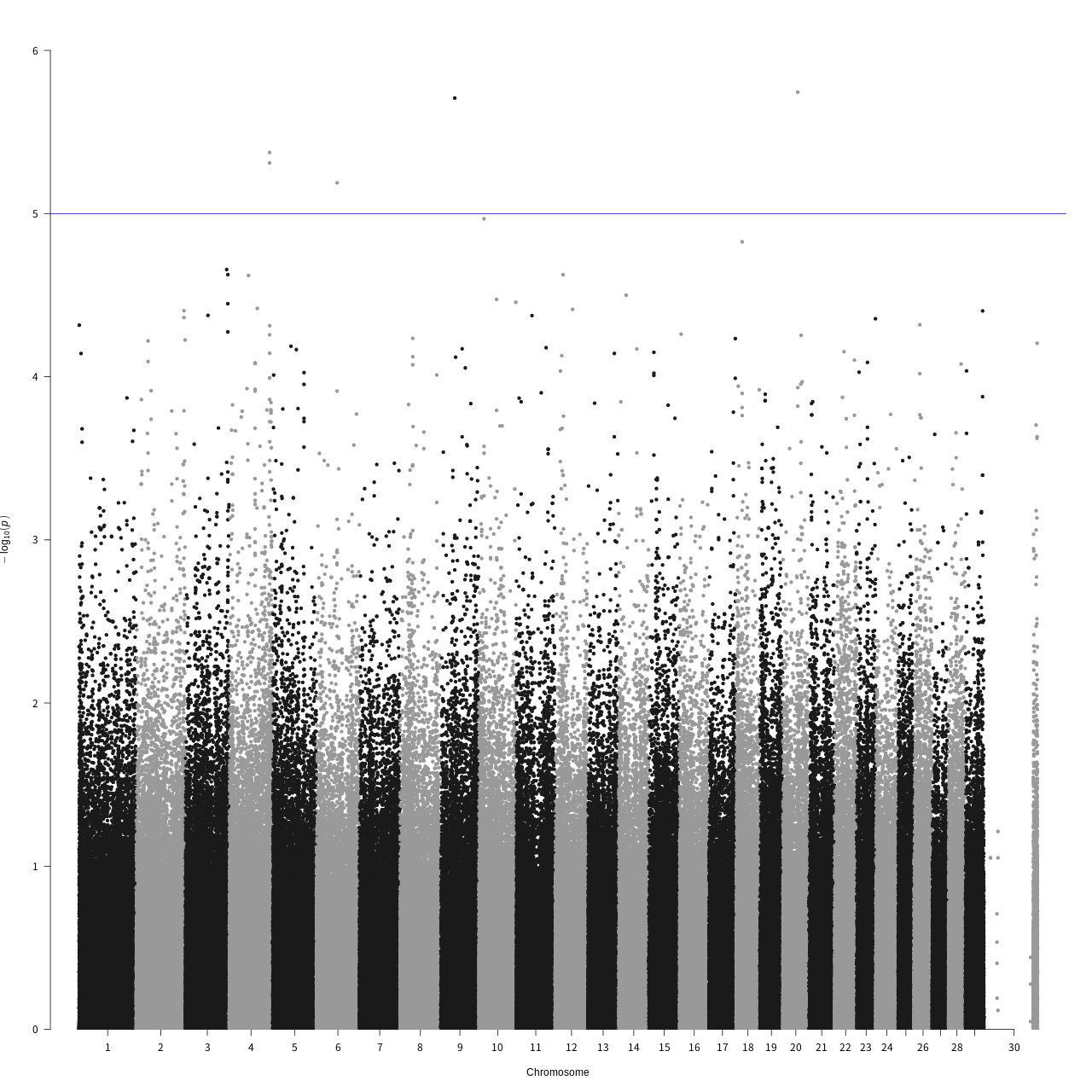

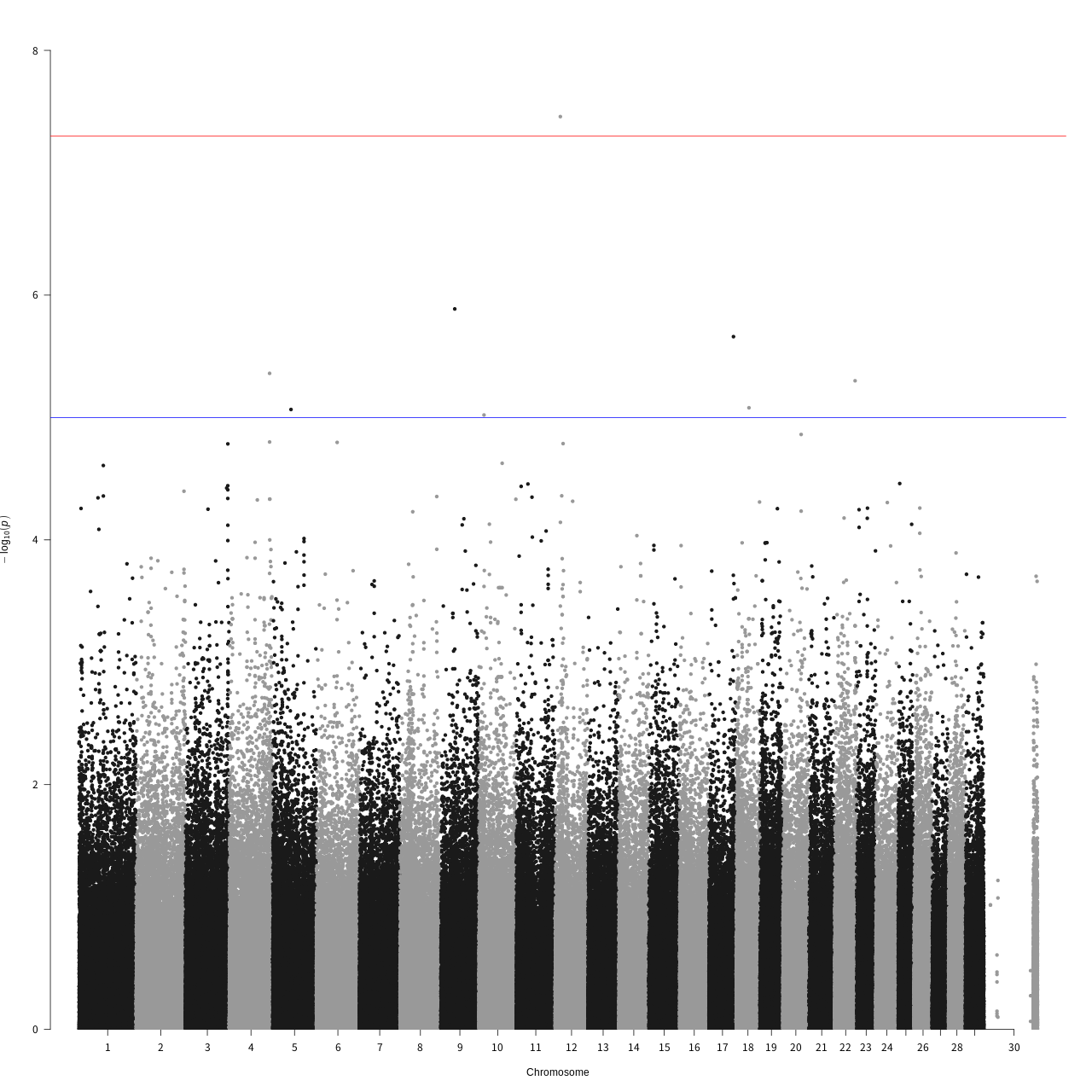


ms_len_5_gemma ms_len_10_gemma ms_len_20_gemma


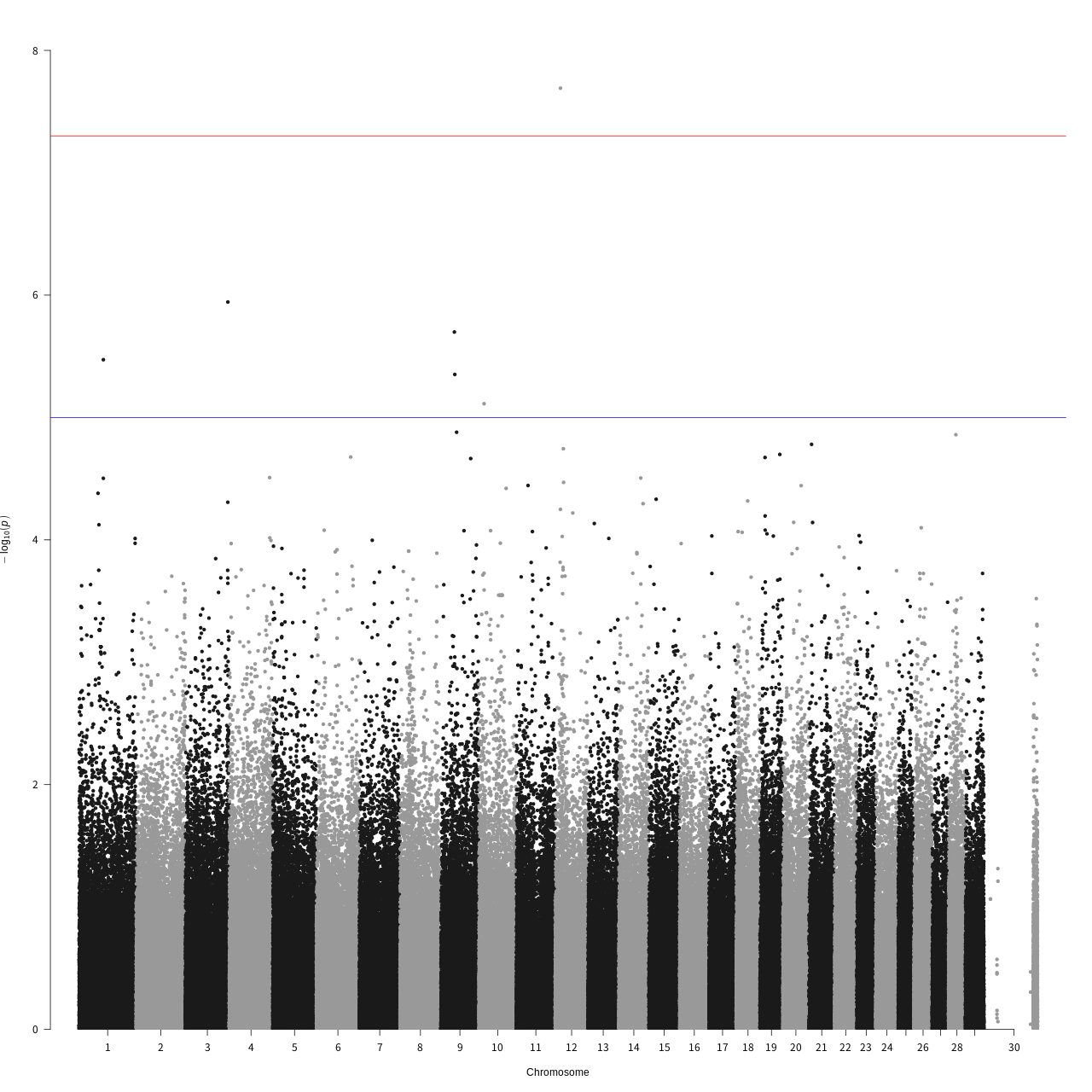

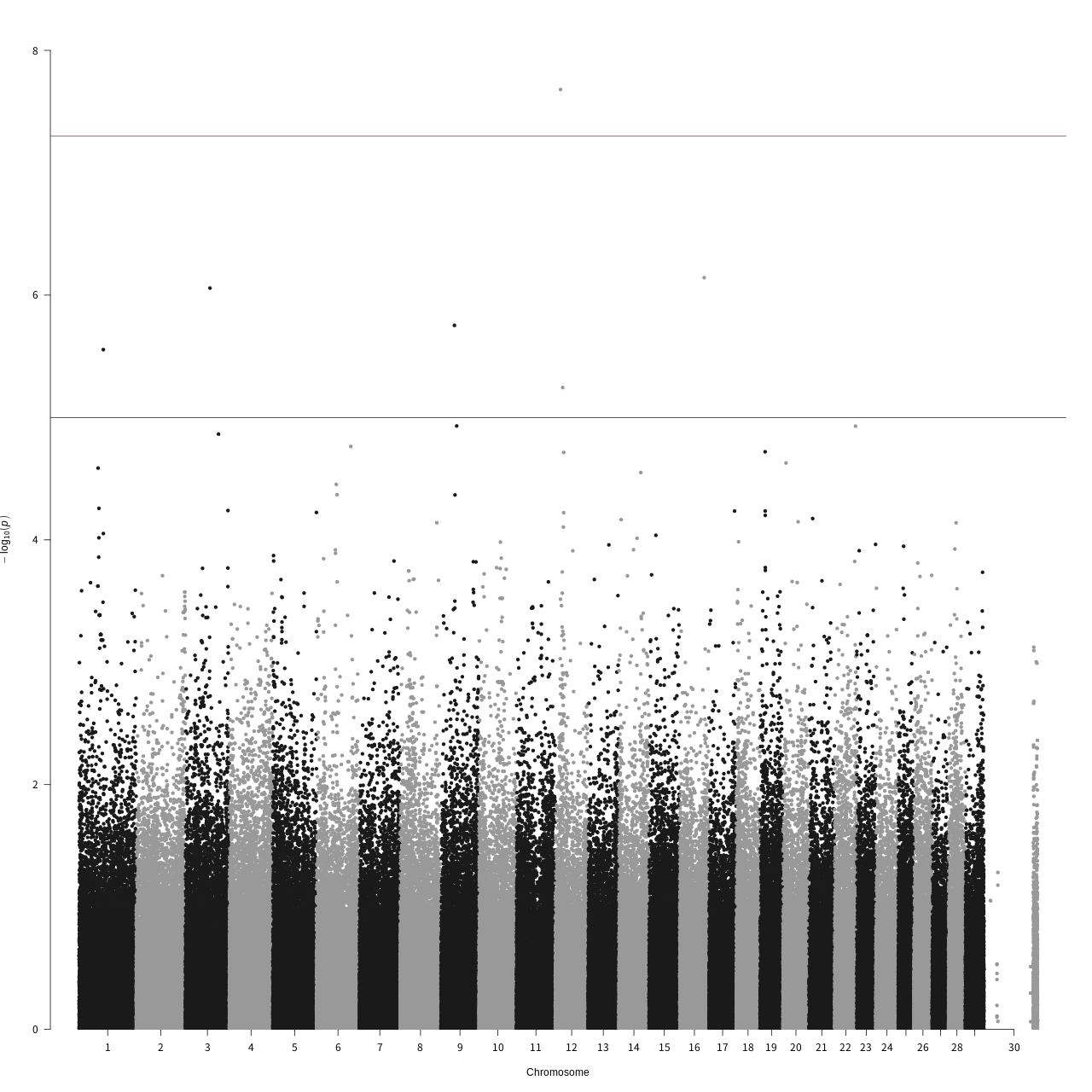

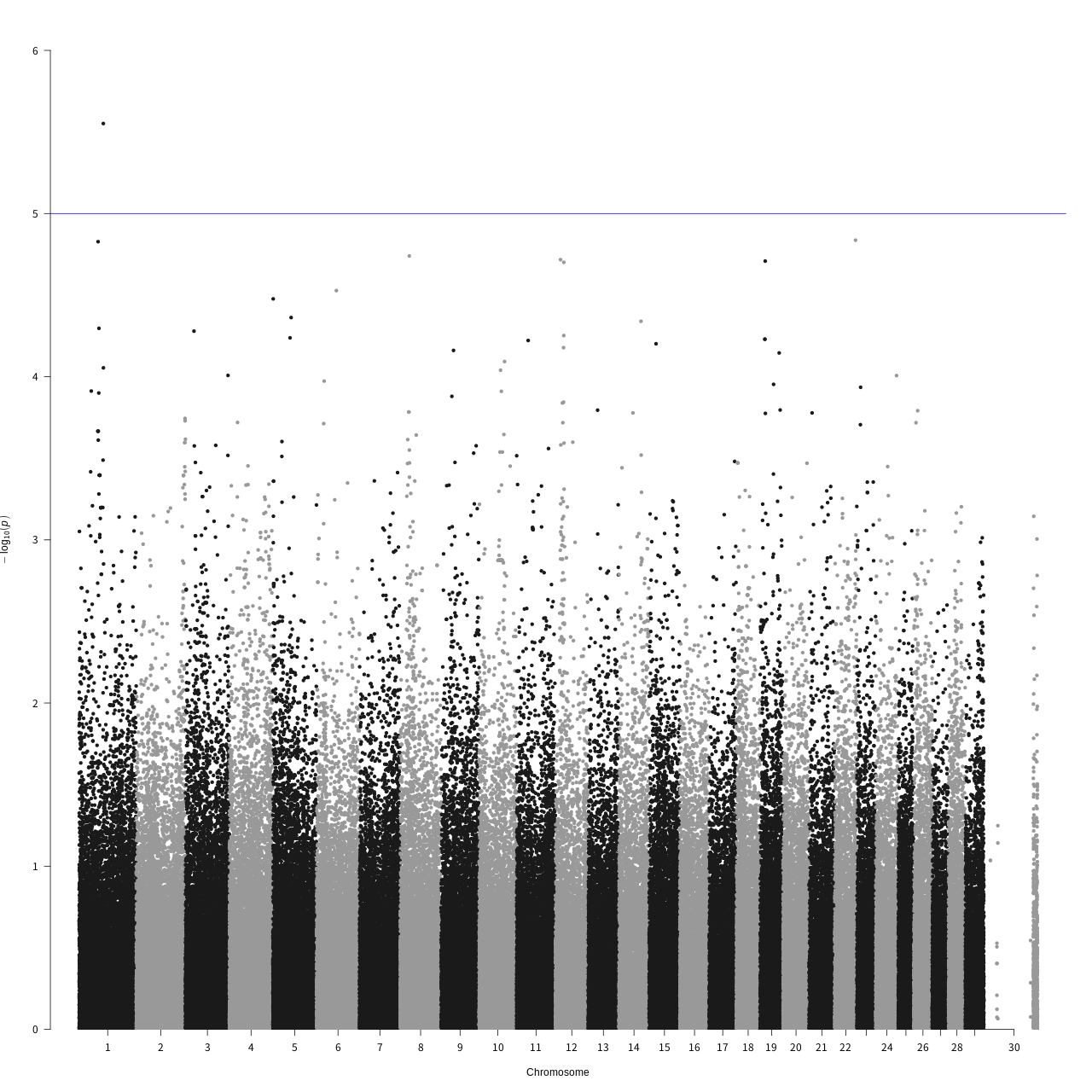


ms_len_50_gemma ms_len_100_gemma ms_len_200_gemma


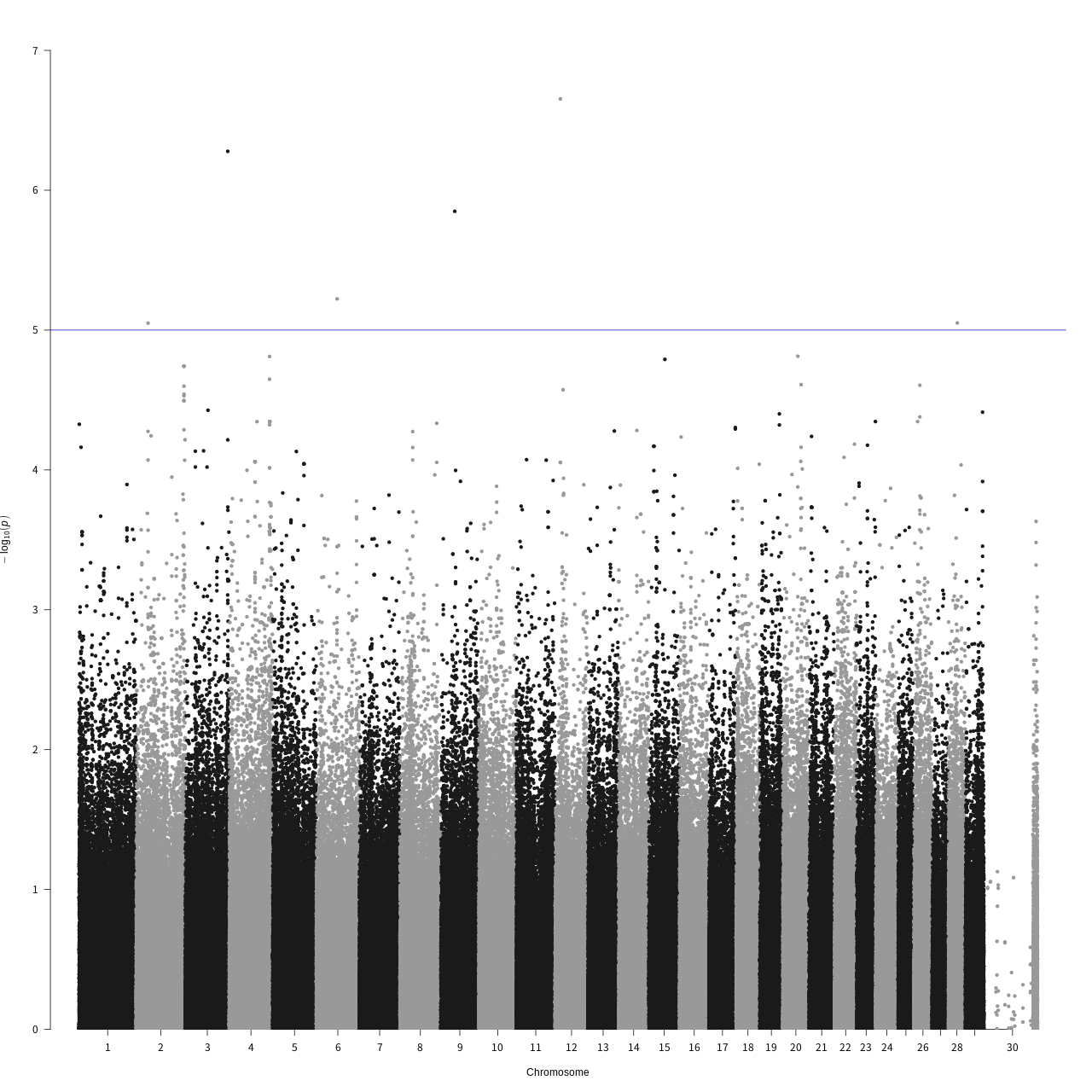

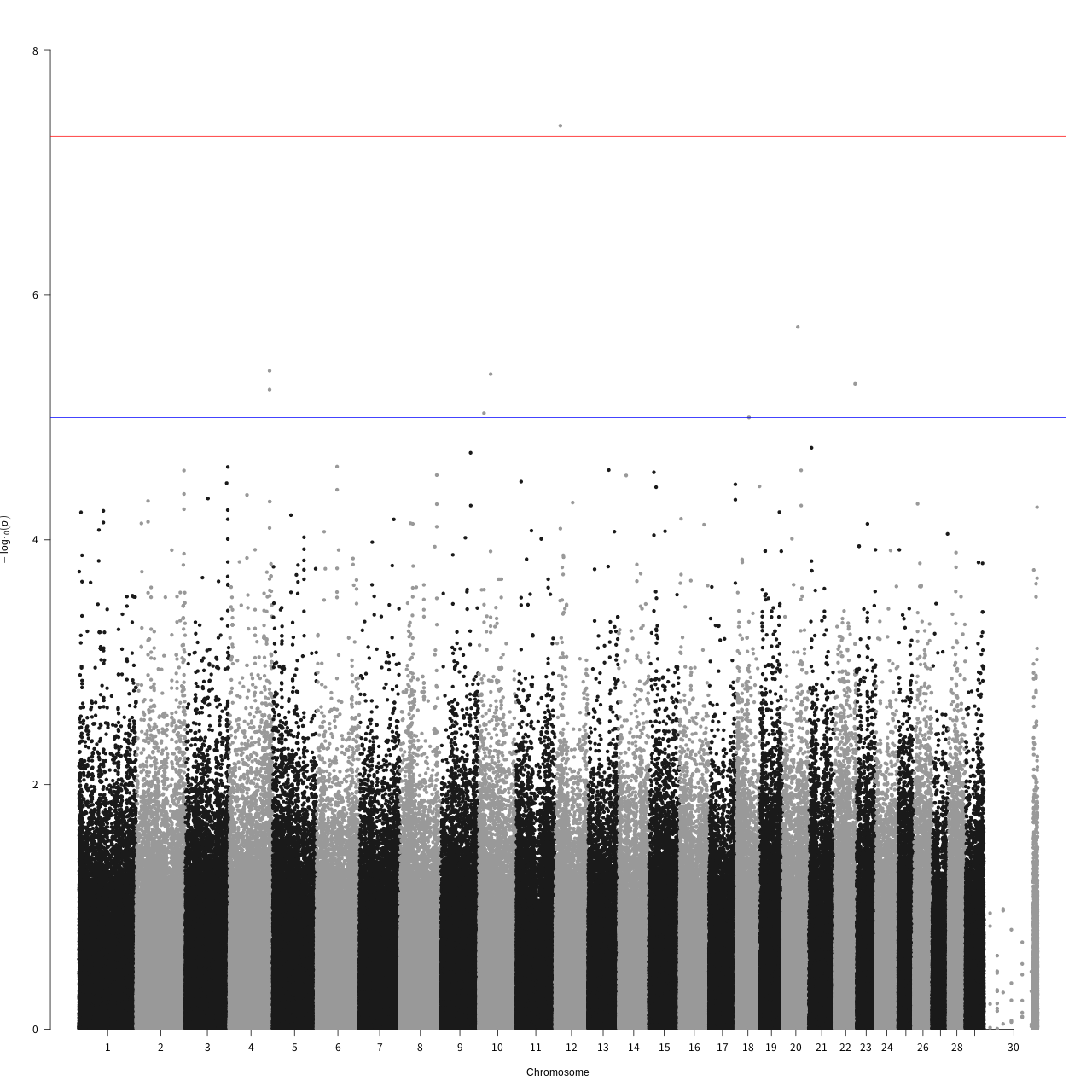

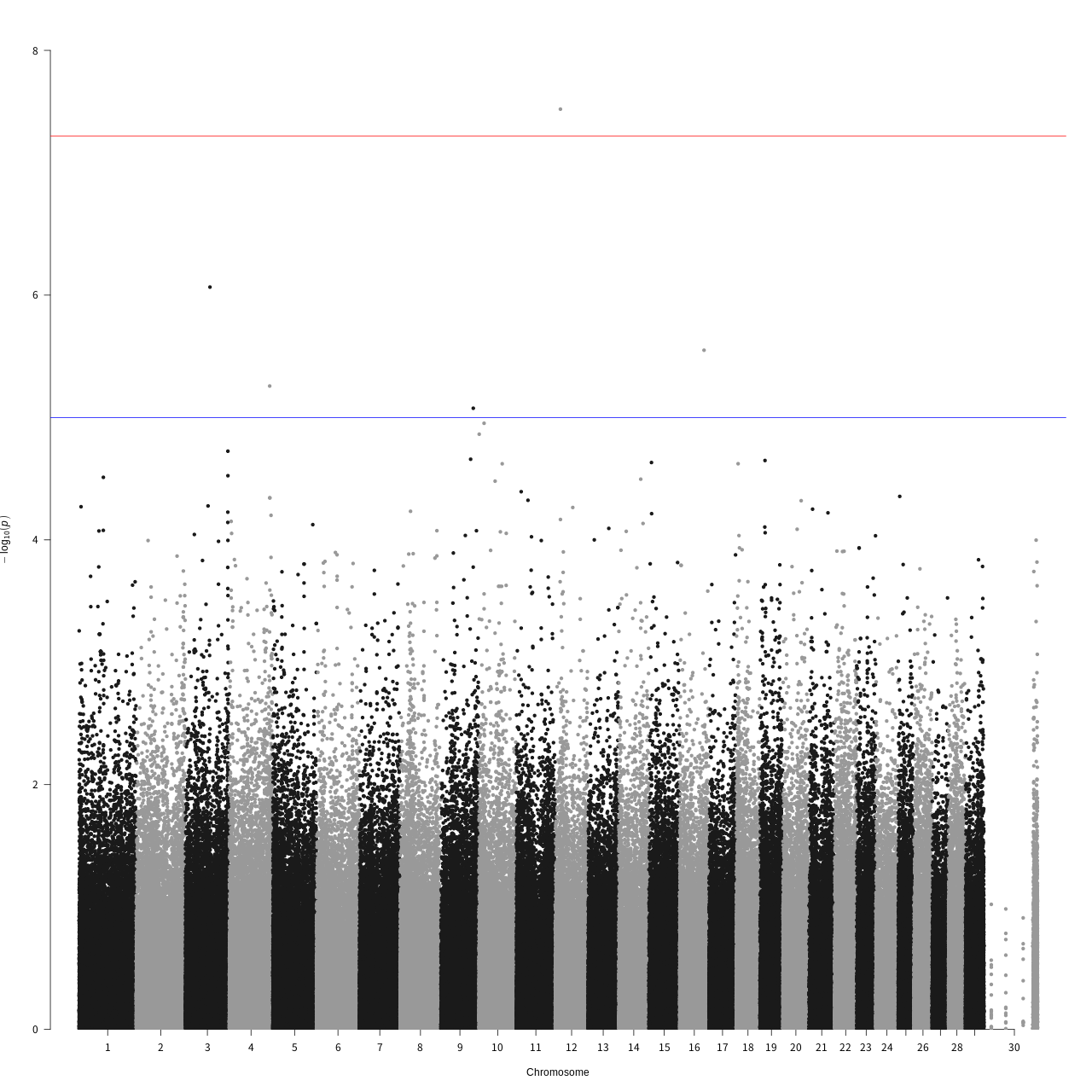


ms_nsnp_2_gemma ms_nsnp_5_gemma ms_nsnp_10_gemma


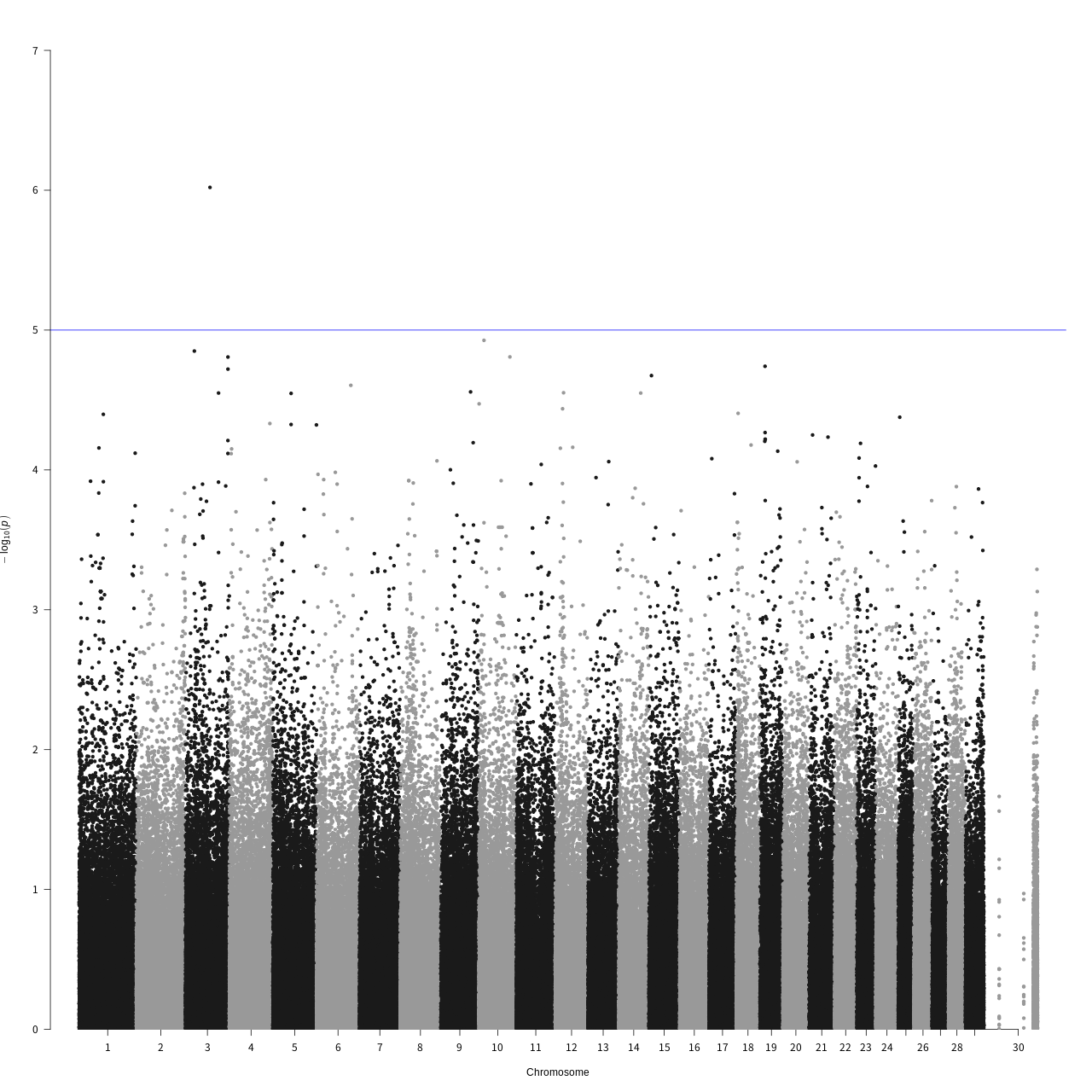

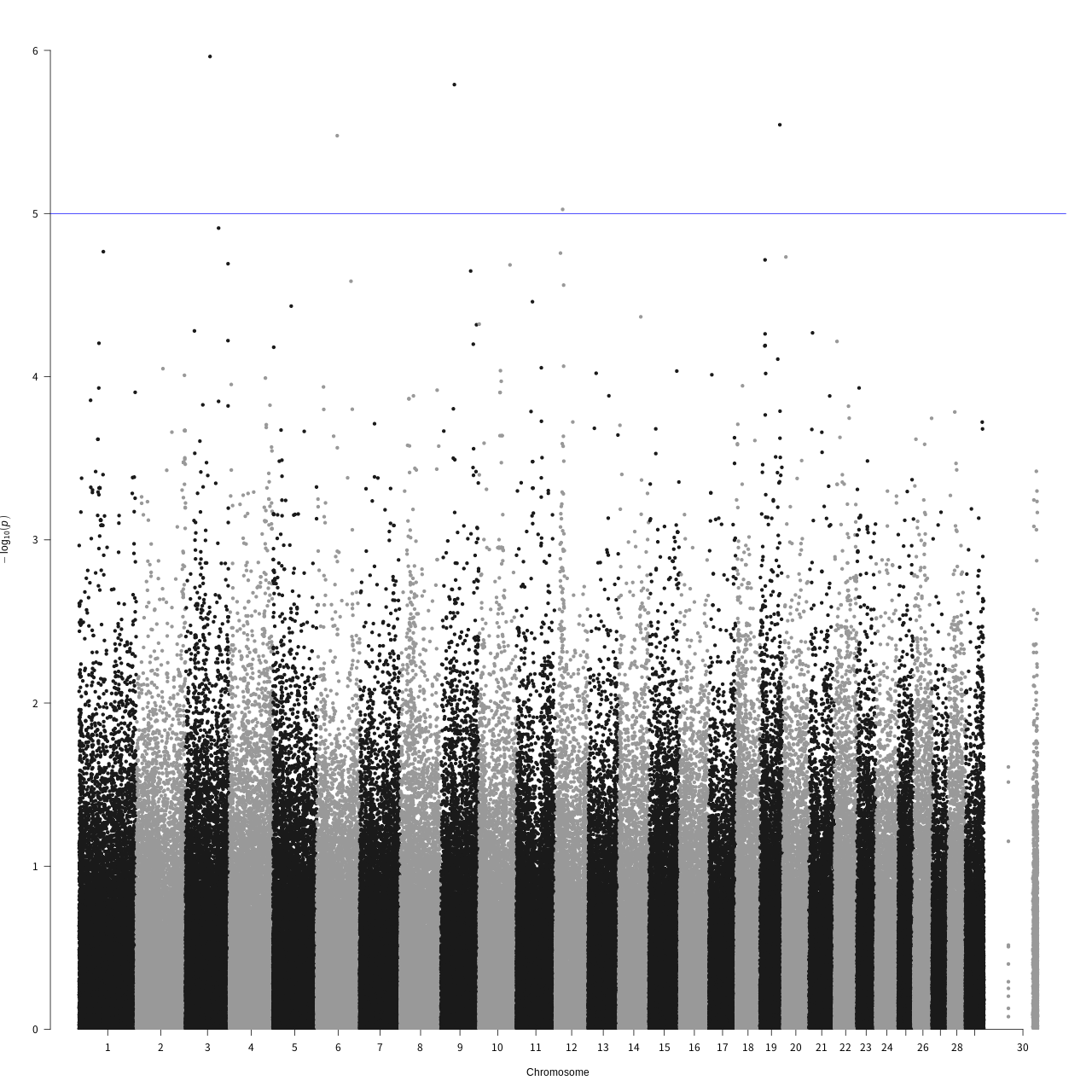

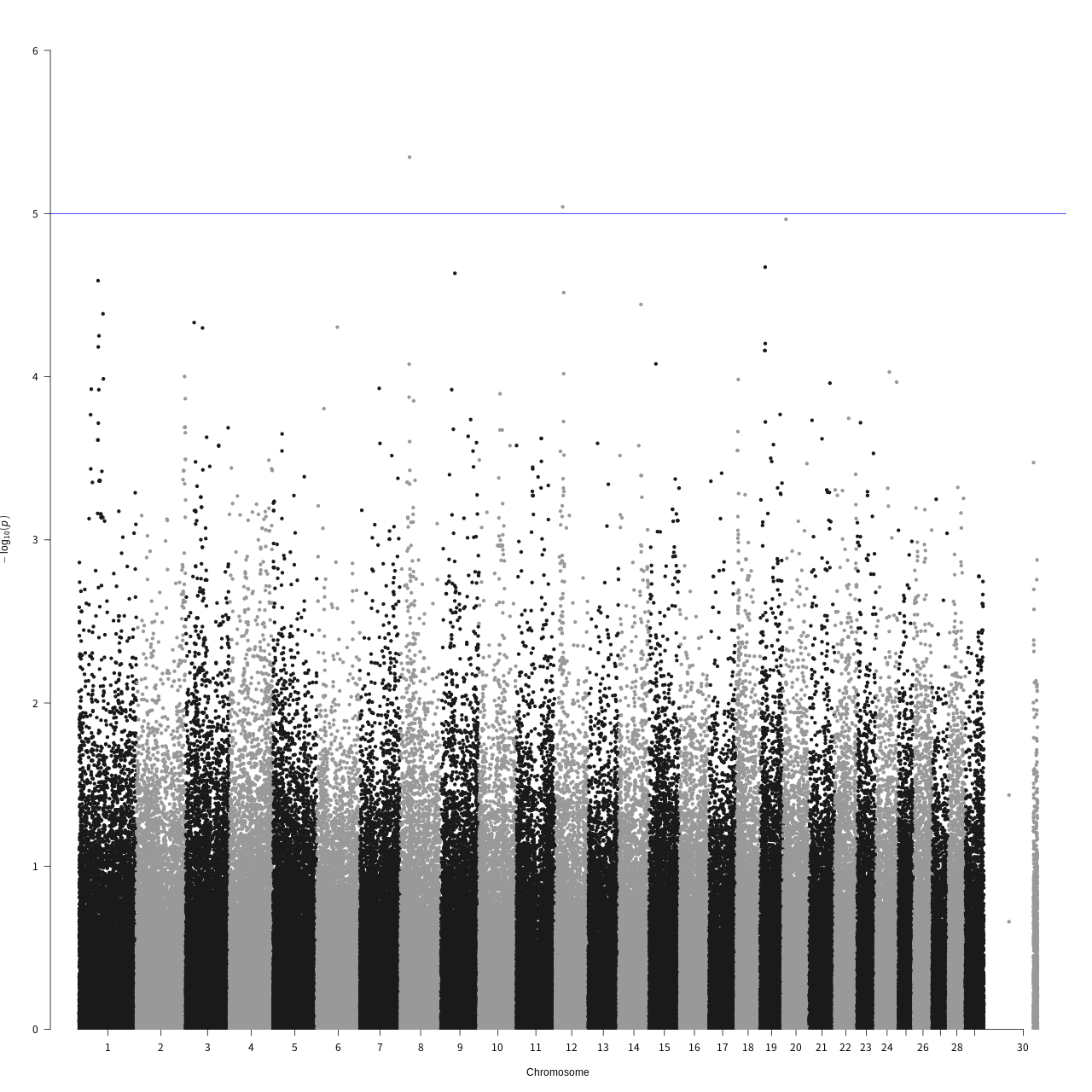


ms_nsnp_20_gemma ms_nsnp_30_gemma ms_nsnp_50_gemma
