# Supplementary material for: Haplotype-Based Genome-Wide Association Study and Identification of Candidate Genes Associated with Carcass Traits in Hanwoo Cattle
Source: Genes (Basel). 2020 May 14;11(5):551. doi: 10.3390/genes11050551 (PMC7290854; doi:10.3390/genes11050551)
Supplement: Supplementary file 1 [file genes-11-00551-s001.zip › Table S3.docx]

**Table S3: Common genes identified by all three methods: Len, LD and nsnp by VENNY**

**BFT**

5 common elements in "Len", "LD" and "nsnp"

GTF2H1

TASP1

TMX4

PLCB1

LRP8

4 common elements in "Len" and "nsnp":

VRK1

FAM110A

PLCB4

ISM1

9 common elements in "LD" and "nsnp":

LCN9

MAGOH

CZIB

CPT2

TRMT10A

RALGDS

GBGT1

PAEP

GLT6D1

**CWT**

45 common elements in "Len", "LD" and "nsnp":

LYPLA1

MRPL15

ANXA13

ASPH

ATP6V1H

C14H8orf34

CA8

CPA6

DHX15

IMPAD1

KHDRBS3

LACTB2

PREX2

PRKDC

SNTG1

SOX17

TOX

UBE2V2

ASAP1

HAS2

KCNQ3

LRATD2

RB1CC1

SPIDR

ST18

RP1

XKR9

ZFAT

CLVS1

FAM91A1

FBXO32

FER1L6

NCOA2

TMEM68

TGS1

KLHL38

RGS20

TCEA1

MCM4

H3F3C

MARVELD1

ZFYVE27

SFRP5

TRAM1

LRRC6

18 common elements in "Len" and "LD":

CYP7B1

PCDH7

PHF20L1

TG

ST3GAL1

CNTN5

LYN

PCMTD1

TTLL8

MLC1

MOV10L1

TBC1D31

FAM83A

DERL1

ZHX2

MORN4

PI4K2A

AVPI1

**EMA**

5 common elements in "Len", "LD" and "nsnp":

SLC8A3

COX16

PDE10A

ADAM21

MED6

1 common element in "Len" and "LD":

SYNJ2BP

1 common element in "Len" and "LD":

SYNJ2BP

**MS**

4 common elements in "Len" and "nsnp":

SCLY

EZH2

ST6GALNAC5

FAM124A

1 common element in "Len", "LD" and "nsnp":

FYN

4 common elements in "LD" and "nsnp":

PGM2

PRPSAP1

QRICH2

UBALD2

1 common element in "Len" and "LD":

IQCH
