# Supplementary material for: Haplotype-Based Genome-Wide Association Study and Identification of Candidate Genes Associated with Carcass Traits in Hanwoo Cattle
Source: Genes (Basel). 2020 May 14;11(5):551. doi: 10.3390/genes11050551 (PMC7290854; doi:10.3390/genes11050551)
Supplement: Supplementary file 1 [file genes-11-00551-s001.zip › Table S4.docx]

**Table S4: DAVID functional annotation cluster for genes significant for carcass trait and their pathways**

| **Category** | **Term** | **Count** | **%** | ***p-value*** | **Genes** |
| --- | --- | --- | --- | --- | --- |
| **BFT (Back Fat thickness)** | | | | | |
| GO_BP | GO:0016042~lipid catabolic process | 3 | 4.76 | 0.020 | *CEL, PLCB4, PLCB1* |
| KEGG | bta04911:Insulin secretion | 3 | 4.76 | 0.030 | *PLCB4, PLCB1, SNAP25* |
| KEGG | bta03015:mRNA surveillance pathway | 3 | 4.76 | 0.035 | *UPF2, MAGOH, MSI1* |
| KEGG | bta04922:Glucagon signaling pathway | 3 | 4.76 | 0.039 | *LDHA, PLCB4, PLCB1* |
| GO_BP | GO:0005975~carbohydrate metabolic process | 3 | 4.76 | 0.039 | *LDHA, GBGT1, GLT6D1* |
| KEGG | bta04972:Pancreatic secretion | 3 | 4.76 | 0.040 | *CEL, PLCB4, PLCB1* |
| KEGG | bta01100:Metabolic pathways | 9 | 14.29 | 0.040 | *CEL, LDHA, DHFR, PLCB4, GBGT1, SPTLC3, AOX4, PLCB1, NANP* |
| KEGG | bta04724:Glutamatergic synapse | 3 | 4.76 | 0.053 | *PLCB4, SLC1A7, PLCB1* |
| KEGG | bta04071:Sphingolipid signaling pathway | 3 | 4.76 | 0.059 | *PLCB4, SPTLC3, PLCB1* |
| **Carcass Weight (CWT)** | | | | | |
| GO_BP | GO:0031648~protein destabilization | 4 | 3.92 | 0.001 | *DERL1, RNF139, PRKDC, SOX17* |
| KEGG | bta04110:Cell cycle | 4 | 3.92 | 0.021 | *PRKDC, CDK6, MCM4, MYC* |
| GO_BP | GO:0071805~potassium ion trans membrane transport | 3 | 2.94 | 0.034 | *KCNQ3, KCNB2, KCNIP4* |
| GO_BP | GO:0071498~cellular response to fluid shear stress | 2 | 1.96 | 0.051 | *MTSS1, HAS2* |
| GO_BP | GO:0060070~canonical Wnt signaling pathway | 3 | 2.94 | 0.059 | *SFRP5, SOX17, MYC* |
| GO_BP | GO:0034765~regulation of ion trans membrane transport | 2 | 1.96 | 0.089 | *KCNQ3, KCNB2* |
| GO_BP | GO:0090090~negative regulation of canonical Wnt signaling pathway | 3 | 2.94 | 0.090 | *SFRP5, RGS20, SOX17* |
| **EMA (Eye Muscle Area)** | | | | | |
| GO_BP | GO:0002092~positive regulation of receptor internalization | 3 | 6.25 | 0.001 | *SCYL2, WNT3A, SYNJ2BP* |
| GO_BP | GO:0021766~hippocampus development | 3 | 6.25 | 0.003 | *WNT3A, PAFAH1B1, YWHAE* |
| GO_BP | GO:0010988~regulation of low-density lipoprotein particle clearance | 2 | 4.17 | 0.008 | *CNPY2, NR1H4* |
| GO_BP | GO:2000188~regulation of cholesterol homeostasis | 2 | 4.17 | 0.018 | *RORA, NR1H4* |
| GO_BP | GO:0070507~regulation of microtubule cytoskeleton organization | 2 | 4.17 | 0.037 | *WNT3A, PAFAH1B1* |
| GO_BP | GO:0006605~protein targeting | 2 | 4.17 | 0.040 | *SYNJ2BP, YWHAE* |
| GO_BP | GO:0043124~negative regulation of I-kappaB kinase/NF-kappaB signaling | 2 | 4.17 | 0.078 | *RORA, NR1H4* |
| GO_BP | GO:0043087~regulation of GTPase activity | 2 | 4.17 | 0.083 | *PAFAH1B1, CRK* |
| GO_BP | GO:0045599~negative regulation of fat cell differentiation | 2 | 4.17 | 0.083 | *WNT3A, RORA* |
| **MS (Marbling Score)** | | | | | |
| KEGG | bta04110:Cell cycle | 3 | 8.57 | 0.022 | *RB1, CHEK2, CUL1* |
| GO_BP | GO:2000134~negative regulation of G1/S transition of mitotic cell cycle | 2 | 5.71 | 0.024 | *EZH2, RB1* |
| GO_BP | GO:0006915~apoptotic process | 3 | 8.57 | 0.031 | *RRAGA, RB1, CUL1* |
| GO_BP | GO:0042752~regulation of circadian rhythm | 2 | 5.71 | 0.043 | *EZH2, PPARG* |
