# Supplementary figures and images for: Haplotype-Based Genome-Wide Association Study and Identification of Candidate Genes Associated with Carcass Traits in Hanwoo Cattle
Source: Genes (Basel). 2020 May 14;11(5):551. doi: 10.3390/genes11050551 (PMC7290854; doi:10.3390/genes11050551)

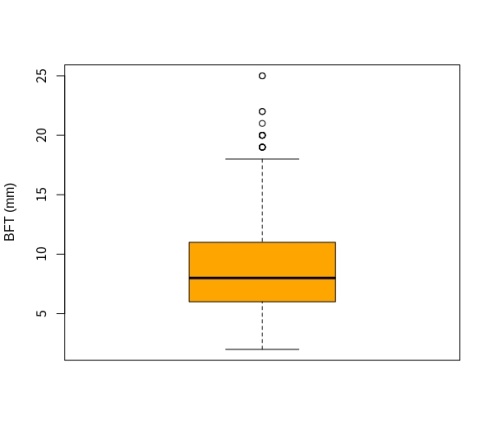

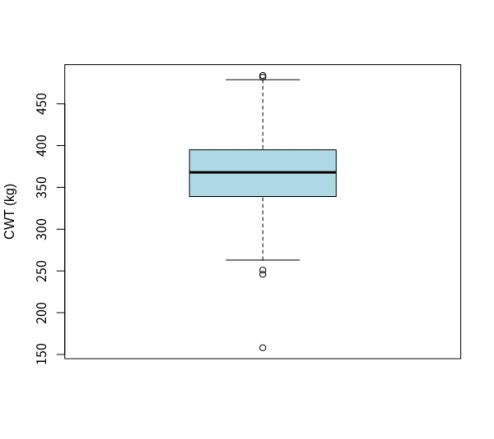


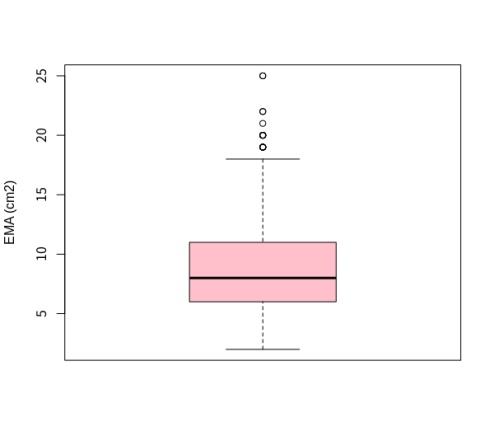

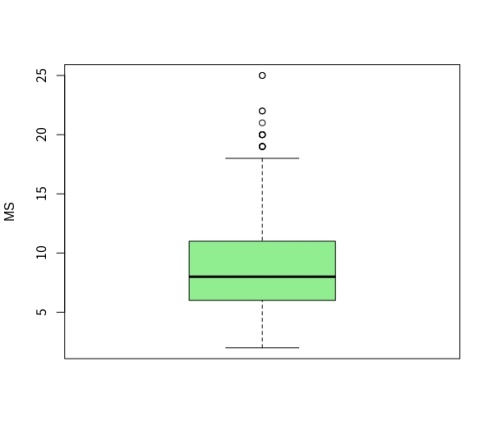


Figure S1: Boxplot for phenotype used in this study

Supplement: Supplementary file 1 [file genes-11-00551-s001.zip › Figure S1.docx]
